# Alkyne Nitro Tag Enables Stable and Efficient Protein Functionalization of Gold Nanoparticles

**DOI:** 10.1021/acsami.6c01215

**Published:** 2026-03-04

**Authors:** Shun-Qiang Xu, Yung-Kun Pan, Po-Cheng Lin, Ling-Ling Weng, Tzu-Jung Chang, Chien-Chi Wu, Yun-Rong Peng, Kui-Thong Tan

**Affiliations:** † Department of Chemistry, 34881 National Tsing Hua University, 101 Section 2, Kuang Fu Road, Hsinchu 30013, Taiwan, Republic of China; ‡ Department of Medicinal and Applied Chemistry, Kaohsiung Medical University, Kaohsiung 80708, Taiwan, Republic of China

**Keywords:** gold nanoparticles, surface modification, protein functionalization, alkyne, nitrobenzene

## Abstract

Surface ligands play a critical role in the preparation of stable, covalently bound nanoparticle–protein conjugates. However, large surface ligands often introduce steric and antifouling effects that reduce protein conjugation yields, whereas small ligands tend to induce nanoparticle aggregation during activation. Here, we report Alkyne Nitro Tag (**ANT**), a compact heterobifunctional ligand that represents a design principle for nanoparticle surface chemistry. **ANT** presents a preinstalled reactive nitrophenyl ester that undergoes nucleophilic acyl substitution with protein residues under mild aqueous conditions, while its nitro substituent imparts colloidal stability to AuNPs through electrostatic repulsion. In **ANT**, the terminal alkyne is essential for strong attachment to the AuNP surface, as thiol groups are incompatible with the reactive ester. Robust conjugation of **ANT**-capped AuNPs (**Au@ANT**) with streptavidin (SA), horseradish peroxidase (HRP), and immunoglobulin G (IgG) was demonstrated by lateral flow assays, Western blotting, enzymatic sensing, and immunoprecipitation. Compared to the conventional physical adsorption and EDC/NHS chemistry, **Au@ANT** conjugates consistently exhibited higher yields, improved stability under physiological and denaturing conditions, and greater retention of protein activity. These results establish **ANT** as a generalizable and high-performance strategy for generating stable and active AuNP–protein conjugates, offering significant advantages for nanomedicine and advanced materials science.

## Graphical abstract

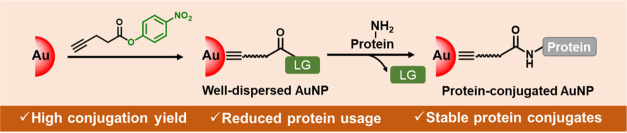

## Introduction

Gold nanoparticles (AuNPs) are widely used in medicine, biology, and chemistry due to their tunable surface plasmon resonance, biocompatibility, and large surface-to-volume ratio, making them ideal scaffolds for catalysts, sensors, and therapeutic agents.^1–3^ Achieving these applications requires robust surface functionalization of AuNPs with biomolecules, such as proteins, peptides, and oligonucleotides.

In general, biomolecule immobilization on AuNPs can be achieved through either physical adsorption (PA) or covalent chemical conjugation.^4^ PA offers a straightforward, coupling-agent-free approach that relies on either electrostatic forces, hydrophobic interactions, or direct surface binding through cysteine residues on the protein (Figure 1a). Typically, PA is performed using citrate-capped AuNPs (**Au@Citrate**), where the solution pH is adjusted to match or slightly exceed the isoelectric point (pI) of the target protein to facilitate adsorption. To date, citrate capping remains the preferred choice for PA-based conjugation due to its simplicity and higher protein conjugation efficiency. However, PA typically requires high protein concentrations to effectively passivate the AuNP surface and prevent aggregation.^5,6^ At low protein concentrations, the rate of protein adsorption is insufficient to outcompete the displacement of citrate by salts in the solution, resulting in loss of surface stabilization and nanoparticle aggregation. Moreover, the success of PA-based conjugation often depends on pH optimization for each specific antibody, and some antibodies fail to form stable conjugates at any pH via direct adsorption.^7,8^ Additionally, weakly bound proteins may desorb from the AuNP surface, resulting in signal loss and compromising assay reliability in complex environments.^9,10^

**Figure 1:**
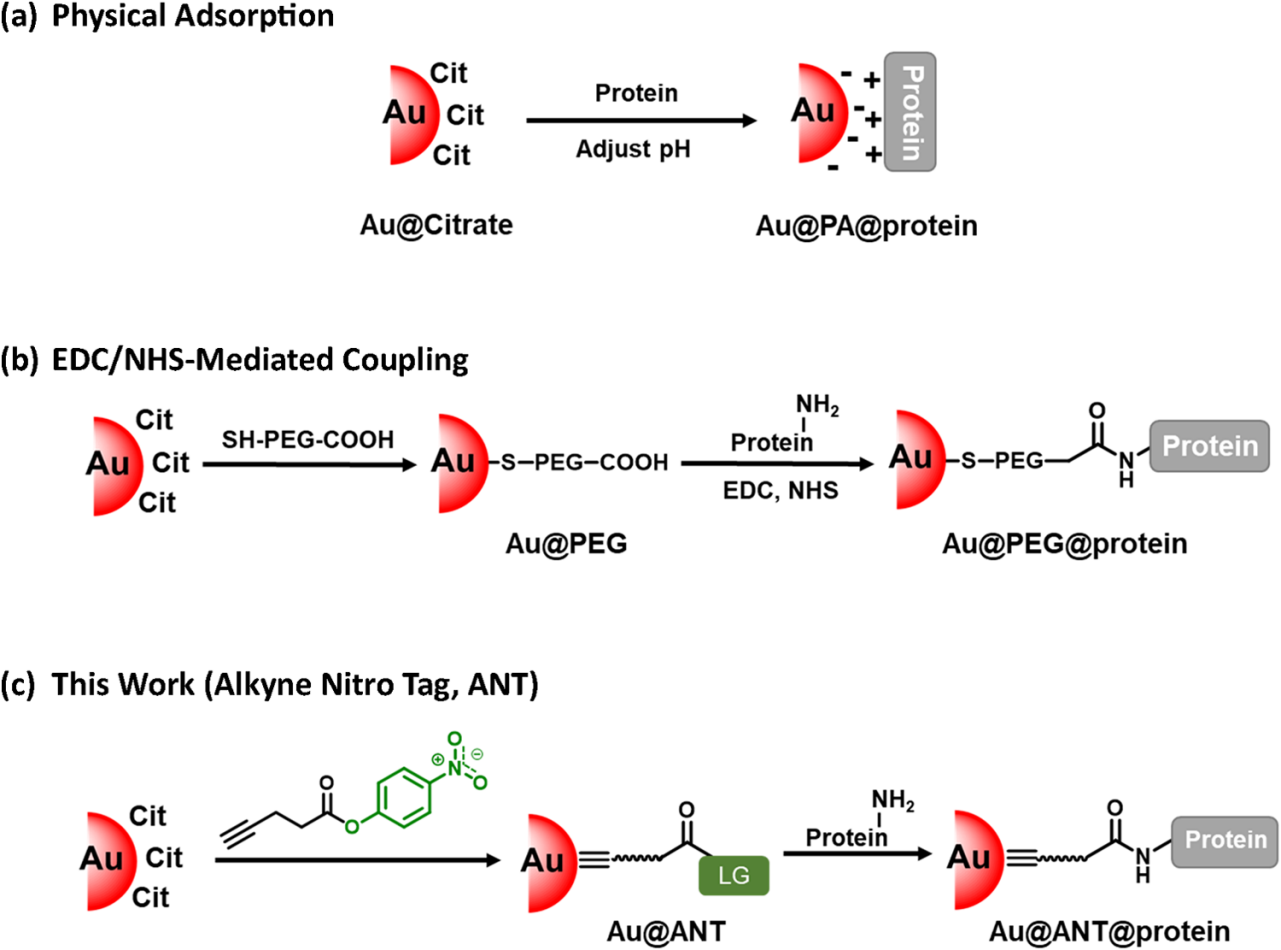
Schematic illustration of **Au@Protein** formation using citrate-capped AuNPs (**Au@Citrate**) by three different strategies. (a) physical adsorption, (b) EDC/NHS-mediated amide coupling, and (c) Alkyne Nitro Tag.

Covalent chemical conjugation methods for AuNP functionalization typically employ thiolated capping reagents that anchor to the gold surface while presenting terminal functional groups, such as carboxylic acids, azides, maleimides, tyrosine residues or Ni^2+^-nitrilotriacetic acid (NTA), to facilitate protein conjugation.^11–18^ These terminal groups enable a variety of bioconjugation strategies, including peptide coupling, click chemistry, Michael addition, enzyme-mediated reactions, and related approaches. Among these, amide-bond formation between protein amines and carboxyl-functionalized AuNP surfaces is the most commonly used, as it allows for the immobilization of a wide range of native proteins (Figure 1b). This is typically achieved via EDC/NHS-mediated carboxyl activation. However, activation of surface carboxylates often disrupts colloidal stability by reducing electrostatic repulsion, leading to unintended nanoparticle aggregation.^19–22^ To mitigate this, carboxylated thiol–poly­(ethylene glycol) (SH-PEG-COOH) is frequently used to maintain stability during conjugation. Yet, the antifouling nature of PEG can hinder protein access to the nanoparticle surface, thereby reducing coupling efficiency.^23–25^ Moreover, NHS esters, while highly reactive, are prone to rapid hydrolysis, which limits the effective coupling window.^26^ At pH 7 and room temperature, NHS esters hydrolyze significantly faster (*t*_1/2_ ≈ 40.6 min) than nitrophenyl esters (*t*_1/2_ ≈ 7.6 h), making them less stable in aqueous solution.^27–29^ Although a one-pot setup involving carboxylated AuNPs, proteins, and EDC/NHS can partially circumvent this issue, such reactions often require fine-tuning and may result in undesired interprotein cross-linking.^30^ Additionally, activation of carboxylic acids near adjacent carboxyl groups can lead to intramolecular cyclization, forming cyclic anhydrides that increase surface hydrophobicity, reduce water solubility, and ultimately impair conjugation efficiency despite the higher inherent reactivity.^31^ These limitations underscore the continued need for more robust and reliable protein conjugation strategies for AuNP surface functionalization.

To address these limitations, we introduce Alkyne Nitro Tag (**ANT**), a compact heterobifunctional ligand that enables efficient and stable covalent conjugation of native proteins to AuNPs (Figure 1c). **ANT** incorporates a preinstalled reactive nitrophenyl ester moiety, which can undergo nucleophilic acyl substitution with nucleophilic residues on proteins under mild aqueous conditions, eliminating the need for in situ activation steps that often result in aggregation and low conjugation yields. For anchoring to the AuNP surface, **ANT** employs a terminal alkyne group that is inert to electrophilic attack yet capable of forming strong covalent interactions with gold.^32–35^ Although traditional heterobifunctional cross-linkers such as DTSSP and DSNB have been widely used for antibody–AuNP conjugation via NHS ester–amine chemistry, their effectiveness is limited by the rapid hydrolysis of NHS esters, which often outcompetes aminolysis and necessitates large excesses of protein.^36,37^ Recent reports further suggest that DTSSP may hydrolyze before covalent coupling occurs, resulting in protein adsorption rather than true covalent attachment.^7,26,38^ It is also noteworthy that most neutral small-molecule ligands fail to stabilize AuNPs and often induce aggregation.^39–43^ In contrast, **ANT** confers remarkable colloidal stability despite its compact and overall neutral structure. This stability likely attributed to the formal negative charge of the oxygen atom in the nitro group, which may contribute to electrostatic or dipolar repulsion. Such localized negative character on the nitro group could influence interparticle interactions, particularly in systems where traditional small neutral ligands often induce aggregation. **ANT**’s small size and moderate hydrophobicity may facilitate protein adsorption to the AuNP surface, enhancing conjugation efficiency through proximity effect.^25,44^ This compact design not only improves conjugation efficiency under mild aqueous conditions but also preserves protein activity and prevents desorption, effectively addressing the key limitations of both physical adsorption and traditional EDC/NHS-based methods.

Here, we demonstrate the utility of **ANT** for functionalizing AuNPs with a range of proteins, including streptavidin (SA), horseradish peroxidase (HRP), and immunoglobulin G (IgG), achieving higher conjugation yields and enhanced stability compared to conventional methods. The versatility of **ANT**-functionalized AuNPs was further validated across multiple analytical methods and applications, including lateral flow assays, dot blotting, Western blotting, enzymatic activity assays, and colorimetric sensing. These results establish **ANT** as a robust and broadly applicable strategy for stable and efficient protein conjugation to AuNPs.

## Results and Discussion

### Design Rationale and Colloidal Stability of **Au@ANT**

To justify the use of terminal alkynes rather than thiols for AuNP surface functionalization, we first compared their chemical stability in the presence of electrophiles. While thiolated Nitro Tag (**SNT**) degraded within 3 days in DMSO at −80 °C, **ANT** retained its original HPLC profile even after 1 year (Figure S1), confirming the superior compatibility of terminal alkynes over thiols in preserving preactivated groups. In addition, the nitrophenyl ester moiety in **ANT** plays dual roles: it contributes to electrostatic repulsion that stabilizes the colloid and provides a reactive site for nucleophilic attack by biomolecules. In this study, we employed **ANT** to functionalize AuNPs with native proteins, generating **Au@ANT@protein**, and compared its performance with **Au@PA@protein** and **Au@CO**_**2**_ **H@protein**, prepared using conventional PA and EDC/NHS methods, respectively (Figure 1).

For surface functionalization, 100 μM **ANT** was incubated with **Au@citrate** at 25 °C in pH 7 aqueous solution for 4 h to produce **Au@ANT**. After centrifugation and washing, **Au@ANT** retained its characteristic wine-red color, with its maximum absorption peak shifting slightly from 521 to 524 nm, similar to SH-PEG­(1K)-COOH-modified AuNPs (**Au@PEG**) (Figure 2a,2b, and Table 1). In contrast, ligands lacking the nitro group, such as phenyl ester (ligand **2**), NHS (ligand **3**), and difluorophenyl ester (ligand **4**), resulted in rapid color changes to blue, accompanied by broad and significantly red-shifted absorption spectra, indicative of nanoparticle aggregation. This aggregation is attributed to rapid displacement of citrate by alkynylated ligands, leading to the loss of electrostatic stabilization. Similarly, other short alkynylated ligands containing sulfonic acid, thioester, hydroxyl, or primary amine groups also failed to maintain the wine-red color, further confirming colloidal destabilization (Figure S2). Notably, only nitro-containing ligands (**ANT**, ligands **5** and **10**) and carboxylic acid–containing ligands (MUA, MPA, PTA, and SH-PEG­(1K)-COOH) preserved the colloidal appearance of the AuNPs. Among these, the nitro-containing ligands uniquely stabilized the particles without the need for charged groups or bulky polymers, a novel behavior not previously reported. Finally, to demonstrate that terminal alkynes can anchor onto AuNP surfaces with efficiency comparable to thiols, the surface coverage of **ANT** on 15 nm AuNPs was determined to be approximately 4.6 molecules/nm^2^. This value is comparable to the reported surface densities of thiolate ligands on similarly sized AuNPs (5.7–6.2 molecules/nm^2^).^45^ The surface coverage was determined using ICP-MS and by monitoring the absorption spectra (λ_max_ = 400 nm) of *p*-nitrophenol released upon treatment with 1 M NaOH (Figure S3).

**Figure 2:**
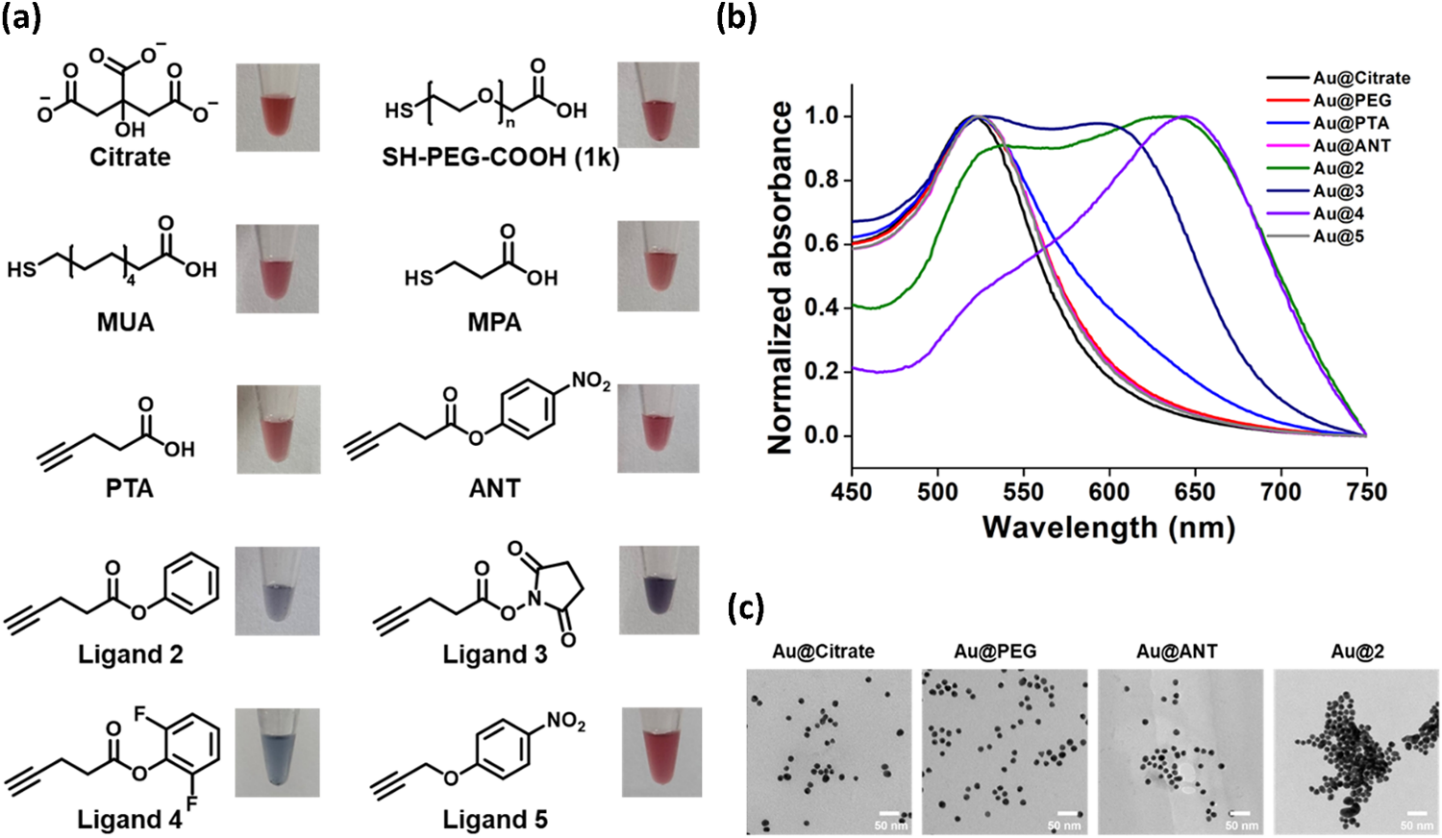
(a) Chemical structures of AuNP surface ligands and physical appearances of the corresponding ligand-capped AuNPs in aqueous solution. (b) UV–vis absorption spectra and (c) TEM images of the ligand-capped AuNPs.

**Table 1:** Maximum Absorption Wavelength (λ_abs_), ζ-Potential, and Hydrodynamic Diameter (*Z*-Average) of Ligand-Capped AuNPs

entry	sample	λ_abs_/nm	ζ-potential/mV	*Z*-average/nm
1	Au@Citrate	521	–31.0	37.7
2	Au@PEG	523	–13.1	135.0
3	Au@MUA	524	–34.0	56.0
4	Au@MPA	521	–25.0	53.8
5	Au@PTA	523	–28.8	35.7
6	Au@ANT	524	–20.4	38.3
7	Au@2	541, 634	–16.6	125.3
8	Au@3	528, 593	–25.1	124.4
9	Au@4	644	–19.7	77.2
10	Au@5	524	–19.3	39.0

To further assess the colloidal stability of **ANT**-functionalized AuNPs, we conducted a series of complementary analyses. Transmission electron microscopy (TEM) showed that the gold core size remained consistent at approximately 15 nm, typical for the AuNPs prepared by the Turkevich method (Figure 2c).^46^ Notably, **Au@ANT** displayed excellent dispersion, comparable to **Au@Citrate** and **Au@PEG**, with no signs of aggregation. The gold cores remain consistent at approximately 15 nm in diameter and exhibit no signs of aggregation after conjugation with streptavidin and Igg proteins (Figure S4). In contrast, **Au@2**, which bears a neutral phenyl ligand of similar size, exhibited significant clustering, indicating poor colloidal stability despite structural similarity to **ANT**. These observations were consistent with the results from UV–vis spectroscopy and visual appearance in solution shown in Figure 2a,2b. To further evaluate colloidal behavior, we performed dynamic light scattering (DLS) and zeta potential measurements (Table 1). AuNPs capped with nitro-containing ligands, including **ANT** and ligand **5**, exhibited hydrodynamic diameters (Z-average) of 38.3–39.0 nm, comparable to those of **Au@Citrate** (37.7 nm) and **Au@PTA** (35.7 nm). In contrast, AuNPs capped with ligands **2**, **3**, and **4** showed significantly larger hydrodynamic sizes (77.2–125.3 nm), despite their small molecular size (Figure S5). This size range approached that of **Au@PEG** (135 nm), which contains a much larger poly­(ethylene glycol) coating, suggesting that the size increase observed for ligands **2**–**4** is due to particle aggregation rather than ligand thickness. Zeta potential measurements further clarified these findings. **Au@Citrate** exhibited a highly negative surface charge (−31.0 mV), consistent with strong electrostatic stabilization and literature values for citrate-stabilized AuNPs.^47^ In comparison, **Au@ANT** (−20.4 mV), **Au@5** (−19.3 mV), **Au@2** (−16.6 mV), and **Au@4** (−19.7 mV) all exhibited moderately negative surface charges, indicating broadly similar surface potentials and overall neutral ligand character. Interestingly, despite their comparable zeta potentials, only **Au@ANT** and **Au@5** maintained excellent dispersibility in aqueous solution. This suggests that their enhanced colloidal stability is not solely governed by surface charge magnitude, but may instead stem from localized partial negative charges on the nitro group oxygens, which might promote electrostatic repulsion and suppress aggregation. These findings underscore the importance of subtle structural and electronic features in modulating interparticle interactions and colloidal stability.

### Streptavidin (SA) Conjugation with **Au@ANT**

Streptavidin (SA) is widely used in bioassays, proteomics, and imaging due to its strong and selective binding to biotin, which makes it an ideal protein for signal amplification and molecular targeting.^48–50^ Notably, SA lacks cysteine residues, making it a suitable model for evaluating the robustness of covalent conjugation methods that do not rely on thiol chemistry. To functionalize SA on AuNP surfaces using the **ANT** strategy, 1 μM SA was incubated with **Au@ANT** at 25 °C in pH 8.5 aqueous solution overnight to yield **Au@ANT@SA**. For comparison, SA was also conjugated to AuNPs using conventional physical adsorption (**Au@PA@SA**) and EDC/NHS-mediated chemical coupling to various carboxylated AuNPs under identical protein concentrations (Supporting Information, Material and Methods). To assess conjugation efficiency and nanoparticle stability, biotin binding assays, including lateral flow assay (LFA) and dot blotting, were employed. Rather than relying solely on absorption spectra and size measurements to infer conjugation success, these functional assays provide a more direct and meaningful evaluation of protein attachment and its retained bioactivity on the nanoparticle surface.

The conjugation efficiency of SA on AuNPs was first evaluated using lateral flow assay (LFA) with test strips containing biotinylated BSA immobilized on the test line (T). Each strip was dipped into Tris buffer containing the AuNP–SA conjugates for 10 min. Subsequently, images were captured using a smartphone camera and signal intensity was quantified using a gel scanner. As shown in Figure 3a, **Au@ANT@SA** produced a red test line approximately 29% more intense than that generated by **Au@PA@SA**, indicating significantly improved biotin-binding activity and conjugation efficiency (Figure S6). Dot blot analysis further confirmed that **Au@ANT@SA** produced stronger signals than **Au@PA@SA**. Notably, **Au@ANT@SA** also outperformed commercially available AuNP–SA conjugates, showing higher signal intensity in both dot blot and LFA test strips (Figure S7). In contrast, SA–conjugated AuNPs prepared via the EDC/NHS method displayed markedly reduced conjugation efficiency. Both LFA and dot blot results revealed faint signals and signs of aggregation for conjugates derived from MUA- and MPA-capped AuNPs.^20^ This aggregation was likely due to the rapid loss of electrostatic stabilization upon EDC activation of short carboxylate ligands (Figure S8). Additionally, **Au@PEG@SA** conjugates failed to generate detectable signals in either assay, which was attributed to the antifouling properties of PEG, hindering effective protein attachment by limiting adsorption between SA and the nanoparticle surface. Although shorter PEG chains can be used to improve conjugation efficiency, the nanoparticles exhibited aggregation after EDC/NHS treatment (Figure S9). Absorption spectra and DLS analyses showed that **Au@ANT@SA** and **Au@PA@SA** exhibited slight red shifts in their SPR bands after conjugation, consistent with modest increases in nanoparticle size (Figure S10 and Table S1). In contrast, **Au@PEG@SA** showed no appreciable spectral change following EDC/NHS treatment. These results are consistent with previous reports showing that EDC/NHS-mediated conjugation to carboxylated ligands often leads to poor coupling efficiency and nanoparticle aggregation.^19–25,51^ In particular, when PEG is used as a linker, this method typically does not produce obvious changes in the absorption spectra. To further quantify conjugation efficiency, the amount of unreacted SA remaining in the supernatant was analyzed using SDS-PAGE (Figure S11). The conjugation yield achieved with ANT (47%) was substantially higher than that obtained using physical adsorption (27%) or EDC/NHS peptide coupling with PEG capped ligand (≈0%), highlighting the superior functionality and stability of the ANT-mediated strategy for protein functionalization of AuNPs.

**Figure 3:**
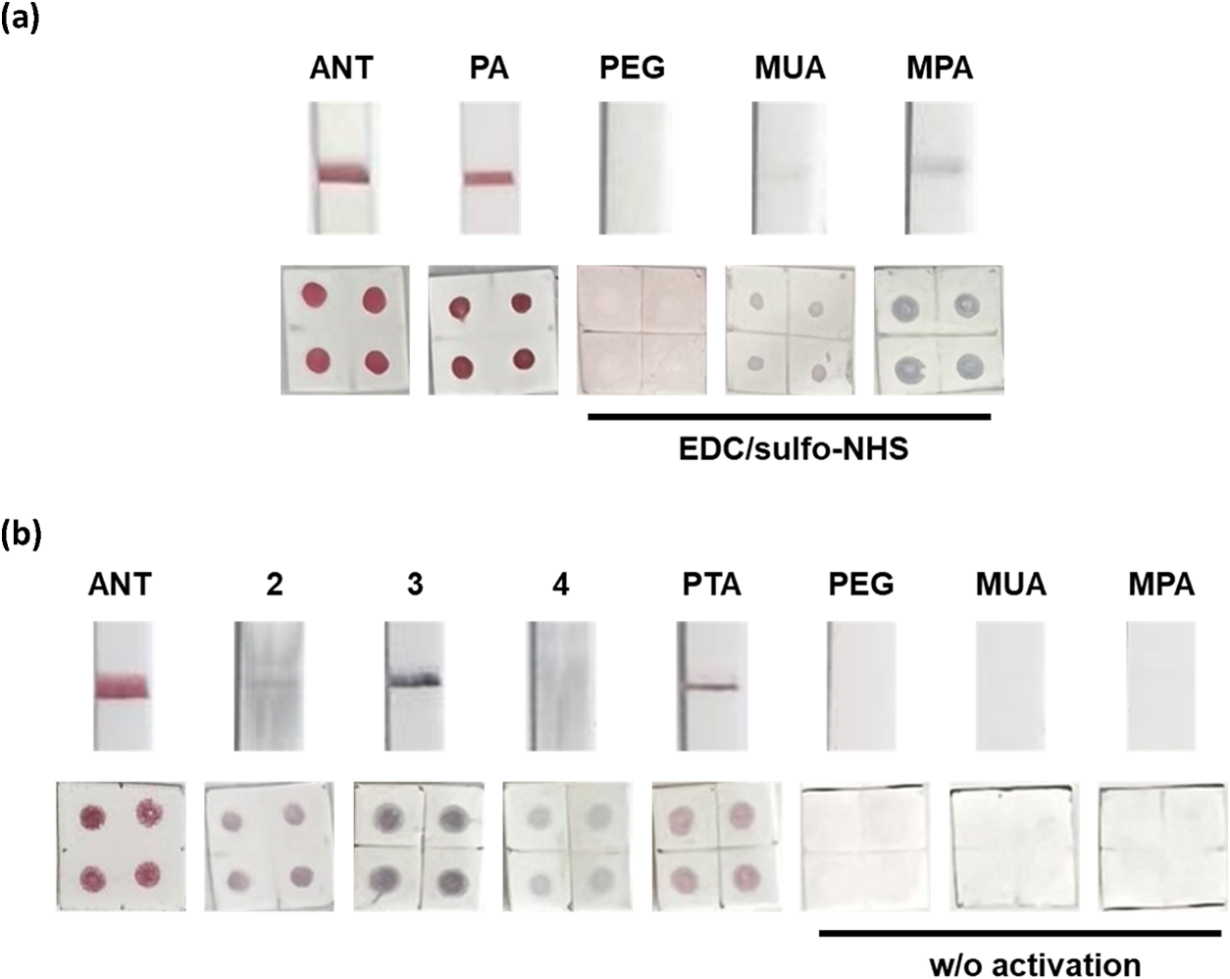
Representative LFA test strips and dot blot results of SA-AuNPs conjugates prepared using (a) ANT, PA, and conventional EDC/NHS coupling and (b) AuNPs capped with different surface ligands.

Next, we compared the efficiency of SA conjugation to AuNPs using various preactivated ligands, including **ANT**, phenyl ester (ligand **2**), NHS ester (ligand **3**), and difluorophenyl ester (ligand **4**), as well as carboxylated ligands without coupling reagents, such as **PTA**, **MUA**, and **MPA** (Figure 3b). Among these, only **Au@ANT@SA** produced a strong wine-red test line in both the LFA and dot blot assays. In contrast, conjugates prepared using ligands **2**–**4** generated weak or undetectable signals, often accompanied by blackened test lines or blot zones, suggesting poor conjugation efficiency and significant particle aggregation. It should be noted that LFA and dot blot assays may yield different outcomes. Large aggregates formed during conjugation may be unable to flow through the LFA strip, resulting in weak or absent test line signals, whereas the same aggregates can still be retained and visualized on dot blots. For AuNPs functionalized without EDC/NHS activation, the **PTA**-tethered particles gave weak but detectable signals, while **MUA**- and **MPA**-modified AuNPs showed no visible signal in either assay. This suggests that the shorter and more hydrophobic PTA ligand facilitates more effective protein adsorption than MUA and MPA, consistent with previous reports showing that shorter and hydrophobic ligands often enhance surface interaction and protein loading.^25^ To confirm that SA conjugation onto **Au@ANT** occurs via covalent linkage rather than adsorption, the nitrophenyl ester moiety in **ANT** was shown to react efficiently with protein nucleophiles (Figures S12 and S13). Furthermore, we investigated the effect of alkyl chain length on conjugation efficiency using **ANT** derivatives with varying carbon chain lengths (Figure S14). Increasing the chain length to 14 carbons significantly enhanced the ligand’s hydrophobicity, leading to partial aggregation of the gold nanoparticles and reduced both colloidal stability and conjugation efficiency. In contrast, shortening the chain to 3 carbons preserved nanoparticle dispersion but resulted in slightly lower signal intensity after SA conjugation compared to the original **ANT**. This reduction is likely due to a resonance effect that diminishes the reactivity of the nitrophenyl ester, thereby impairing protein conjugation efficiency.

Overall, the improved performance of **ANT** can be attributed to its preinstalled nitrophenyl ester moiety, which offers sufficient stability for handling while enabling effective covalent coupling to nucleophilic residues on the protein without the need for in situ activation. In contrast, ligands **2**–**4** failed to maintain colloidal stability, leading to poor conjugation efficiency and nanoparticle aggregation. These findings underscore the critical importance of ligand design, not only for enabling efficient bioconjugation but also for preserving nanoparticle dispersity throughout the protein modification process.

### Stability and Applications of **Au@ANT@SA**

Maintaining nanoparticle stability and preserving the activity of surface-conjugated proteins in complex environments are essential for the successful application of nanomaterials in biomedical settings. To assess this, **Au@ANT@SA** and **Au@PA@SA** were incubated in 60% fetal bovine serum (FBS) and 1 M NaCl, and their stability was evaluated using LFA test strips containing BSA-biotin at the test line. As shown in Figure 4a, **Au@ANT@SA** retained strong biotin-binding activity after 24 h of incubation at 37 °C, with minimal signal loss. In contrast, **Au@PA@SA** exhibited poor stability and a substantial reduction in signal intensity under the same conditions. Additionally, **Au@ANT@SA** demonstrated excellent storage stability in buffer containing 0.1% BSA, 0.1% tween 20, and 5% sucrose at 4 °C after 180 days, whereas **Au@PA@SA** showed a gradual decline in signal intensity upon storage (Figures 4b and S15). **Au@ANT@SA** also exhibited improved resistance to thiol-induced displacement compared to **Au@PA@SA**, as well as robust performance after lyophilization (Figures S16 and S17). These results highlight the exceptional physiological stability and protein retention afforded by **ANT**, supporting its broad potential for bioanalytical applications.

**Figure 4:**
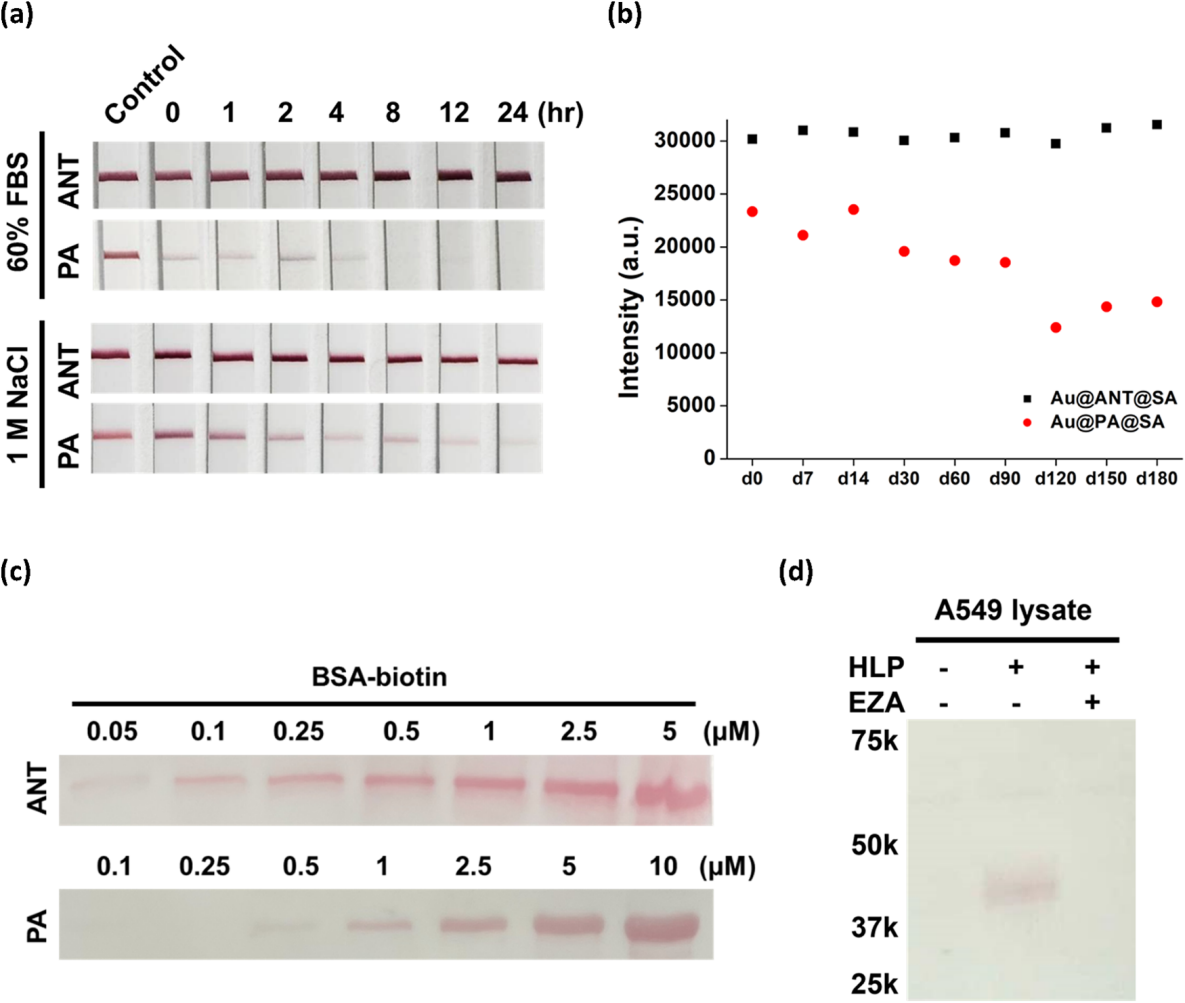
Stability and applications of **Au@ANT@SA**. (a) LFA test strip analysis of **Au@ANT@SA** and **Au@PA@SA** after incubation in 60% FBS or 1 M NaCl at 37 °C for 24 h. (b) Long-term storage stability of **Au@ANT@SA** and **Au@PA@SA** in conjugate buffer at 4 °C for 180 days and analyzed by LFA test strips. (c) Western blot detection of biotinylated-BSA at different concentrations and visualized with **Au@ANT@SA** and **Au@PA@SA**. (d) Western blot detection of endogenous hCAXII in A549 cells labeled with a biotinylated affinity probe (**HLP**) and visualized with **Au@ANT@SA**.

We next applied **Au@ANT@SA** in Western blotting. Currently, only a limited number of studies have utilized AuNP–protein conjugates as stains in blotting assays, and these often require silver enhancement or more sensitive detection techniques.^52–54^ Our results showed that **Au@ANT@SA** enables direct, one-step detection of biotinylated proteins on PVDF membranes without additional amplification (Figure 4c). Owing to the high conjugation efficiency and stability of the ANT system, **Au@ANT@SA** produced strong visible bands readily detectable by the naked eye and showed higher analytical sensitivity than **Au@PA@SA**, achieving a visual detection limit of at least 50 nM biotinylated protein. The superior detection sensitivity of **Au@ANT@SA** over **Au@PA@SA** for biotinylated protein detection was further confirmed by dot blot analysis, where visible signals were still observed even after a 400-fold dilution of **Au@ANT@SA** (Figure S18).

To demonstrate the utility of **Au@ANT@SA** in biologically relevant systems, it was applied to the detection of native human carbonic anhydrase XII (hCAXII), a transmembrane glycoprotein overexpressed in various cancers.^55–57^ As a hypoxia-associated biomarker, hCAXII contributes to tumor progression by acidifying the extracellular environment and promoting invasive behavior. A biotinylated affinity labeling probe (**HLP**) specific for hCA was used to selectively label cell surface hCAXII on A549 cancer cells, which are known to overexpress this hCA isoform (Figure S19).^58^ After labeling, the cell lysates were separated by SDS-PAGE and transferred to a PVDF membrane, where **Au@ANT@SA** was applied for one-step blotting of the biotinylated protein. As shown in Figure 4d, a distinct band was observed only in the presence of the hCA-targeting probe. No signal was detected when the probe was omitted or when the cells were pretreated with ethoxzolamide, an hCA inhibitor that blocks the probe binding. Together, these results demonstrate the effectiveness and reliability of **ANT** as a dual-function ligand for generating highly stable and functional protein–AuNP conjugates.

### Horseradish Peroxidase (HRP) Conjugation with **Au@ANT**

Horseradish peroxidase (HRP) is a widely used heme-containing metalloenzyme with broad applications in biomedical analysis, including chromogenic and fluorogenic signal amplification, enzyme-linked immunoassays (ELISA), biosensors, and immunohistochemical staining, owing to its oxidative catalytic activity in the presence of hydrogen peroxide (H_2_O_2_).^59^ Beyond diagnostics, HRP also plays a critical role in initiating polymerization reactions for hydrogel formation and material cross-linking.^60^ Despite having eight cysteine residues, HRP cannot participate in thiol–gold conjugation, as all cysteines are engaged in disulfide bonds essential for structural stability. While physical adsorption is commonly used to immobilize HRP on AuNPs, it often suffers from low enzyme retention.^61–63^ A recent strategy involving thiol-modified HRP has been reported to improve conjugation efficiency and stability, but it requires prior chemical modification of the enzyme, adding operational complexity to the enzyme modification on AuNPs.^64^

To directly compare the performance of different conjugation strategies, HRP–AuNP conjugates were prepared using **ANT**, physical adsorption (PA), and EDC/NHS-mediated coupling, yielding **Au@ANT@HRP**, **Au@PA@HRP**, **Au@PEG@HRP**, and **Au@MUA@HRP**, respectively. The preparation of **Au@PA@HRP** followed a literature-reported protocol, which demonstrated that horseradish peroxidase (HRP) retains its highest activity when conjugated under slightly acidic conditions (Figure S20).^64^ These conjugates were then evaluated using a tetramethylbenzidine (TMB) chromogenic assay to assess their enzymatic activity and stability (Figure 5a). Absorbance at 450 nm was recorded for each conjugate following TMB/H_2_O_2_ treatment, revealing that **Au@ANT@HRP** retained about 56% of the catalytic activity of native HRP, whereas **Au@PA@HRP**, **Au@PEG@HRP**, and **Au@MUA@HRP** exhibited only 28%, 1%, and 10% activity, respectively (Figure 5b). To gain insight into the enzymatic performance, the maximum velocity (*V*_max_) and Michaelis constant (*K*_m_) of the native and the HRP–AuNP conjugates were assessed (Figure 5c and Table S2). The *V*_max_ of **Au@ANT@HRP** was determined to be 10.8 μM/min, nearly twice that of **Au@PA@HRP** (4.8 μM/min). Another indicator of catalytic performance, *K*_cat_/*K*_m_, also showed a significantly higher value for **Au@ANT@HRP** (38 min^–1^ μM^–1^) than that of **Au@PA@HRP** (22 min^–1^ μM^–1^), confirming **ANT** as an excellent way to fabricate **Au@HRP**. The stability and reusability of the conjugates were then assessed through three consecutive cycles of centrifugation, washing, and reuse in the TMB assay (Figure 5d). **Au@ANT@HRP** retained 96% of its initial activity after three cycles, demonstrating exceptional stability during repeated handling. In contrast, **Au@PA@HRP** exhibited a substantial decrease in activity, retaining only ≈41% after the same number of cycles. Beyond reusability, **Au@ANT@HRP** also exhibited pronounced resistance to proteolytic degradation (Figure 5e). After 16 h of trypsin digestion at 37 °C, **Au@ANT@HRP** preserved approximately 76% of its initial activity, whereas native HRP and **Au@PA@HRP** retained only about 28% and 32% activity, respectively.

**Figure 5:**
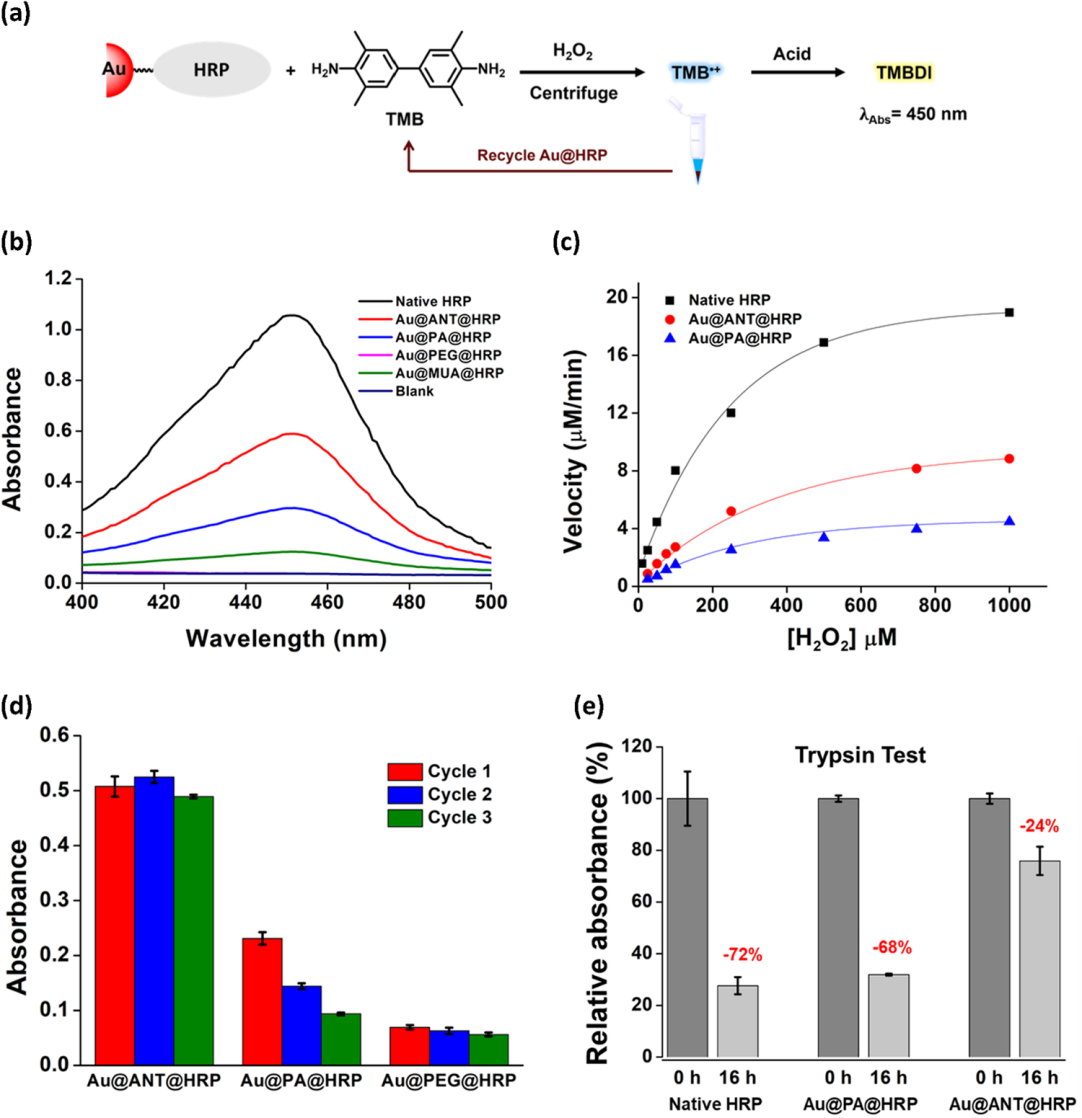
Horseradish Peroxidase conjugation with **Au@ANT**. (a) Schematic illustration of the HRP-catalyzed oxidation of TMB by H_2_O_2_ and recycling of **Au@HRP** conjugates. (b) Absorption spectra of the oxidation product (TMBDI) catalyzed by native HRP, **Au@ANT@HRP**, **Au@PA@HRP**, **Au@PEG@HRP**, and **Au@MUA@HRP**. (c) Michaelis–Menten plots comparing the catalytic activity of native HRP, **Au@ANT@HRP**, and **Au@PA@HRP** using varying H_2_O_2_ concentration. (d) Reusability of **Au@ANT@HRP**, **Au@PA@HRP** and **Au@PEG@HRP** analyzed by absorbance of TMBDI at 450 nm after three reaction cycles. (e) Resistance of native HRP, **Au@ANT@HRP**, and **Au@PA@HRP** to proteolytic degradation after 16 h of trypsin digestion at 37 °C.

Overall, these results demonstrate that **ANT**-mediated conjugation produces HRP–AuNP conjugates with markedly higher catalytic activity, kinetic efficiency, and stability than those prepared by PA or EDC/NHS coupling. The improved performance is attributed to **ANT**’s stable covalent linkage, which prevents enzyme desorption, minimize structural distortion, and maintain active-site accessibility, making it a robust and generalizable strategy for enzyme immobilization on gold nanoparticles.

### Immunoglobulin G (IgG) Conjugation with **Au@ANT**

Antibodies, particularly immunoglobulin G (IgG), are key immune proteins widely conjugated to AuNPs for applications ranging from immunoassays and biosensors to targeted cancer therapy and advanced imaging techniques.^4,5,65^ In most cases, IgG is immobilized on AuNP surfaces via physical adsorption (PA), mediated by electrostatic interactions or the presence of accessible cysteine residues. This simple, coupling-agent-free approach has enabled the commercialization of numerous IgG–AuNP conjugates for point-of-care testing. However, an inherent limitation of PA is the need for high IgG concentrations to fully passivate the AuNP surface during conjugation, which is an especially costly requirement when working with expensive or scarce antibodies. Indeed, numerous studies have reported that AuNP–IgG conjugates prepared with insufficient antibody coverage or under neutral pH conditions are prone to aggregation.^5–8,65^ To address these challenges, the **ANT**-mediated covalent conjugation strategy was employed, enabling stable IgG immobilization at lower antibody concentrations and neutral pH while maintaining colloidal stability and functional activity.

To assess **ANT**-mediated antibody conjugation, 100 nM mouse IgG was coupled to AuNPs using various strategies, including **ANT**, PA, EDC/NHS-mediated coupling, and ligands such as **2**, **3**, **4**, **PTA**, **MUA**, and **MPA**. Conjugation efficiency and performance were evaluated using lateral flow assays (LFA), in which goat antimouse IgG was immobilized on the test line as the capture target. Among all conditions tested, **Au@ANT@IgG** exhibited the highest colloidal stability and produced the strongest test line signal on the LFA strips (Figures 6a and S21). In contrast, IgG–AuNP conjugates prepared using EDC/NHS coupling or ligands **2**, **3**, **4**, and **PTA** showed weaker signal intensities and visible signs of aggregation during conjugation. In line with experimental results, the IR spectra showed no significant changes after EDC/NHS activation or IgG treatment (Figure S22). We also investigated the relative IgG loading on AuNPs using SDS-PAGE analysis of the unreacted IgG remaining in the supernatant after conjugation. The results showed that ANT provides the highest protein-conjugation efficiency (Figure S23). It is noteworthy to mention that the absorbance of **Au@ANT@IgG** was typically slightly lower than that of the corresponding **Au@PA@IgG** samples (Figure S24). While both **Au@ANT@IgG** and **Au@PA@IgG** demonstrated remarkable stability in FBS and 1 M NaCl conditions, **Au@ANT@IgG** showed greater resistance to thiolated compounds such as dithiothreitol (DTT) (Figure S25). These findings are consistent with results obtained for SA- and HRP-functionalized AuNPs, further confirming the improvement offered by the **ANT** ligand for stable and efficient protein attachment.

**Figure 6:**
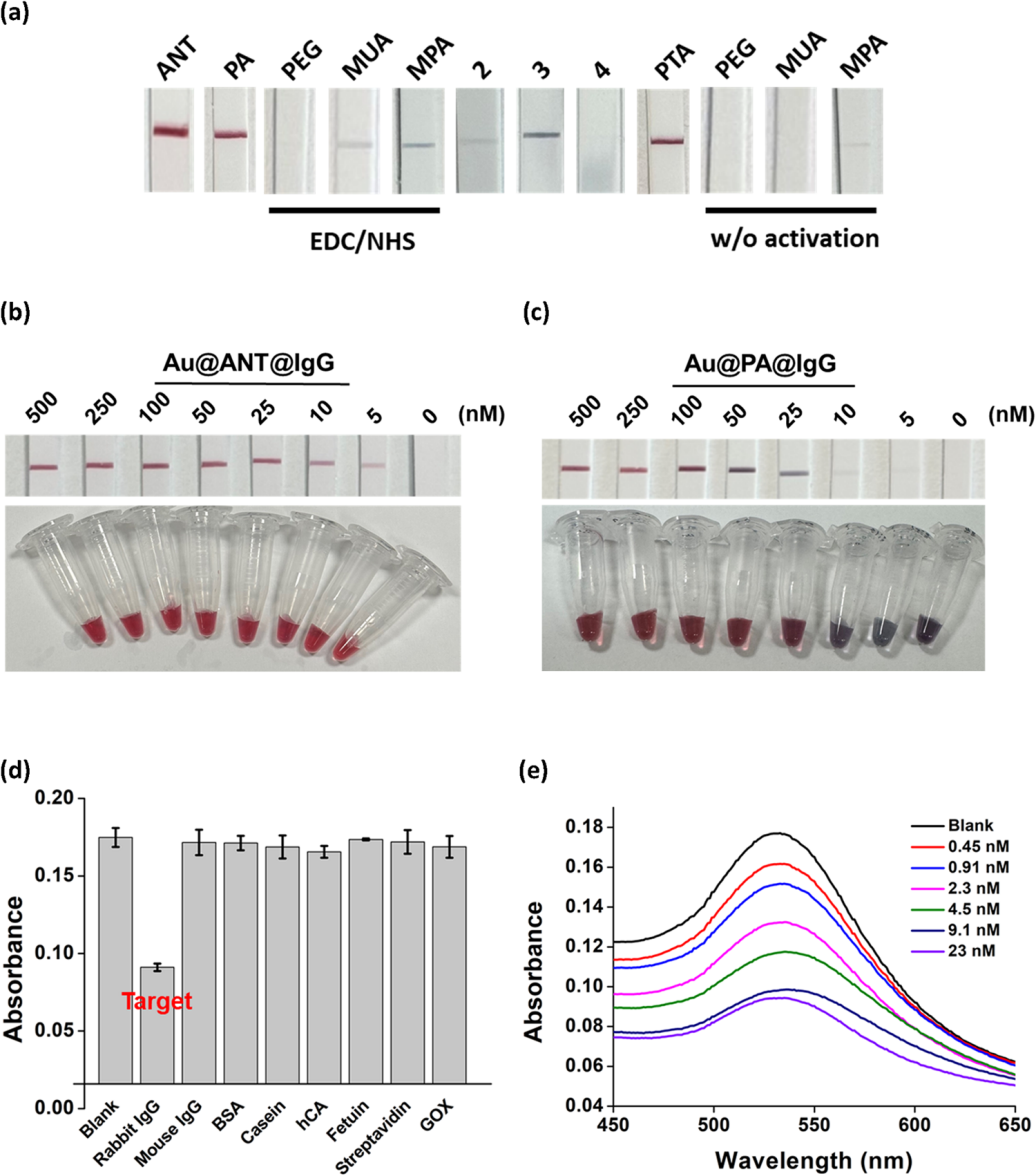
Conjugation and application of IgG with **Au@ANT**. (a) Representative LFA test strips results of IgG conjugated AuNPs prepared using **ANT**, PA, EDC/NHS coupling and AuNPs capped with different surface ligands. (b) LFA test strips and visual appearance of **Au@ANT@IgG** prepared with varying IgG concentrations, showing strong test line signals even at reduced antibody loadings. (c) LFA test strips of **Au@PA@IgG** prepared with varying IgG concentrations, showing aggregation and signal loss at low antibody loadings. (d) Absorbance of **Au@ANT@IgG** after incubation with 9.1 nM rabbit IgG (target) or 91 nM of nontarget proteins. (e) Absorption spectra of **Au@ANT@IgG** after treatment with different concentrations of rabbit IgG at 37 °C. The detailed protocol for the precipitation assay is provided in the Supporting Information.

To further examine the effect of surface coverage on conjugate stability, AuNPs were functionalized with varying concentrations of IgG using the **ANT** and PA approach. LFA, absorption spectroscopy, and visual inspection demonstrated that **Au@ANT@IgG** maintained excellent colloidal stability and strong signal intensity across all tested IgG concentrations, despite being prepared in a neutral pH 7 aqueous solution, a condition that typically promotes aggregation via the PA method, as reported in the literature (Figure 6b).^7,8,66^ Remarkably, even at a low antibody loading of 5 nM, the conjugates retained their characteristic wine-red color and showed no spectral broadening. In contrast, **Au@PA@IgG** exhibited progressive aggregation and signal loss as the IgG concentration decreased, with absorption spectra broadening already apparent at 100 nM and worsening at lower loadings (Figures 6c and S26). These observations are in agreement with prior reports showing that IgG–AuNP conjugates prepared at low protein concentrations are prone to aggregation. The ability of **Au@ANT@IgG** to remain stable and functional under low antibody loading conditions highlights its practical value in minimizing IgG consumption, which is an important advantage for cost-sensitive applications involving expensive or scarce antibodies.

Immunoprecipitation is one of the most widely used methods for antigen detection, relying on the principle that antibodies can bind multivalent antigens to form cross-linked aggregates that precipitate out of solution. Several IgG–AuNP conjugates have been developed for colorimetric or turbidimetric antigen detection based on this aggregation-induced precipitation principle to improve detection sensitivity.^67–70^ However, these approaches often require large amounts of antibody to prevent nonspecific aggregation or suffer from low sensitivity. Using **Au@ANT@IgG** prepared with only 25 nM goat antirabbit IgG, selective colorimetric detection of 9.1 nM rabbit IgG (target) was achieved even in the presence of a 10-fold excess of nontarget proteins (Figures 6d and S27). The conjugates displayed a limit of detection (LOD) of approximately 0.85 nM, substantially lower than that reported for conventional protein precipitation assays (1.7–133 nM) (Figures 6e and S28).^67–70^ In contrast, **Au@PA@IgG** prepared using 25 nM IgG could not be used for the precipitation assay, as it readily aggregated at low antibody loadings (Figure S29). These results highlight the potential of **Au@ANT@IgG** to deliver high-sensitivity immunoprecipitation-based assays while minimizing antibody consumption.

## Conclusions

We have introduced Alkyne Nitro Tag (**ANT**) as a compact heterobifunctional ligand that uniquely stabilizes gold nanoparticles through the electrostatic repulsive effects of its nitro substituent while simultaneously enabling direct covalent protein conjugation via a preinstalled nitrophenyl ester. This design circumvents the limitations of conventional strategies, such as the instability of physical adsorption and the inefficiency of EDC/NHS coupling, by offering a robust and generalizable platform for protein functionalization. Through systematic comparisons with SA, HRP, and IgG, **ANT** was shown to yield conjugates with improved stability, activity retention, and reusability, even under harsh biological and chemical conditions. Moreover, **Au@ANT** protein conjugates supported diverse applications, including lateral flow assays, Western blotting, enzymatic sensing, and immunoprecipitation, achieving higher sensitivity and markedly reduced protein consumption. Taken together, these findings establish **ANT** as a fundamentally new strategy for engineering protein–nanoparticle conjugates, opening avenues for advanced nanomaterial design and bioanalytical technologies.

## Experimental Section

### Materials and Instruments

All chemicals and reagents were purchased from Sigma-Aldrich or TCI and used without further purification. Solvents including DMSO, DMF, DCM, acetonitrile, hexane, ethyl acetate, and methanol were obtained from Sigma-Aldrich or TCI and used as received. Flash column chromatography was performed using silica gel (230–400 mesh, Merck). Analytical HPLC was carried out on an XBridge BEH C18 column (130 Å, 5 μm, 4.6 × 250 mm^2^). Products were purified using a semipreparative HPLC column (Cosmosil 20 mm ID × 150 mm, 5C18-AR-300, Nacalai Tesque). Antibodies were purchased from Jackson ImmunoResearch. Streptavidin was obtained from Fitzgerald, and horseradish peroxidase (HRP) from Sigma-Aldrich. Lateral flow assay (LFA) membranes (Whatman FF120HP) were purchased from Cytiva, backing cards from Prisma Biotech, absorbent pads from Merck, and sample pads from Advanced Microdevices.

^1^H and ^13^C NMR spectra were recorded on Varian UnityInova-500, Varian MR-400, or Bruker-400 spectrometers. Chemical shifts (δ) are reported in ppm relative to residual solvent signals: δ 7.24 (CDCl_3_) and δ 3.31 (CD_3_OD) for 1H; δ 77.0 (CDCl_3_) and δ 49.0 (CD_3_OD) for ^13^C. High-resolution mass spectra (HRMS) with electrospray ionization (ESI) were measured on a JEOL JMS-T100LP 4G. UV–vis absorption spectra were recorded using a TECAN Infinite M200 PRO microplate reader with Thermo Scientific 96-well microplates. Images were captured on a ChemiDoc Touch Imaging System (Bio-Rad, CA, USA) and analyzed using Image Lab software (Bio-Rad, CA, USA). TEM images were acquired with a Hitachi HT7700. Dynamic light scattering (DLS) and ζ-potential measurements were performed using a Malvern Zetasizer Nano. All LFA strip and dot blot images presented in this work were captured using a smartphone camera.

### Synthesis of Citrate-Capped AuNPs (**Au@citrate**)

Spherical AuNPs (≈15 nm) were synthesized using the sodium citrate reduction method (Turkevich method). An aqueous solution of HAuCl_4_ (1 mM, 50 mL) was heated to reflux, followed by the rapid addition of trisodium citrate solution (38.8 mM, 6 mL). The mixture was maintained under reflux for 7 min, after which the resulting AuNP solution was cooled to room temperature and stored at 4 °C until further use.

### Surface Modification of AuNPs with Ligands

**Au@citrate** (200 μL, OD_520_ = 1.0) was diluted with deionized water (790 μL). **ANT** or other ligands (10 mM, 10 μL) were then added, and the mixture was shaken at 25 °C for 4 h (Schemes S1 and S2). The resulting solution was centrifuged at 10,000 rpm for 30 min, and the supernatant was discarded. The pellet was resuspended in deionized water (990 μL) and centrifuged again under the same conditions. After removing the supernatant, the purified ligand-modified AuNPs were collected for further experiments.

### Determination of Surface Coverage of **ANT** on AuNPs

200 μL of 6.6 nM AuNP was reacted with **ANT**. After centrifugation, the resulting **Au@ANT** was resuspended to 50 μL. The resulting **Au@ANT** solution was mixed with an equal volume of 1 M NaOH for 2 h to completely hydrolyze the nitrophenyl ester. After centrifugation at 10,000 rpm for 30 min, the supernatant was collected and analyzed by UV–vis absorption. The concentration of released p-nitrophenol was determined to be 4.4 nmol by comparison with calibration curves prepared from aqueous standards of known concentrations. The concentration of gold in the initial **Au@citrate** solution (200 μL) was determined by inductively coupled plasma mass spectrometry (ICP-MS) to be 142 ppm. Thus, the concentration of 15.2 nm AuNPs was calculated as 6.6 nM and 0.00132 nmol of AuNPs were present in the ligand modification reaction. Based on these values, the number of **ANT** molecules per AuNP was estimated to be approximately 3333 and the surface coverage of **ANT** was calculated to be 4.6 molecules per nm^2^.

### AuNP–Protein Conjugation through **ANT**

**Au@ANT** solution was diluted with deionized water (pH 7) or borate buffer (pH 8.5) to a final volume of 198 μL. Protein solution (2 μL) was added to achieve final concentrations of 1 μM streptavidin (SA), 50 nM horseradish peroxidase (HRP), or 0–500 nM mouse IgG. The mixtures were shaken at room temperature overnight, after which the AuNP conjugates were blocked with 150 μM BSA for 1 h. The suspensions were centrifuged at 10,000 rpm for 30 min and the supernatant was discarded. The pellets were washed once with 200 μL deionized water, followed by a second centrifugation at 10,000 rpm for 30 min. The resulting conjugates were resuspended and stored in Tris buffer (for **Au@ANT@HRP**) or conjugate buffer (5 mM sodium borate, 0.1% BSA, 0.1% Tween-20, and 5% D-sucrose) (for **Au@ANT@SA** and **Au@ANT@IgG**) to a final volume of 100 μL. For the antibody precipitation assay, **Au@ANT@IgG** was prepared by incubating 25 nM goat antirabbit IgG for 6 h, followed by blocking with 100 nM BSA for 12 h. After centrifugation and washing as described above, the conjugates were stored in PBS buffer until use.

### AuNP–Protein Conjugation through EDC/NHS Peptide Coupling

**Au@citrate** (200 μL, OD_520_ = 1.0) was centrifuged at 5000 g for 30 min. The supernatant was carefully removed, and the pellet was resuspended in 200 μL deionized water. To this suspension, 2 μL of SH-PEG­(1k)-COOH, MUA, or MPA (10 mM) was added, and the mixture was shaken at 25 °C for 1 h. The reaction mixture was centrifuged at 10,000 rpm for 30 min and the supernatant was discarded. The pellet washed once with deionized water. After a second centrifugation under the same conditions, the pellet was resuspended in 200 μL deionized water for subsequent protein coupling. For the one-pot protocol, EDC (100 mM, 2 μL) was added to the AuNP solution, followed immediately by sulfo-NHS (100 mM, 2 μL). After 10 min, 2 μL of protein was introduced to achieve final concentrations of 1 μM SA, 50 nM HRP or 100 nM IgG. The mixture was shaken overnight at room temperature, then blocked with 150 μM BSA for 1 h. The suspension was centrifuged at 10,000 rpm for 30 min and the supernatant discarded. The pellet washed once with 200 μL deionized water. After a second centrifugation under the same conditions, the pellet was resuspended in either Tris buffer or conjugate buffer for storage. For the two-step protocol, EDC (100 mM, 2 μL) was added to the AuNP suspension, followed by sulfo-NHS (100 mM, 2 μL). After 1 h, the mixture was centrifuged at 10,000 rpm for 30 min to remove excess coupling agents, and the pellet was resuspended in 200 μL deionized water. Protein (2 μL, final concentration 1 μM SA, 50 nM HRP or 100 nM IgG) was then added, and subsequent steps were carried out as described in the one-pot protocol.

### AuNP–Protein Conjugation through Physical Adsorption

The pH of **Au@citrate** (200 μL, OD_520_ = 1.0) was adjusted using 0.2 M potassium carbonate to pH 8.0 (for SA), pH 6.5 (for HRP), or pH 8.5 (for IgG). Protein solution (2 μL) was added to achieve final concentrations of 1 μM SA, 50 nM HRP, or 0–500 nM mouse IgG. The mixtures were shaken at room temperature for 1 h, after which the AuNP conjugates were blocked with 150 μM BSA for an additional 1 h. The suspensions were centrifuged at 12,000 rpm for 15 min and the supernatant discarded. The pellets were washed once with 200 μL deionized water. After a second centrifugation at 12,000 rpm for 15 min, the supernatant was removed, and the pellets were resuspended in Tris buffer or conjugate buffer to a final volume of 100 μL for storage.

### Determination of Unreacted Streptavidin and Conjugation Yield by SDS-PAGE

Supernatants from AuNP–SA conjugation reactions performed via ANT, PA, and EDC/NHS methods (without BSA blocking) were collected. Each supernatant was mixed with 4× loading buffer (250 mM Tris-HCl, 40% glycerol, 8% SDS, 20% DTT) and heated at 90 °C for 10 min. Samples were then loaded onto a 10% SDS–PAGE gel, and electrophoresis was carried out at 95 V and 50 A. Following electrophoresis, gels were stained with Coomassie Brilliant Blue (CBB). Images were captured using both a smartphone camera and the ChemiDoc Touch Imaging System (Bio-Rad). To determine the conjugation yield, SDS–PAGE was also performed on SA standards of known concentrations to generate a calibration curve. Band intensities were quantified using Image Lab software, and the concentration of unreacted SA in each supernatant was calculated. Conjugation yields were then determined by comparing the amount of unreacted SA to the initial protein input.

### TEM Imaging of AuNP Conjugates

AuNP samples (10 μL) were deposited onto TEM support grids (TED PELLA INC., 01800-F, F/C, 200 mesh). After 7 min, excess liquid was removed using lens wipes, and the grids were dried in a chemical fume hood overnight before imaging.

### DLS and ζ-Potential Analysis of AuNP Conjugates

For dynamic light scattering (DLS) measurements, **Au@ligand** samples were diluted with deionized water to a final volume of 1.5 mL and loaded into disposable cuvettes. Measurements were performed with 15 scans of 10 s each. For ζ-potential analysis, **Au@ligand** samples were diluted to a final volume of 0.75 mL, loaded into DTS-1070 sample cells, and analyzed with 20 measurements per run over three runs.

### LFA Tests of **Au@SA**

BSA–biotin (20 μM) and goat antimouse IgG (0.25 mg/mL) were used to coat the test and control lines, respectively. The running solution contained Tris buffer (50 mM), BSA (250 μM), SDS (1%), control particles (5 μL), and test particles (5 μL) in a total volume of 50 μL. Control particles were AuNPs functionalized with mouse IgG (300 nM) by physical adsorption. Images were captured using a smartphone camera, while signal intensities were recorded and quantified using the ChemiDoc Touch Imaging System and Image Lab software (Bio-Rad). Test line intensity was used as the primary measure of conjugate stability and performance. For complex media stability tests, FBS or NaCl (2 M) in Tris buffer was added to the running solution (containing **Au@SA**) to achieve final concentrations of 60% FBS or 1 M NaCl. The mixtures were incubated at 37 °C for 0, 1, 2, 4, 8, 16, and 24 h prior to testing. For thiol interference tests, **Au@SA** conjugates were incubated with GSH (5 mM) or DTT (5 mM) in running buffer at 37 °C for 1 h, after which test strips (BSA–biotin-coated test line) were immersed in the mixture for 30 min. For long-term storage tests, **Au@SA** conjugates were stored at 4 °C and evaluated on days 0, 7, 14, 30, 60, 90, 120, 150, and 180. For lyophilization tests, conjugates were lyophilized to remove solvent and reconstituted with 100 μL H_2_O. Both lyophilized and nonlyophilized conjugates were mixed with 2.5 μL of 5 mM BSA and 1% SDS in 50 mM Tris buffer (final volume: 50 μL), and test strips were immersed in the mixtures for 30 min.

### Dot Blotting of **Au@SA**

Nitrocellulose membranes were cut into 1 cm × 1 cm squares and divided into four sections. Test dots were coated with BSA–biotin, and the membranes were incubated at 37 °C for 30 min to remove residual moisture. The membranes were then blocked with 5% milk in PBS for 1 h, followed by washing with PBS. For testing, **Au@SA** (50 μL) was diluted with 50 mM Tris buffer to a final volume of 1 mL, and the membranes were immersed in the solution overnight. After incubation, the membranes were washed with PBST (1% Triton X-100 in PBS) to remove unbound AuNPs. Images were captured with a smartphone camera, and signal intensities were recorded using the ChemiDoc Touch Imaging System (Bio-Rad) and quantified with Image Lab software (Bio-Rad).

### Western Blotting of **Au@SA**

Protein samples were separated by SDS–PAGE (95 V, 50 A, 100 min) and transferred to a methanol-activated PVDF membrane (90 V, 150 A, 90 min). The membrane was washed with PBST (0.1% Tween-20 in PBS) and blocked with 5% milk in PBST. It was then incubated overnight at 100 rpm in a mixture of **Au@SA** (40 μL, 3× concentrated) and PBST (8 mL). After incubation, the membrane was washed three times with PBST. Images were captured using a smartphone camera, and the signals were recorded with the ChemiDoc Touch Imaging System. hCA labeling was performed following a previously reported protocol (ACS Sens. **2022**, 7, 2691–2700).

### Chromogenic Reaction of TMB

A TMB stock solution was prepared by dissolving TMB in DMSO at 10 mg/mL. H_2_O_2_ solution was prepared by diluting 30% H_2_O_2_ with citrate–PBS buffer (25 mM, pH 5) to 25 ppm. For the reaction, 400 μL of H_2_O_2_ solution, 390 μL of deionized water, and 5 μL of TMB stock solution were premixed. An aliquot of 100 μL of this mixture was then added to microcentrifuge tubes containing 5 μL of native HRP or **Au@HRP**. After incubation at 37 °C for 15 min, the reaction was quenched by addition of 100 μL of 3 M H_2_SO_4_. The oxidized TMB product (TMBDI) was analyzed using a UV–vis absorption spectrometer (TECAN Infinite M200 PRO).

### Enzyme Kinetics of Native HRP and **Au@HRP**

For kinetic assays, 1.8 μL of 41.6 mM TMB (10 mg/mL in DMSO) and 6 μL of native HRP or **Au@HRP** were premixed with 277.2 μL of deionized water. H_2_O_2_ (15 μL) at various concentrations (0.5, 1.0, 1.5, 2.0, 5.0, 15, and 20 mM) was then added to reach a final volume of 300 μL. At 30 s intervals, 50 μL of the reaction mixture was withdrawn, quenched with an equal volume of 3 M H_2_SO_4_, and analyzed by UV–vis absorption at 450 nm. Kinetic parameters were determined by fitting initial rates (*V*_0_) to the Michaelis–Menten equation.

### Recyclability of **Au@HRP**

For recycling tests, 400 μL of H_2_O_2_ solution, 395 μL of deionized water, and 5 μL of TMB stock solution were premixed. Aliquots of 100 μL were added to microcentrifuge tubes containing 20 μL of **Au@HRP** or Tris buffer (blank control). After incubation at 37 °C for 5 min, the mixtures were centrifuged at 7400 g for 15 min. From the supernatant, 50 μL was withdrawn, quenched with an equal volume of 3 M H_2_SO_4_, and analyzed by UV–vis absorption at 450 nm. The remaining supernatant was discarded, and the pellet was washed with 200 μL of Tris buffer (25 mM), followed by centrifugation at 7400 g for 30 min. The supernatant was removed, and the pellet was resuspended in 20 μL of Tris buffer (25 mM) for the next cycle.

### Trypsin Digestion Assay

**Au@HRP** (2 μL) was mixed with deionized water (38 μL) and trypsin solution (10 μL, 0.25% w/v, Corning, USA) in microcentrifuge tubes and incubated at 37 °C for 16 h. Meanwhile, H_2_O_2_ solution (400 μL, 25 ppm) was premixed with TMB stock solution (5 μL, 10 mg/mL in DMSO). To initiate the reaction, 50 μL of this mixture was added to the trypsin-treated samples. After incubation at 37 °C for 5 min, the reactions were quenched with 100 μL of H_2_SO_4_ (3 M), and the oxidized TMB product (TMBDI) was analyzed using a UV–vis absorption spectrometer. For each group, untreated HRP (0 h trypsin) served as a control.

### LFA Tests of **Au@IgG**

Goat antimouse IgG (0.25 mg/mL) was used to coat the test line. The running buffer contained Tris (50 mM), BSA (250 μM), Tween-20 (1%), and test particles (5 μL) in a total volume of 50 μL. Test strips were immersed in the mixtures for 15 min. Images were captured with a smartphone camera, and signal intensities were recorded using the ChemiDoc Touch Imaging System and quantified with Image Lab software (Bio-Rad). For stability in complex media, FBS or NaCl (2 M) was added to the running buffer (containing **Au@IgG**) to final concentrations of 60% FBS or 1 M NaCl. Mixtures were incubated at 37 °C for 0, 24, 48, and 72 h before testing. For thiol interference tests, **Au@IgG** conjugates were incubated with 0.5 mM GSH, cysteine (Cys), DTT, or ethanethiol in running buffer at 37 °C for 1 h. Test strips were immersed in the treated mixtures for 15 min.

### Precipitation Assay

**Au@IgG** (20 μL) was mixed with sample protein (2 μL) and incubated at 37 °C for 1 h, followed by addition of PBS buffer (80 μL). After another 1 h incubation at 37 °C, 90 μL of the reaction solution was carefully withdrawn and analyzed by UV–vis absorption spectroscopy. For the selectivity tests, the target protein (rabbit IgG) concentration was 9.1 nM, while nontarget proteins were used at 91 nM. The limit of detection (LOD) was calculated as LOD = 3.3σ/*S*, where σ is the standard deviation of blank measurements and *S* is the slope of the calibration curve.

## Supplementary Material



## Notes

Probe synthesis, gold nanoparticle preparation, absorption spectra, LFA test strip and dot blot images, quantitative analysis, and NMR and HRMS spectra (PDF)
