# Supplementary material for: Alkyne Nitro Tag Enables Stable and Efficient Protein Functionalization of Gold Nanoparticles
Source: ACS Appl Mater Interfaces. 2026 Mar 4;18(10):15867–80. doi: 10.1021/acsami.6c01215 (PMC13006950; doi:10.1021/acsami.6c01215)
Supplement: Supplementary file 1 [file am6c01215_si_001.pdf]

## Supporting Information

# Alkyne Nitro Tag Enables Stable and Efficient Protein Functionalization of Gold Nanoparticles

**Shun-Qiang Xu,[†] Yung-Kun Pan,[†] Po-Cheng Lin,[†] Ling-Ling Weng,[†] Tzu-Jung Chang,[†] Chien-Chi Wu,[†] Yun-Rong Peng[†] and Kui-Thong Tan[†,‡,*]**

[†] Department of Chemistry, National Tsing Hua University, 101 Section 2, Kuang Fu Road, Hsinchu 30013, Taiwan, Republic of China

[‡] Department of Medicinal and Applied Chemistry, Kaohsiung Medical University, Kaohsiung 80708, Taiwan, Republic of China

Corresponding Author: kttan@mx.nthu.edu.tw

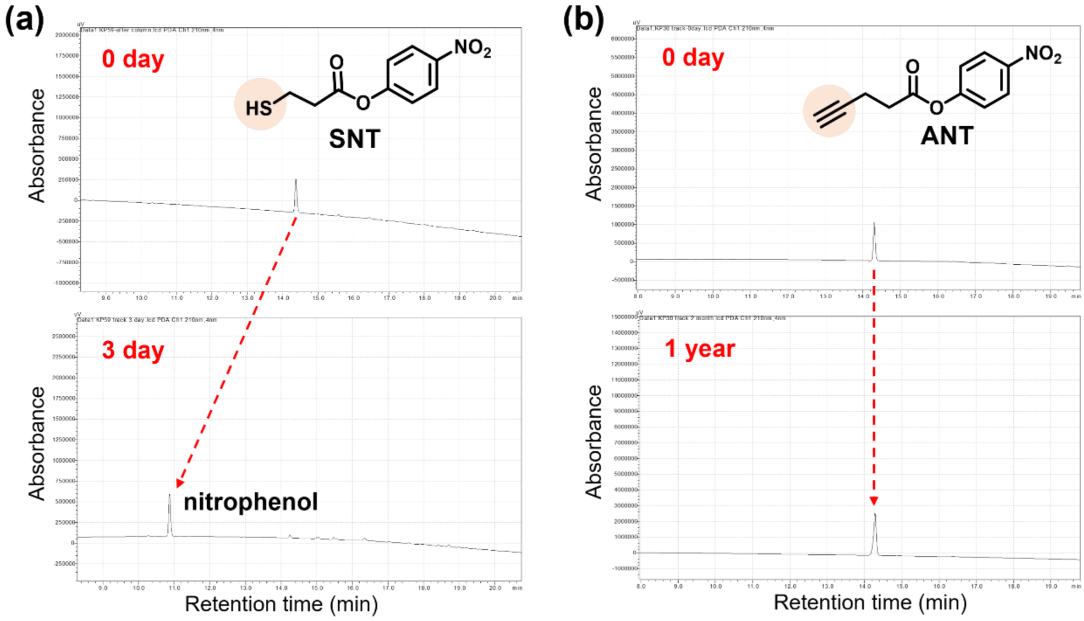


**Figure S1.** Time-course HPLC traces of (a) **SNT** and (b) **ANT** stored in DMSO at –80 °C.

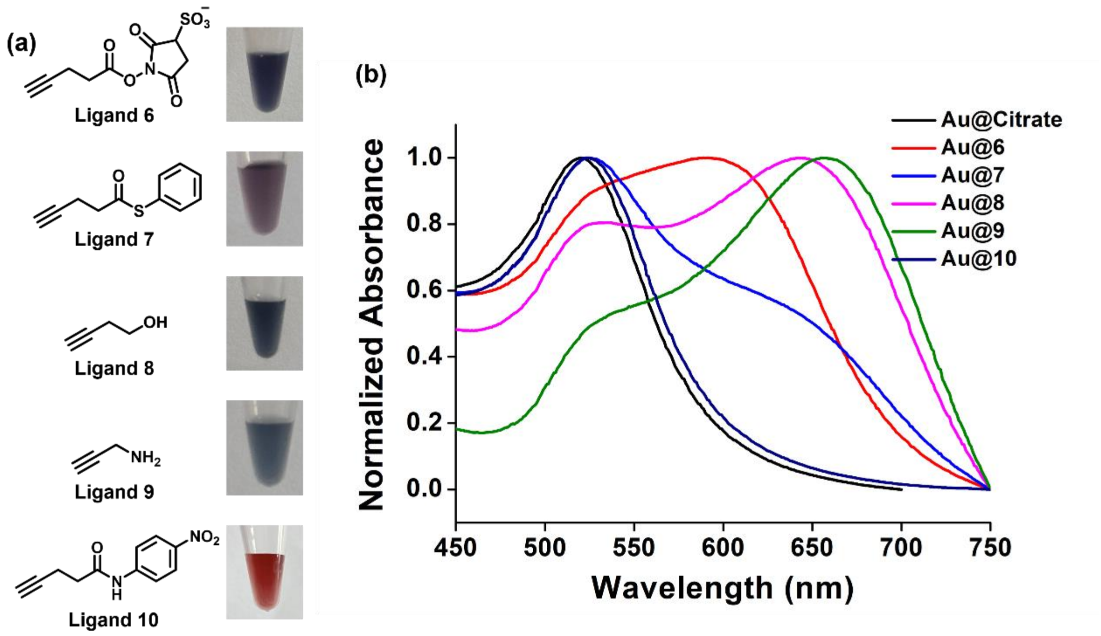


**Figure S2.** (a) Chemical structures of AuNP ligands and physical appearances of the corresponding ligand-capped AuNPs. (b) Normalized absorption spectra of the ligand-capped AuNPs, **Au@6** – **Au@10**.

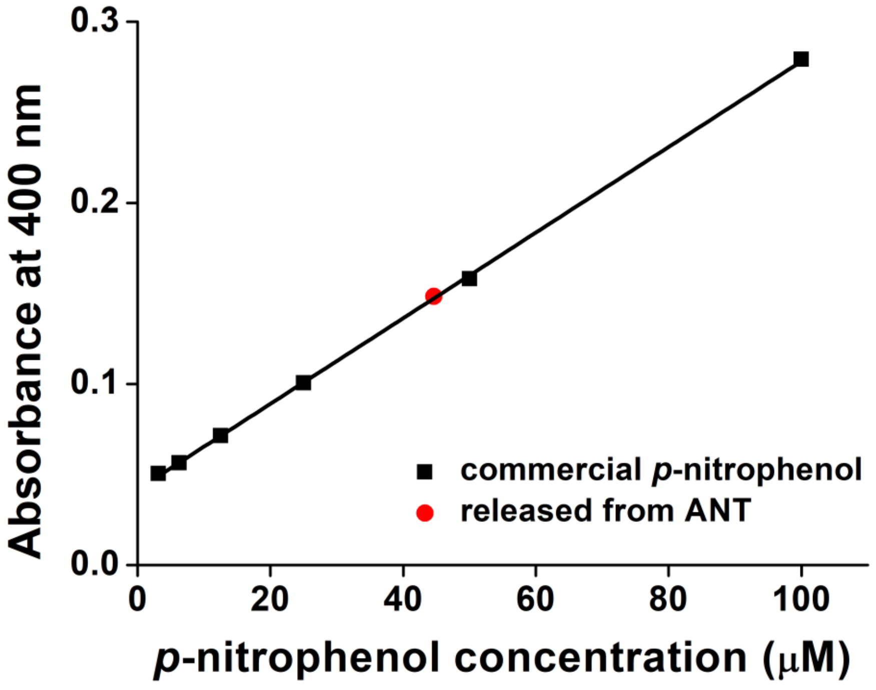


**Figure S3.** The calibration curve of *p*-nitrophenol in basic aqueous solution.

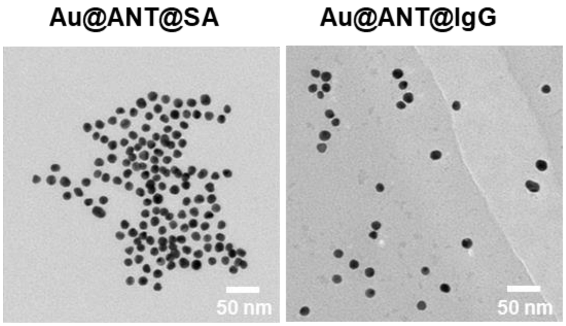


Figure S4. TEM images of **Au@ANT@SA** and **Au@ANT@IgG**.

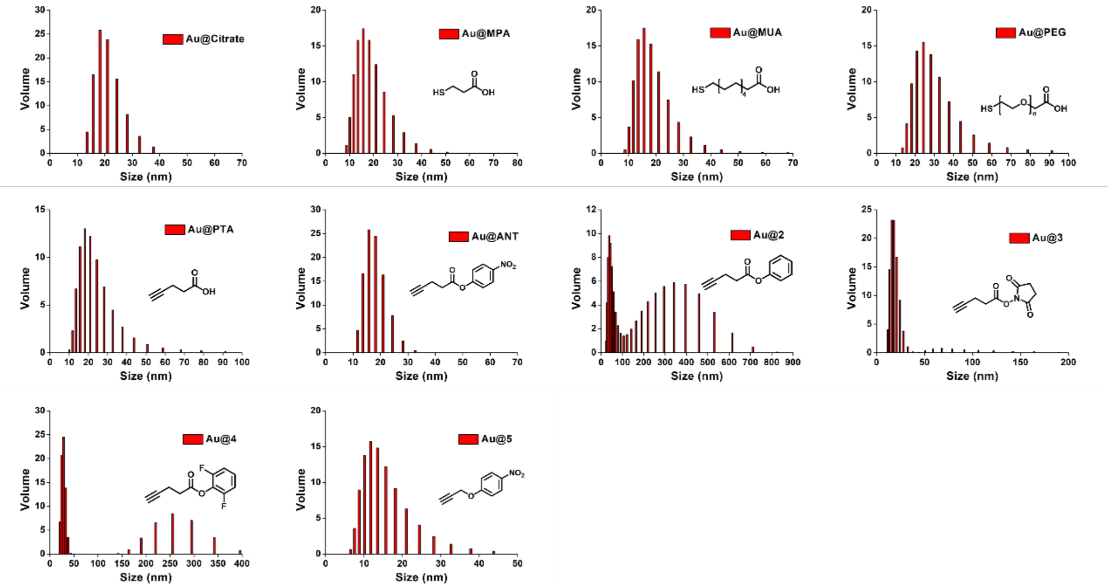


**Figure S5.** DLS size distributions of different ligand-capped AuNPs.

(a)

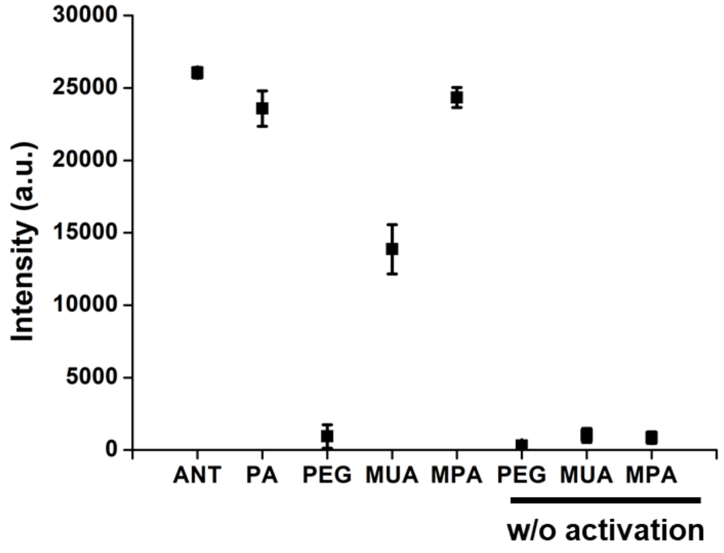


(b)

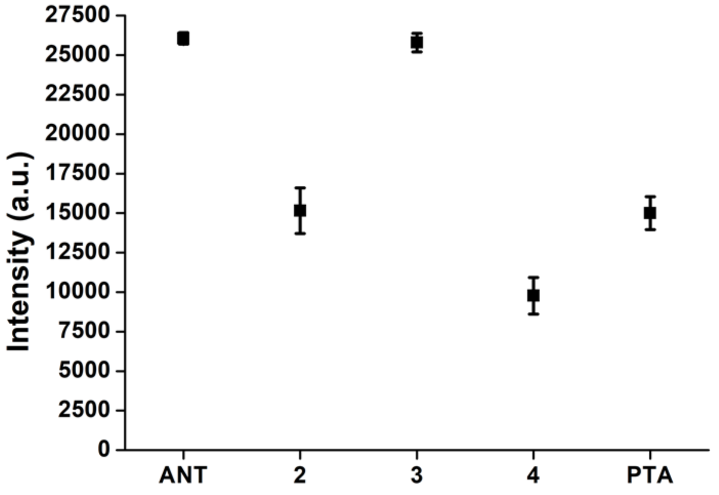


**Figure S6.** Quantitative analysis of the test line signal intensities corresponding to the LFA strips shown in (a) Figure 3a and (b) Figure 3b.

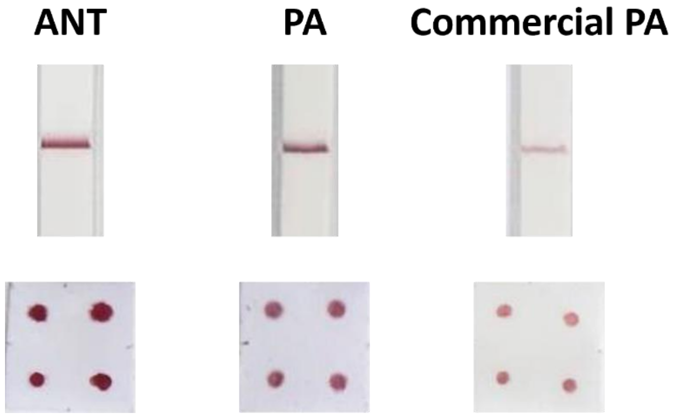


**Figure S7.** LFA and dot blot results of **Au@SA** prepared via **ANT**, PA, and compared with a commercial **Au@SA** conjugate. The number of **Au@SA** added to the detection solution was normalized to the same optical density (OD).

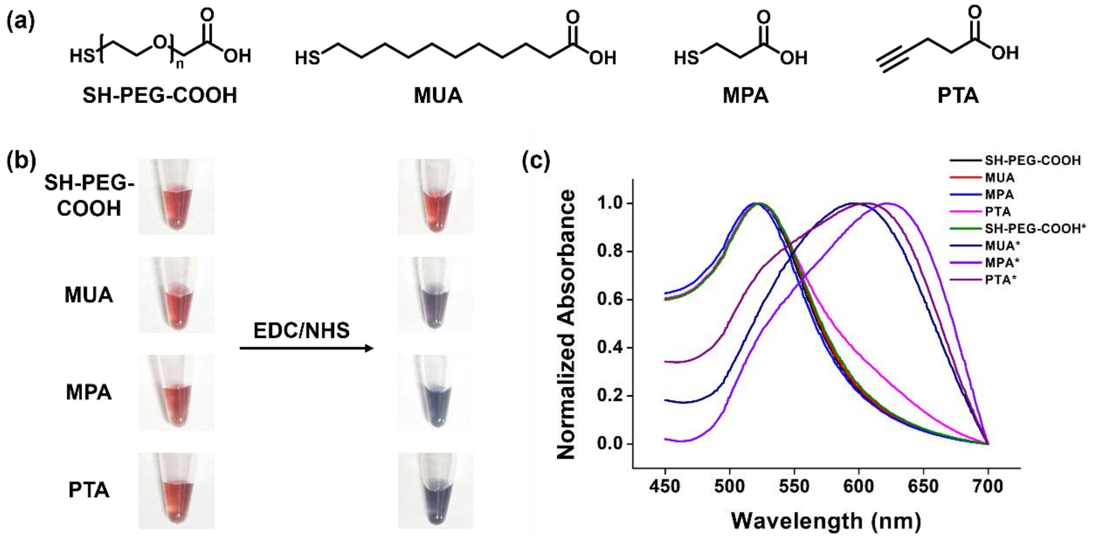


**Figure S8.** (a) Chemical structures of carboxylate ligands for AuNPs. (b) Photographs of AuNPs modified with different ligands and treated with 1 mM EDC/sulfo-NHS. (c) Absorption spectra of samples in (b); asterisks (*) indicate carboxylate capped AuNPs after treatment with EDC/NHS reagents.

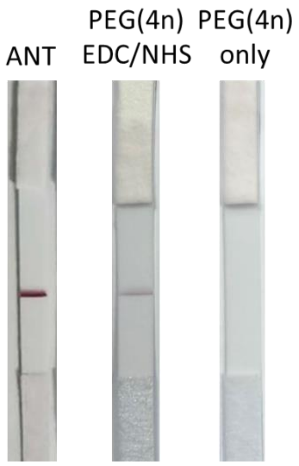


**Figure S9.** SA-AuNPs conjugates prepared using **ANT**, PEG(4n) with or without EDC/NHS coupling reagents. PEG(4n): short-chain PEG ligand containing four ethylene glycol units.

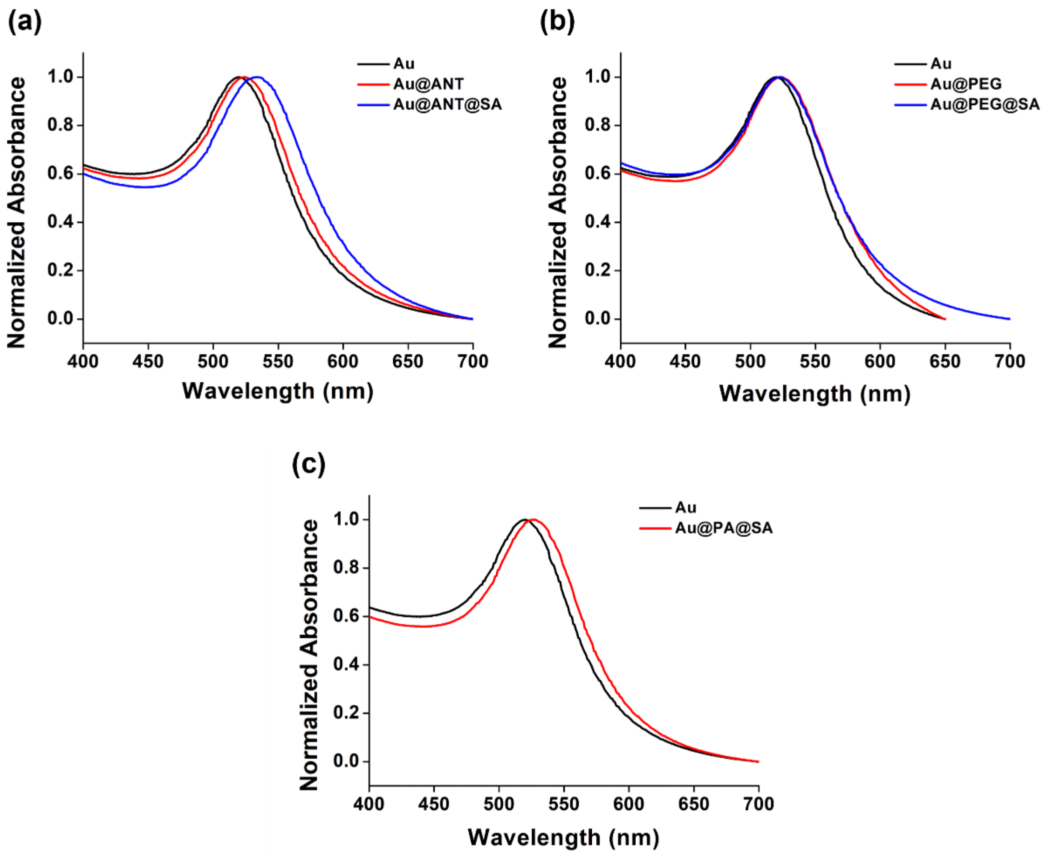


**Figure S10.** Absorption spectra of SA-AuNPs conjugates prepared via (a) **ANT**, (b) PEG (EDC/NHS) and (c) PA methods.

**Table S1.** Maximum absorption wavelength ($\lambda_{abs}$), ζ potential, and hydrodynamic diameter (Z-average) of SA- and IgG-functionalized AuNPs prepared using PA or ANT methods.

| Au@Protein | $\lambda_{abs}$ /nm | ζ-potential/mV | Z-average/nm |
|---|---|---|---|
| Au@PA@SA | 531 | -23.2 | 62.9 |
| Au@ANT@SA | 534 | -24.9 | 87.2 |
| Au@PA@IgG | 528 | -27.2 | 67.3 |
| Au@ANT@IgG | 526 | -29.6 | 59.4 |

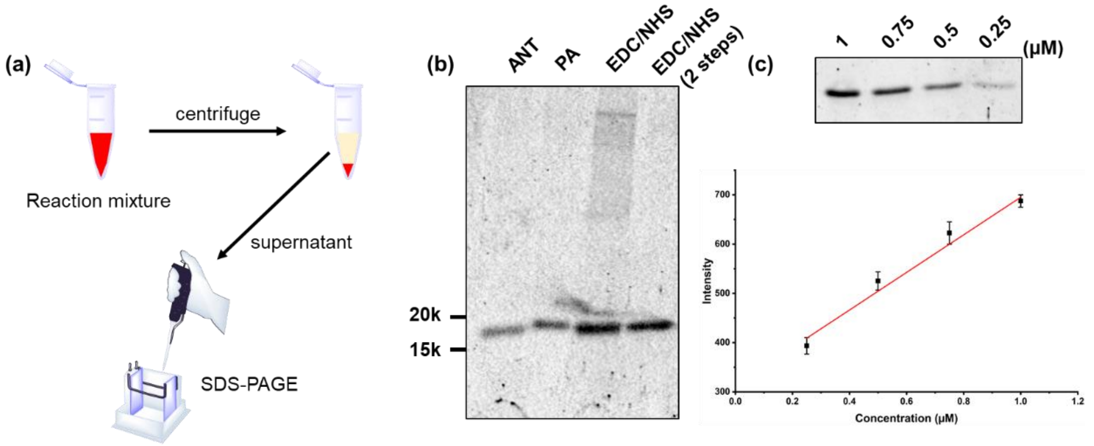


**Figure S11.** (a) Schematic illustration of the SDS–PAGE strategy for determining unreacted SA. (b) SDS–PAGE of unreacted SA collected from **ANT**, PA, and PEG (EDC/sulfo-NHS) conjugations (one-pot and two-step). Gels were stained with Coomassie blue and quantified using an in-gel scanner. (c) SDS–PAGE of SA standards (0.25–1 µM) used for quantification. In all conjugations, 1 µM SA was used. In the one-pot EDC/NHS approach, extensive protein smearing was observed due to interprotein cross-coupling. Among the methods tested, the **ANT** approach gave the weakest SA band, followed by PA and then EDC/NHS, indicating the highest conjugation efficiency for **ANT**.

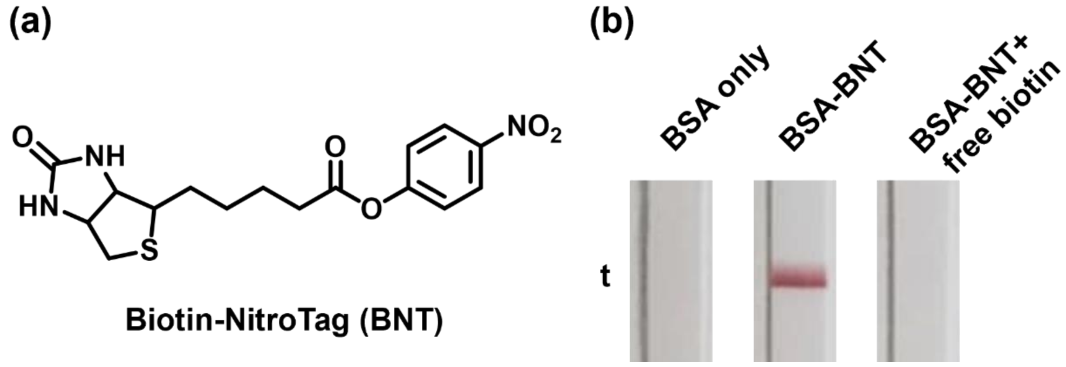


**Figure S12.** (a) Chemical structure of biotin-NitroTag (**BNT**). (b) LFA strips with test lines coated with BSA or BSA–BNT, probed with Au@SA conjugates. Free biotin (100 µM) was added as a competitor to confirm specific binding. The results demonstrate that the nitrophenyl ester is a sufficiently reactive electrophile for protein conjugation.

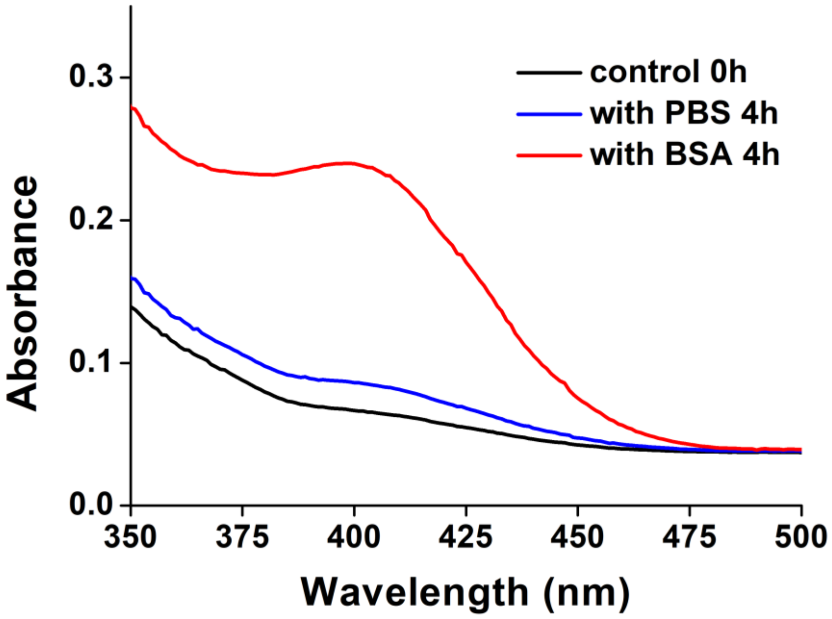


**Figure S13.** UV–vis absorption spectra of **ANT** treated with PBS (control) or with BSA for 4 h at 25 °C. The increased absorbance indicates release of *p*-nitrophenol from **ANT** upon nucleophilic substitution by BSA.

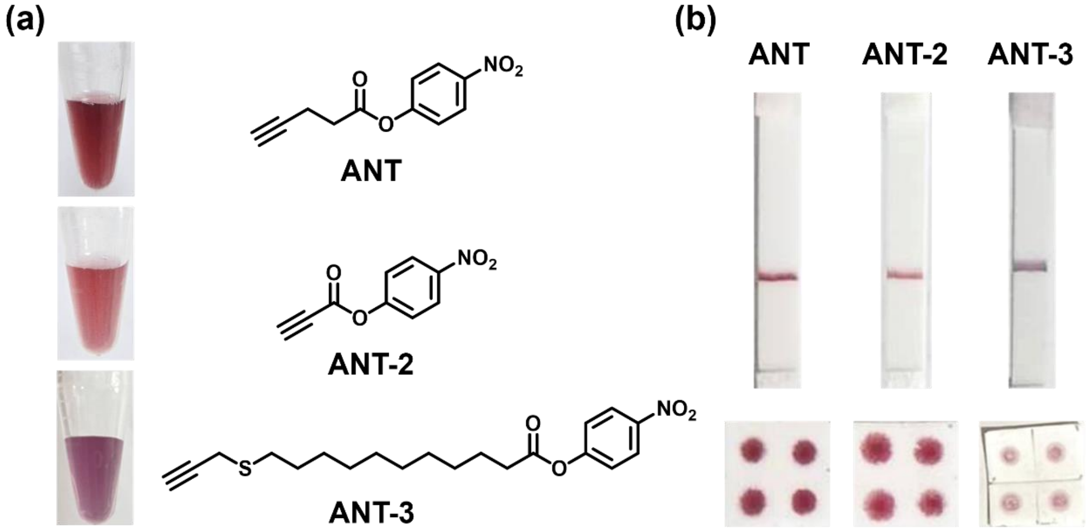


**Figure S14.** (a) Chemical structures of **ANT** analogues and photographs of the corresponding AuNP solutions. (b) LFA and dot blot results of **Au@SA** conjugates prepared with the **ANT** analogues. The concentration of SA used to prepare Au@ANT@SA and Au@PA@SA was 1 μM.

(a)

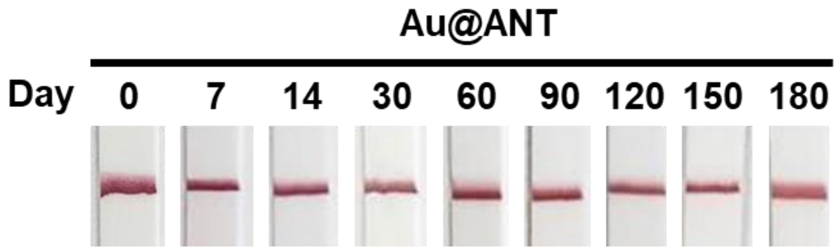


(b)

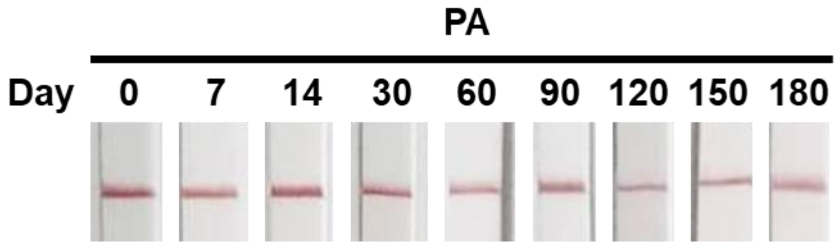


**Figure S15**. Long-term storage stability of (a) **Au@ANT@SA** and (b) **Au@PA@SA** in conjugate buffer at 4 °C for 180 days and analyzed by LFA test strips.

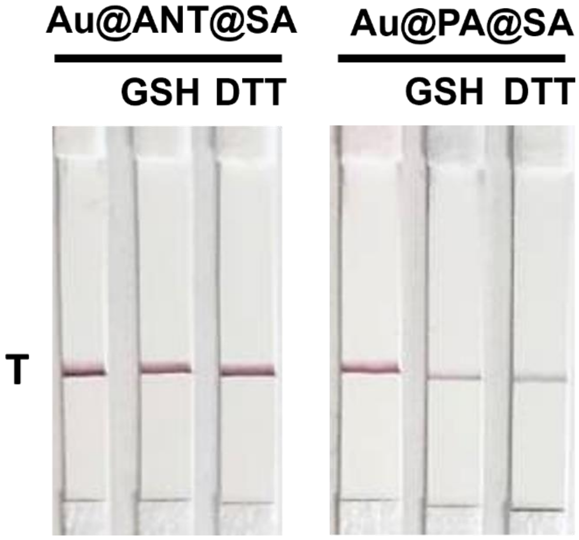


**Figure S16.** Representative LFA strips comparing the stability of **Au@ANT@SA** and **Au@PA@SA** conjugates after incubation with 5 mM glutathione (GSH) or 5 mM dithiothreitol (DTT) for 1 hour at 37 °C. The results indicate improve resistance of **Au@ANT@SA** toward reductive thiol environments. The concentration of SA used to prepare **Au@ANT@SA** and **Au@PA@SA** was 1 μM.

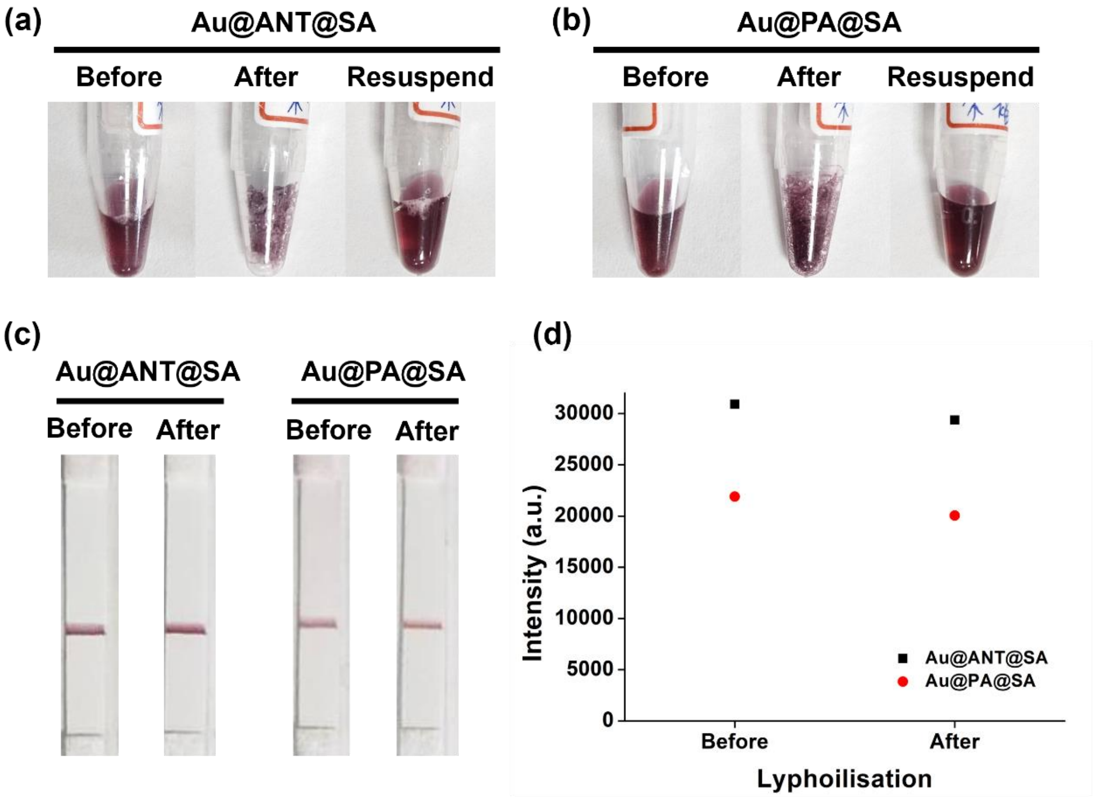


**Figure S17.** Photographs of (a) **Au@ANT@SA** and (b) **Au@PA@SA** before and after lyophilization. (c) LFA strips and (d) signal quantification comparing AuNP-protein conjugates before and after lyophilization.

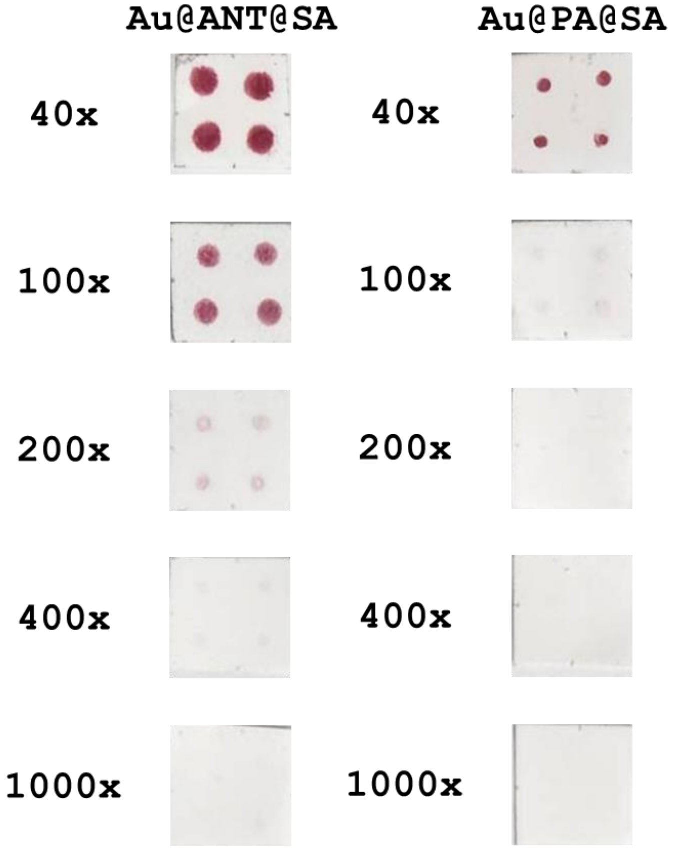


**Figure S18.** Comparison of the analytical sensitivity of **Au@ANT@SA** and **Au@PA@SA** conjugates for detecting biotinylated BSA via dot blot assay. Gold nanoparticle conjugates were subjected to serial dilution before application. **Au@ANT@SA** showed higher sensitivity, with visible signal retained at greater dilution levels compared to **Au@PA@SA**.

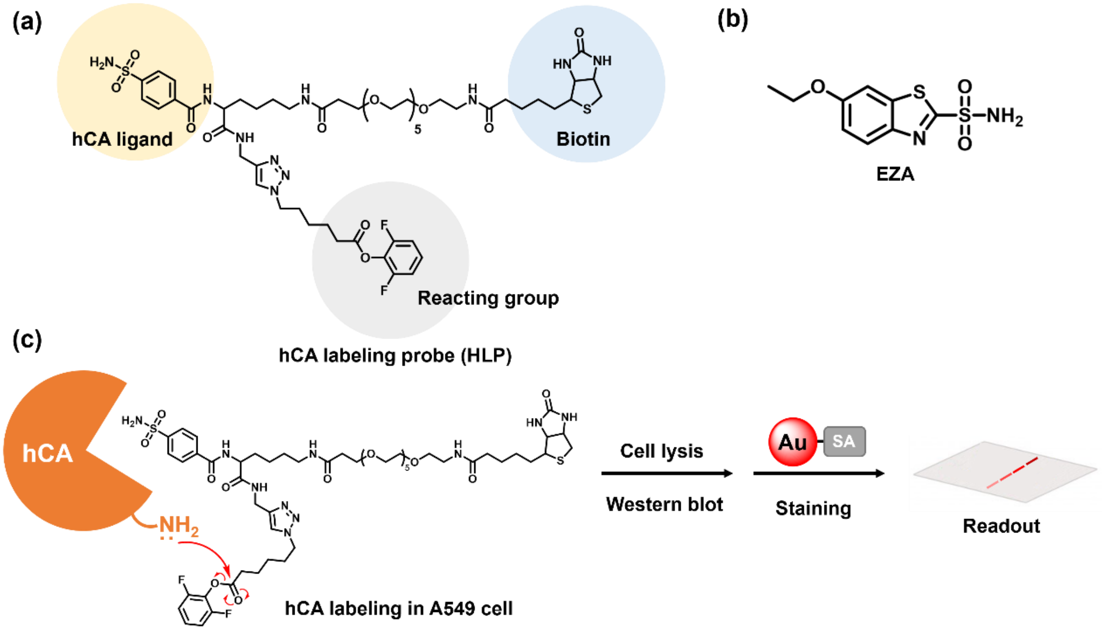


**Figure S19.** (a) Chemical structure of the hCA labeling probe (**HLP**). (b) Chemical structure of hCA inhibitor ethoxzolamide (EZA). (c) Labeling mechanism of **HLP** and Western blot detection using **Au@ANT@SA**.

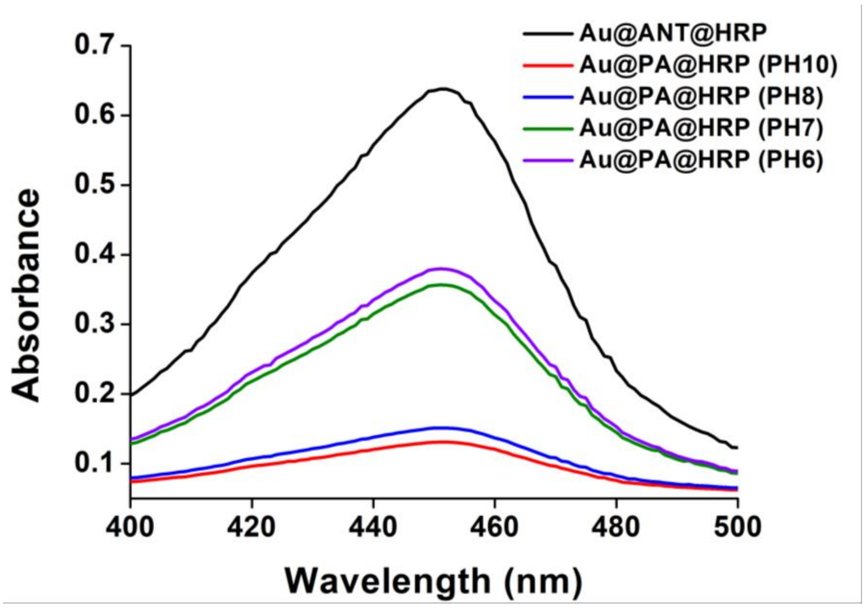


**Figure S20.** HRP conjugation efficiency of **Au@ANT@HRP** and **Au@PA@HRP** prepared under different pH solutions. To evaluate the HRP conjugations, the nanoparticles were incubated with $H_2O_2$ and TMB at 37 °C for 15 minutes and the reaction was quenched by addition of 100 µL of 3 M $H_2SO_4$. The oxidized TMB product (TMBDI) was analyzed using a UV–vis absorption spectrometer (TECAN Infinite M200 PRO).

**Table S2.** Kinetic parameters of native HRP and AuNP-conjugated HRP.

| No. | Sample | $V_{max}$ (μM/min) | $K_m$ (μM) | $K_{cat}$ ($min^{-1}$) | $K_{cat}/K_m$ ($min^{-1}μM^{-1}$) | Ref. |
|---|---|---|---|---|---|---|
| 1 | Native HRP | 23.4 | 209.2 | 23419 | 111.9 | This work |
| 2 | Au@ANT@HRP | 10.8 | 284.4 | 10799 | 38.0 | This work |
| 3 | Au@PA@HRP | 4.8 | 215.0 | 4778 | 22.2 | This work |
| 4 | Native HRP | 2.8 | 6. | 300 | 5.0 | 1 |
| 5 | Au@HRP | 1.0 | 77 | 700 | 9.1 | 1 |
| 6 | Native HRP | 2260 | 3850 | 50850 | 13.2 | 2 |
| 7 | Au@HRP | 5700 | 1810 | 128250 | 70.8 | 2 |
| 8 | Native HRP | -- | 476 | 23400 | 49.2 | 3 |
| 9 | CNP@HRP[a] | -- | 682 | 37620 | 55.2 | 3 |
| 10 | Native HRP | 5.23 | 3700 | 208800 | 56.4 | 4 |
| 11 | Native HRP | 5.23 | 3700 | 4560 | 1.2 | 5 |
| 12 | Native HRP | 7.26 | 276 | -- | -- | 6 |
| 13 | HRP-NF[b] | 5.85 | 220 | -- | -- | 7 |

[a] CNP: carbon nanoparticle

[b] NF: organic–inorganic hybrid nanoflowers

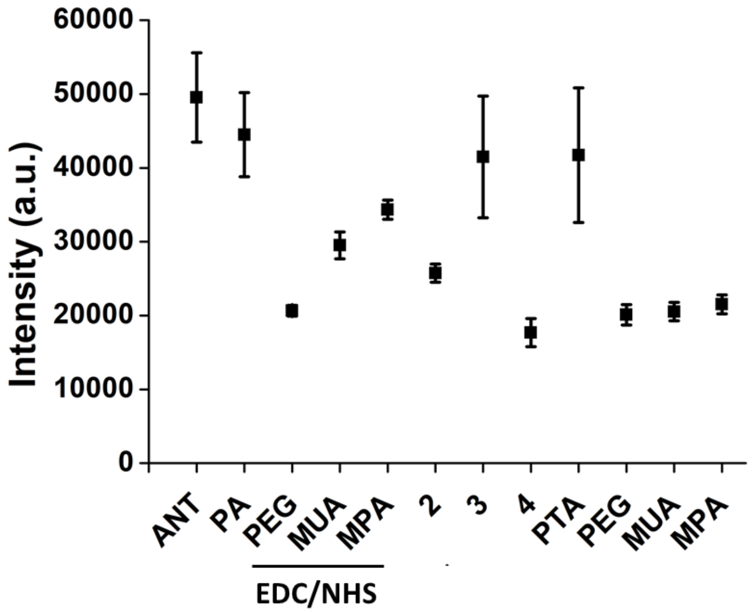


**Figure S21.** Quantitative analysis of the test line signal intensities corresponding to the LFA strips shown in Figure 6a in the main text.

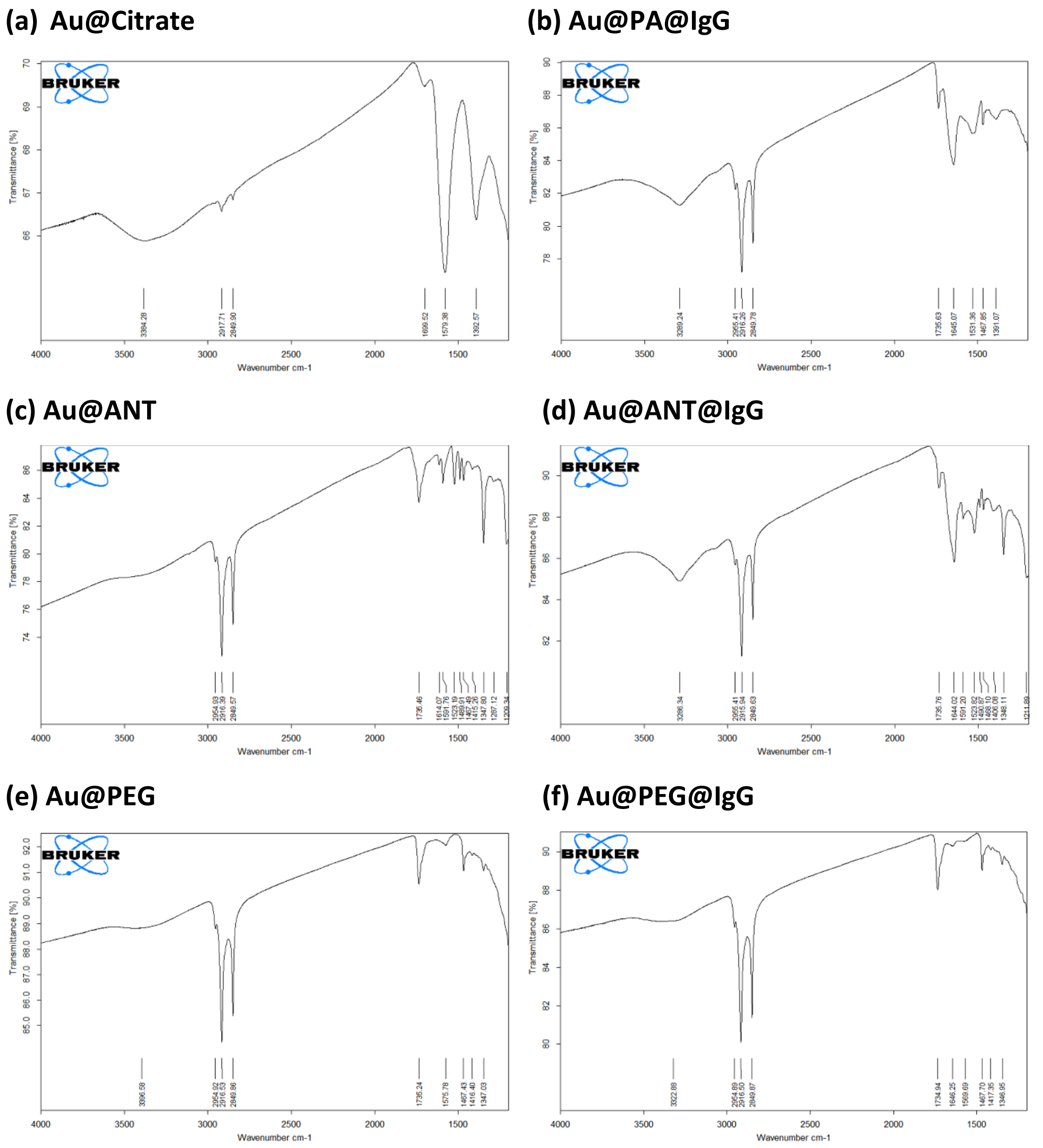


**Figure S22.** FTIR spectra of (a) **Au@citrate**, (b) **Au@PA@IgG**, (c) **Au@ANT**, (d) **Au@ANT@IgG**, (e) **Au@PEG**, and (f) **Au@PEG@IgG**.

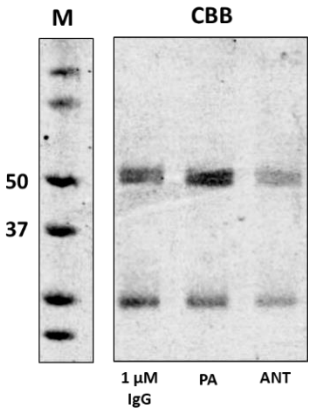


**Figure S23.** SDS-PAGE analysis of unreacted IgG remaining in the supernatant after conjugation using **ANT** and PA methods. Reduced IgG band intensity in the supernatant indicates higher conjugation efficiency for **ANT**.

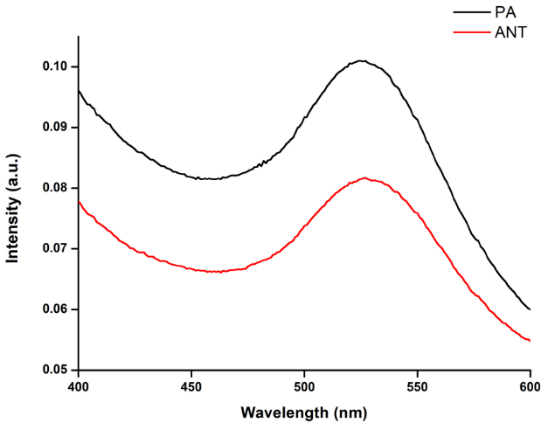


**Figure S24**. UV–vis absorption spectra of **Au@ANT@IgG** and **Au@PA@IgG** after conjugation and purification.

(a)

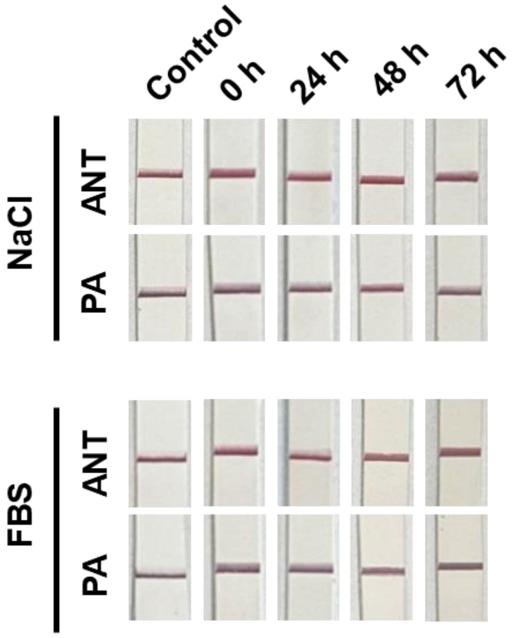


(b)

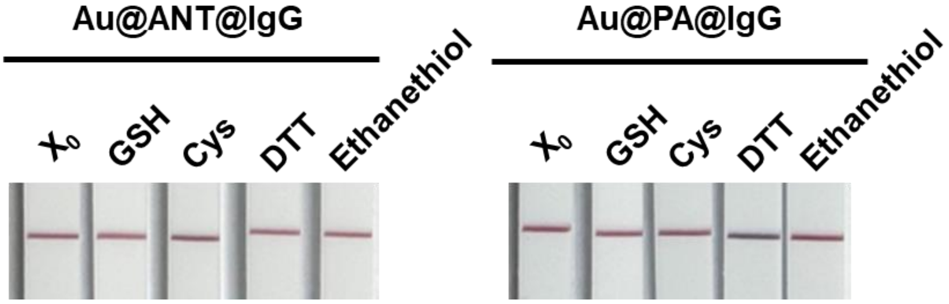


**Figure S25.** Stability of **Au@ANT@IgG** and **Au@PA@IgG** in (a) 60 % FBS and 1M NaCl, or (b) upon treatment with 0.5 mM thiolated compounds for 1 hour at 37 °C. **Au@PA@IgG** shows visible signs of aggregation after incubation with DTT for 1 hour at 37 °C.

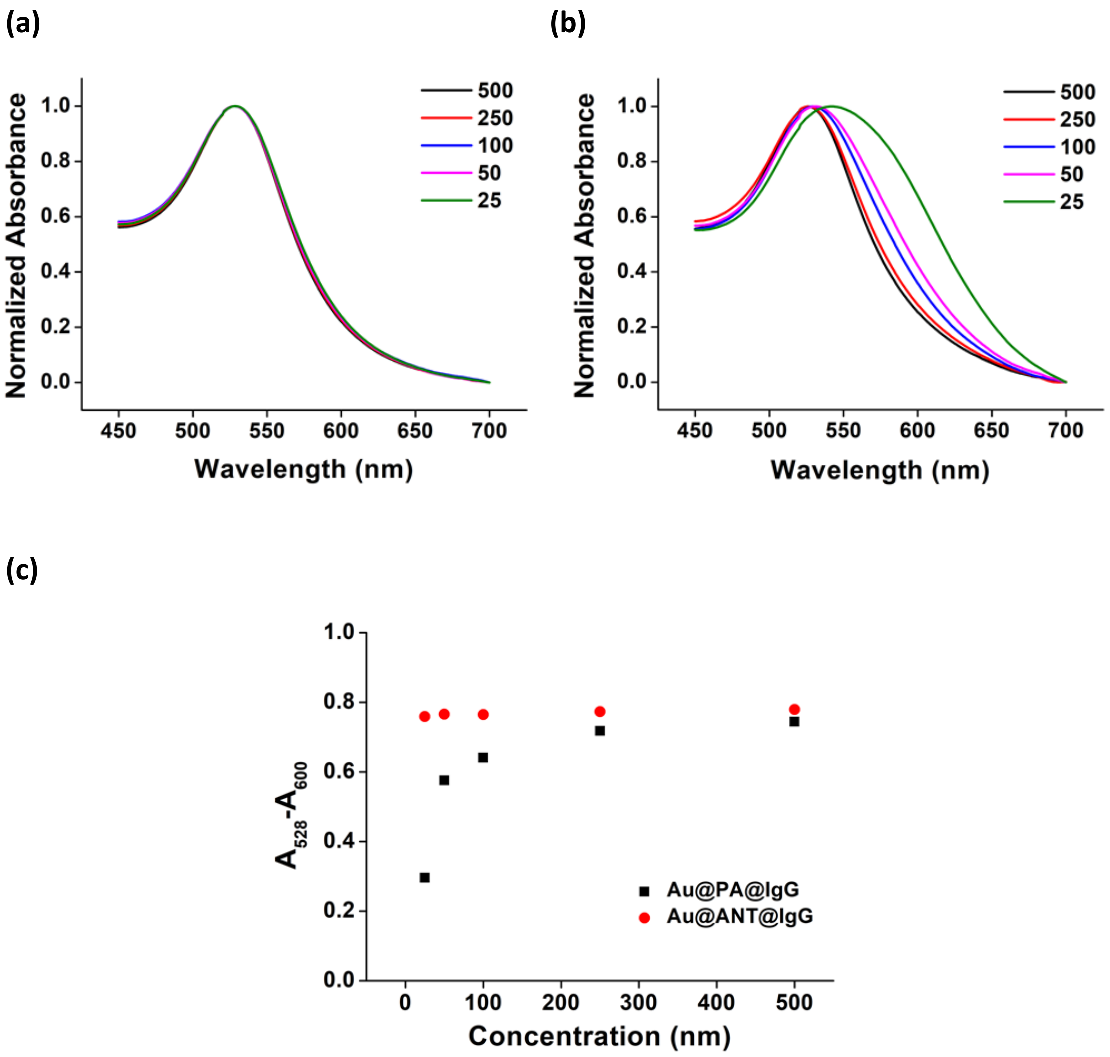


**Figure S26.** (a) Absorption spectra of **Au@ANT@IgG** corresponding to Figure 6b, confirming colloidal stability. (b) Absorption spectra of **Au@PA@IgG** corresponding to Figure 6b. (c) Absorbance shift (528 nm – 600 nm) of **Au@PA@IgG** and **Au@ANT@IgG** conjugates prepared using different IgG concentrations. The absorbance shift serves as an indicator of nanoparticle aggregation and can be used to determine the minimal antibody concentration required to stabilize gold nanoparticles. Aggregation leads to a red shift in the SPR peak and reduced absorbance at around 520 nm.

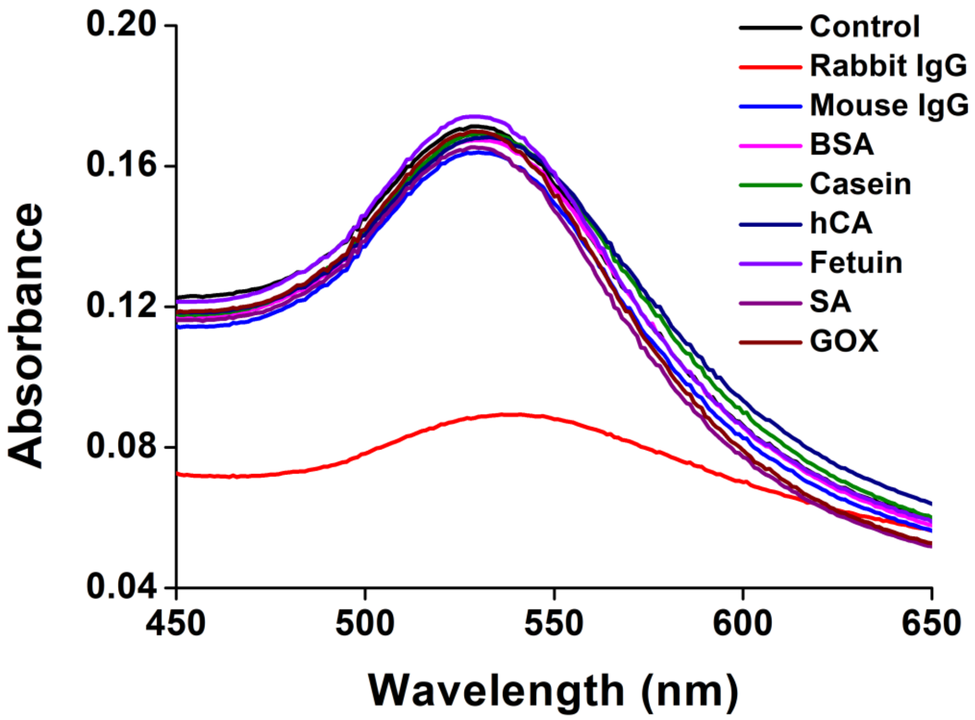


**Figure S27.** UV–vis absorption spectra of **Au@ANT@IgG** after incubation with the target rabbit IgG (9.1 nM) or with 91 nM non-target proteins.

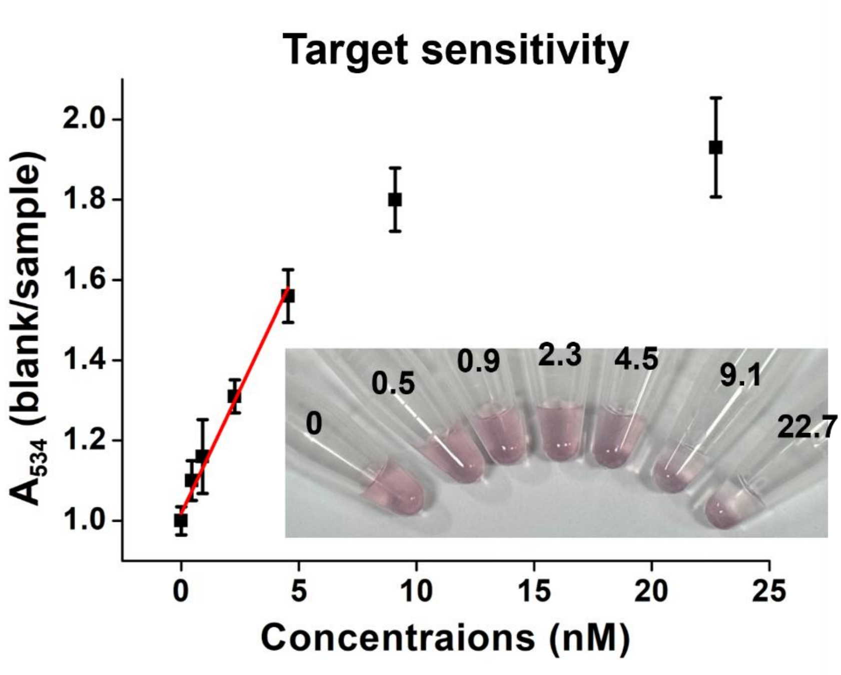


**Figure S28.** Precipitation assay of **Au@ANT@IgG** with rabbit IgG at different concentrations. The calibration curve demonstrates a linear response from 0 – 5 nM. y = 0.1183x + 1.0093, $R^2$ = 0.9973

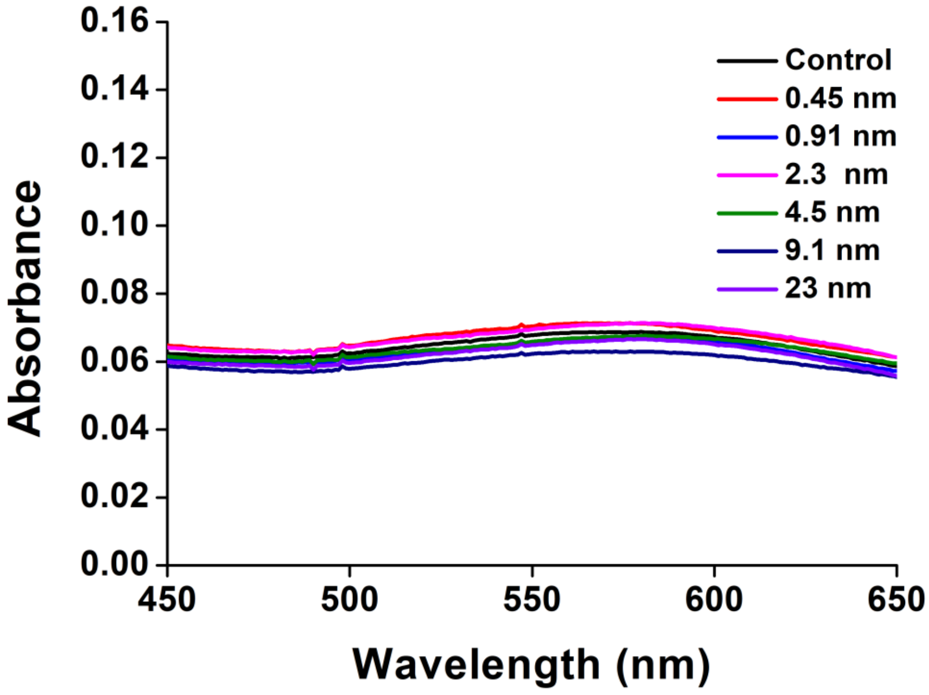


**Figure S29.** Precipitation assay of **Au@PA@IgG** upon addition of rabbit IgG at varying concentrations. **Au@PA@IgG** was prepared using the PA method with 25 nM IgG. Significant aggregation was observed even prior to target addition, indicating colloidal instability of the conjugates under these preparation conditions.

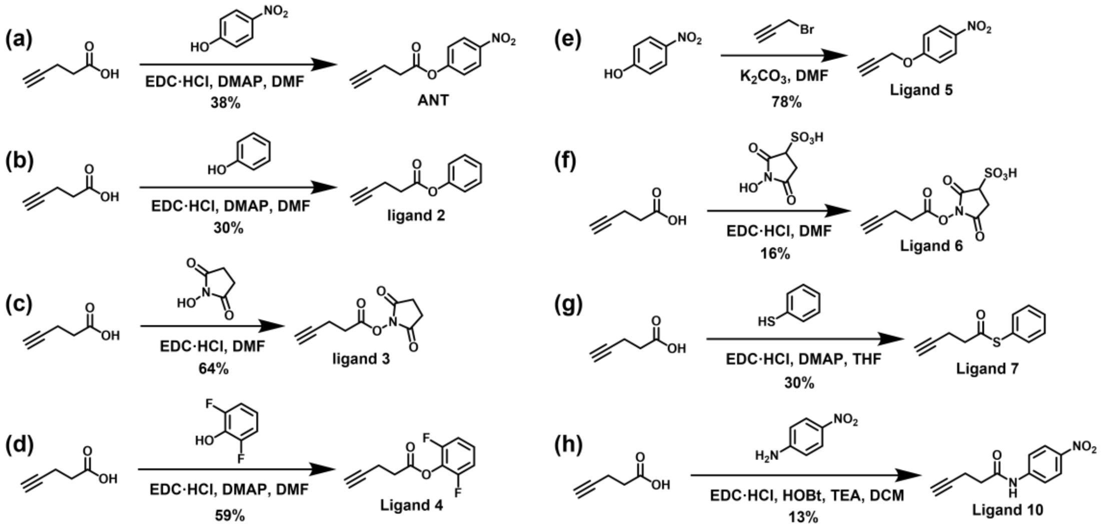


**Scheme S1.** Synthesis of **ANT** and other alkynylated ligands. (a) **ANT**, (b) Ligand **2**, (c) Ligand **3**, (d) Ligand **4**, (e) Ligand **5**, (f) Ligand **6**, (g) Ligand **7**, and (h) Ligand **10**.

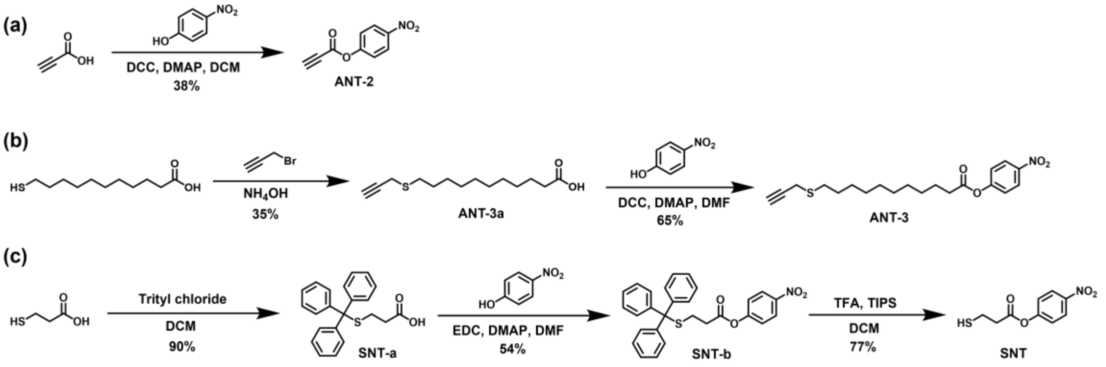


**Scheme S2.** Synthesis of **ANT** derivatives. (a) **ANT-2**, (b) **ANT-3** and (c) **SNT**.

## References:


[1] Männel, M. J.; Kreuzer, L. P.; Goldhahn, C.; Schubert, J.; Hartl, M. J.; Chanana, M., Catalytically Active Protein Coatings: Toward Enzymatic Cascade Reactions at the Intercolloidal Level. *ACS Catal.* **2017**, *7*, 1664.

[2] Siyal, L.; Kumar, B.; Kumar, R.; Sahney, R., Synthesis of Horseradish Peroxidase-Gold Nanoparticle Conjugate through Green Route. *Asian J. Chem.* **2020**, *32*, 1243.

[3] Ciaurriz, P.; Bravo, E.; Hamad-Schifferli, K., Effect of Architecture on the Activity of Glucose Oxidase/Horseradish Peroxidase/Carbon Nanoparticle Conjugates. *J. Colloid Interface Sci.* **2014**, *414*, 73.

[4] Gao, L.; Zhuang, J.; Nie, L.; Zhang, J.; Zhang, Y.; Gu, N.; Wang, T.; Feng, J.; Yang, D.; Perrett, S.; Yan, X., Intrinsic Peroxidase-Like Activity of Ferromagnetic Nanoparticles. *Nat. Nanotechnol.* **2007**, *2*, 577.

[5] Li, M.; Su, H.; Tu, Y.; Shang, Y.; Liu, Y.; Peng, C.; Liu, H., Development and Application of an Efficient Medium for Chromogenic Catalysis of Tetramethylbenzidine with Horseradish Peroxidase. *ACS Omega* **2019**, *4*, 5459.

[6] Shi, W.; Wang, Q.; Long, Y.; Cheng, Z.; Chen, S.; Zheng, H.; Huang, Y., Carbon Nanodots as Peroxidase Mimetics and Their Applications to Glucose Detection. *Chem. Commun.* **2011**, *47*, 6695.

[7] Dadi, S.; Temur, N.; Gul, O. T.; Yilmaz, V.; Ocsoy, I., In Situ Synthesis of Horseradish Peroxidase Nanoflower@Carbon Nanotube Hybrid Nanobiocatalysts with Greatly Enhanced Catalytic Activity. *Langmuir* **2023**, *39*, 4819.

**Synthesis of ANT.** To a stirred solution of 4-pentynoic acid (50 mg, 0.51 mmol) in 3 mL DMF was added EDC (194 mg, 1.02 mmol) and DMAP (31 mg, 0.26 mmol) sequentially at room temperature. After 15 minutes, 4-nitrophenol (142 mg, 1.05 mmol) was added, and the reaction mixture was stirred at room temperature and monitored by TLC (DCM, $R_f$ = 0.8). Upon completion, the mixture was diluted with EA (5 mL) and washed with $H_2O$ (5 mL) and brine (5 mL). The organic layer was collected, dried over anhydrous $Na_2SO_4$, and concentrated by rotary evaporation. The crude product was purified by flash column chromatography (DCM) to afford **ANT** as pale-yellow powder in 38% yield (42.3 mg).

**$^1$H NMR** (400 MHz, $CDCl_3$) δ 8.26 (d, *J* = 9.3 Hz, 2H), 7.29 (d, *J* = 9.3 Hz, 2H), 2.85 (t, *J* = 7.2, 7.2 Hz, 2H), 2.63 (td, *J* = 4.5, 2.6 Hz, 2H), 2.05 (t, *J* = 2.6 Hz, 1H) ppm.

**$^{13}$C NMR** (101 MHz, $CDCl_3$) δ 169.40, 155.25, 145.43, 125.23, 122.42, 81.63, 69.77, 33.47, 14.36 ppm.

**Synthesis of ligand 2.** To a stirred solution of 4-pentynoic acid (50 mg, 0.51 mmol) in 3 mL of DMF was added EDC (194 mg, 1.02 mmol) and DMAP (31 mg, 0.26 mmol) sequentially at room temperature. After 15 minutes, phenol (96 mg, 1.02 mmol) was added, and the reaction mixture was stirred overnight. The reaction was monitored by TLC (HEX/EA= 3:1, $R_f$ = 0.6). Upon completion, the mixture was diluted with EA (5 mL) and washed with $H_2O$ (5 mL) and brine (5 mL). The organic layer was collected, dried over anhydrous $Na_2SO_4$, and concentrated by rotary evaporation. The crude product was purified by flash column chromatography (HEX/EA) to afford ligand **2** as colorless oil in 30% yield (26.1 mg).

**$^1$H NMR** (400 MHz, $CDCl_3$) δ 7.40 – 7.36 (m, 2H), 7.24 (tt, *J* = 17.2, 1.2 Hz, 1H), 7.12 – 7.0974(m, 2H), 2.82(t, *J* = 7.2 Hz, 2H), 2.63 (td, *J* = 17.3, 2.7 Hz, 2H), 2.05 (t, *J* = 2.6 Hz, 1H) ppm.

**$^{13}$C NMR** (101 MHz, $CDCl_3$) δ 170.31, 150.60, 129.45, 125.94, 121.51, 82.08, 69.43, 33.52, 14.49 ppm.

**Synthesis of ligand 3.** To a stirred solution of 4-pentynoic acid (30 mg, 0.26 mmol) in 5 mL of DMF was added EDC (147 mg, 0.76 mmol) and N-hydroxysuccinimide (24 mg, 0.24 mmol). The reaction was monitored by HPLC. Upon completion, DMF was

removed under reduced pressure. The crude product was purified by reverse-phase column chromatography (gradient 10% ACN/90% $H_2O$ to 100% ACN for 21 min) to afford ligand **3** as white powder in 64% yield (30.0 mg).

**$^1$H NMR** (400 MHz, $CDCl_3$) δ 2.86 (t, *J* = 7.2, 7.7HZ, 2H), 2.82 (s, 4H), 2.59 (td, *J* = 17.5, 2.6 Hz, 2H), 2.04 (t, *J* = 2.6, 2.6 Hz, 1H) ppm.

**$^{13}$C NMR** (101 MHz, $CDCl_3$) δ 175.27, 172.83, 87.17, 77.34, 34.80, 30.60, 18.63 ppm.

**Synthesis of ligand 4.** To a stirred solution of 4-pentynoic acid (50 mg, 0.51 mmol) in 5 mL of DMF was added EDC (147 mg, 0.76 mmol) and DMAP (31 mg, 0.5 mmol). After 5 minutes, 2,6-difluorophenol (132 mg, 1.02 mmol) was added and the mixture was stirred overnight. The reaction was monitored by TLC (HEX:EA= 4:1, Rf= 0.6). Upon completion, DMF was removed under reduced pressure. The crude product was purified by flash column chromatography (DCM) to afford ligand **4** as pale-yellow powder in 59% yield (63.2 mg).

**$^1$H NMR** (400 MHz, $CDCl_3$) δ 7.15 – 7.07 (m, 1H), 6.97 – 6.88 (m, 2H), 2.87 (t, *J* = 7.6 Hz, 2H), 2.60 (td, J = 3.1, 7.7 Hz, 2H), 2.03 (t, *J* = 2.6 Hz, 1H) ppm.

**$^{13}$C NMR** (101 MHz, $CDCl_3$) δ 168.28, 156.29 (d, *J* = 4.2 Hz), 153.80 (d, *J* = 4.2 Hz), 126.93 (t, *J* = 16.1 Hz), 126.37 (t, J = 9.1 Hz), 111.90 (dd, *J* = 17.0, 5.0 Hz), 81.48, 69.47, 32.55, 14.16 ppm.

**$^{19}$F NMR** (471 MHz, $CDCl_3$) δ 126.23 ppm.

**Synthesis of ligand 5.** To a stirred solution of 4-pentynoic acid (25.5 mg, 0.21mmol) in 5 mL dry DMF was added potassium carbonate (74 mg, 0.54 mmol). After 30 minutes, 3-bromoprop-1-yne (15 mg, 0.11 mmol) was added, and the mixture was stirred overnight. The reaction was monitored by TLC (HEX/EA= 4:1, $R_f$ = 0.6). Upon completion, the mixture was diluted with EA (5 mL) and washed with $H_2O$ (5 mL) and brine (5 mL). The organic layer was collected, dried over anhydrous $Na_2SO_4$, and concentrated by rotary evaporation. The crude product was purified by flash column chromatography (HEX/EA) to afford ligand **5** as colorless oil in 78% yield (15.1 mg).

**$^1$H NMR** (400 MHz, $CDCl_3$) δ 8.19 (d, *J* = 9.4 Hz, 2H), 7.03 (d, *J* = 9.3 Hz, 2H), 4.77 (d, *J* = 2.4 Hz, 2H), 2.56 (t, *J* = 2.4, 2.3 Hz, 1H) ppm.

**$^{13}$C NMR** (101 MHz, $CDCl_3$) δ 162.34, 142.16, 125.84, 114.99, 56.31, 29.70 ppm.

**HRMS** (ESI): m/z calc. for $[C_9H_8NO_3]^+$ $[M+H]^+$ 178.05042 found 178.04993

**Synthesis of ligand 6.** To a stirred solution of 4-Pentynoic acid (45 mg, 0.46 mmol) in 5 mL DMF was added EDC·HCl (106 mg, 0.55 mmol). After 5 minutes, 1-hydroxy-2,5-dioxopyrrolidine-3-sulfonic acid (100 mg, 0.46 mmol) was added, and then the mixture was stirred overnight. The reaction was monitored by HPLC. After the reaction was completed, DMF was removed under reduced pressure. The residue was purified by reverse-phase column chromatography (gradient 10% ACN/90% $H_2O$ to 100% ACN for 21 min) to afford ligand **6** as pale-yellow oil in 16% yield (22.0 mg).

**$^1$H NMR** (400 MHz, CDCl3) δ 4.24 – 4.21 (m, 1H), 3.11 – 2.96 (m, 2H), 2.77 (t, *J* = 5.28, 7.32 Hz, 2H), 2.50 (t, *J* = 6.24, 6.16 Hz, 2H), 2.14 (s, 1H) ppm.

**Synthesis of ligand 7.** To a stirred solution of 4-Pentynoic acid (50 mg, 0.51 mmol) in 3 mL THF was added EDC·HCl (146 mg, 0.76 mmol) and DMAP (mg, 0.5 mmol) sequentially at room temperature. After 5 minutes, thiophenol (30 mg, 0.25 mmol) was added, and the mixture was stirred overnight. The reaction was monitored by TLC (HEX/EA= 4:1, $R_f$ = 0.7). After the reaction was complete, the solvent was removed by rotary evaporation. The mixture was then dissolved in DCM (5 mL) and was successively washed with $H_2O$ (3×5 mL), brine (5 mL) and dried over anhydrous $Na_2SO_4$. The organic solvent was removed by rotary evaporation. Subsequently, the residue was purified by flash column chromatography (HEX/EA) to afford ligand **7** as pale-yellow oil in yield 30% (26.1 mg).

**$^1$H NMR** (400 MHz, $CDCl_3$) δ 7.40 – 7.39 (m, 5H), 2.88 (t, *J* = 7.3, 7.6 Hz, 2H), 2.55 (td, *J* = 2.6, 7.7 Hz, 2H), 1.99 (t, *J* = 2.6, 2.7 Hz, 1H) ppm.

**$^{13}$C NMR** (101 MHz, $CDCl_3$) δ 195.54, 134.51, 129.55, 129.26, 127.32, 81.84, 69.52, 42.06, 14.60 ppm.

**HRMS** (ESI): m/z calc. for $[C_{11}H_{11}OS]^+$ $[M+H]^+$ 191.05306 found 191.05303.

**Synthesis of ligand 10.** To a stirred solution of 4-Pentynoic acid (50 mg, 0.51 mmol) in 10 mL DCM was added EDC·HCl (194 mg, 1.02 mmol), DMAP (6.2 mg, 0.05 mmol), TEA (77 mg, 0.76 mmol) and 4-nitroaniline (140 mg, 1.02 mmol) sequentially at ice bath.

The reaction was then stirred at room temperature overnight. The reaction was monitored by TLC (HEX:EA= 5:1, Rf= 0.5). After the reaction was completed, the mixture was washed with 1M HCl (3×5 mL), brine (5 mL) and dried over anhydrous $Na_2SO_4$. The organic solvent was removed by rotary evaporation. Subsequently, the residue was purified by flash column chromatography (HEX/EA) to afford ligand **10** as pale-yellow powder in 13% yield (14.4 mg)

**$^1$H NMR** (400 MHz, MeOD) δ 8.18 (d, *J* = 9.3 Hz, 2H), 7.79 (d, *J* = 9.4 Hz, 2H), 2.62 (t, *J* = 8.8, 6.8 Hz, 2H), 2.60 (td, *J* = 8.6, 2.1 Hz, 2H), 2.26 (t, *J* = 2.8, 2.5 Hz, 1H) ppm.

**$^{13}$C NMR** (101 MHz, MeOD) δ 171.34, 144.68, 143.16, 124.35, 118.92, 81.99, 68.95, 35.55, 13.83 ppm.

**HRMS** (ESI): m/z calc. for $[C_{11}H_9N_2O_3]^-$ $[M-H]^-$ 217.0619 found 217.0618.

**Synthesis of ANT-2.** To a stirred solution of propiolic acid (50 mg, 0.71 mmol) in 3 mL DCM was added 4-nitrophenol (142 mg, 1.05 mmol), DCC (197 mg, 1.42 mmol) and DMAP (2.6 mg, 2.6 mmol) sequentially at 0°C. Then the mixture was stirred at room temperature overnight. The reaction was detected by TLC (Hex/EA= 5:1, $R_f$=0.8). After the reaction was complete, the mixture was successively wash with HCl (0.1 N, 3×5 mL) and NaCl (3×5 mL), then the mixture finally dryed over anhydrous $Na_2SO_4$. The organic solvent was removed by rotary evaporation. Subsequently, the residue was purified by flash column chromatography (Hex/EA) to afford **ANT-2** as pale-yellow powder in yield 38% (42.3 mg)

**$^1$H NMR** (400 MHz, $CDCl_3$) δ 8.28 (d, *J* = 9.3 Hz, 2H), 7.34 (d, *J* = 9.3 Hz, 2H), 3.15 (s, 1H) ppm.

**$^{13}$C NMR** (101 MHz, $CDCl_3$) δ 154.34, 149.80, 146.01, 125.53, 122.38, 78.20, 73.70 ppm.

**Synthesis of ANT-3-a.** To a stirred solution of 11-mercaptoundecanoic acid (109 mg, 0.5 mmol) in 5 mL $NH_4OH$ (2 M) in ice bath. After 30 minutes, the mixture was cooled down to room temperature. Propargyl bromide (30 mg, 0.25 mmol) was added, and then the mixture was stirred for 3 hours. The reaction was detected by TLC (DCM:MeOH= 95:5, Rf= 0.45). After the reaction was completed, the solvent was removed under reduced pressure. The residue was washed by ethanol to afford **ANT-3-a** as pale-yellow solid in 35% yield (22.3 mg).

**$^{1}$H NMR** (400 MHz, $CDCl_3$) δ 3.21 (d, *J* = 2.6 Hz, 2H), 2.65 (t, *J* = 7.6, 7.2 Hz, 2H), 2.31 (t, *J* = 7.7, 7.3 Hz, 2H), 2.19 (t, *J* = 2.6, 2.5 Hz, 1H), 1.64 – 1.56, 1.34 –1.25 ppm.

**$^{13}$C NMR** (101 MHz, $CDCl_3$) δ 180.39, 80.17, 70.77, 34.11, 31.59, 29.40, 29.32, 29.19, 29.14, 29.02, 28.91, 28.78, 24.64, 19.13 ppm.

**HRMS** (ESI): m/z calc. for $[C_{14}H_{23}O_2S]^-$ $[M-H]^-$ 255.1424 found 255.1415.

**Synthesis of ANT-3.** To a stirred solution of ANT-3-A (20 mg, 0.8 mmol) in 5 mL DCM was added DCC (24 mg, 0.12 mmol) and DMAP (4.7 mg, 0.04 mmol). After 15 minutes, 4-nitrophenol (21 mg, 0.16 mmol) was added, and then the mixture was stirred for 3 hours. The reaction was detected by TLC (DCM, Rf= 0.8). After the reaction was completed, the solvent was removed under reduced pressure. The mixture was filtered to collect the precipitate. The mixture was then dissolved EA (5 mL) and was successively washed with $NaHCO_3$ (5 mL), HCl (1 M, 5 mL) and finally dry over anhydrous $Na_2SO_4$. The organic solvent was removed by rotary evaporation. Subsequently, the residue was purified by flash column chromatography (DCM) to afford **ANT-3** as a pale-yellow powder in 65% yield (19.2 mg)

**$^{1}$H NMR** (400 MHz, $CDCl_3$) δ 8.25 (d, *J* = 9.2 Hz, 2H), 7.25 (d, *J* = 9.4 Hz, 2H), 3.22 (d, *J* = 2.6 Hz, 2H), 2.66 (t, *J* = 7.6, 7.3 Hz, 2H), 2.57 (t, *J* = 7.5, 7.4 Hz, 2H), 2.20 (t, *J* = 2.6, 2.5 Hz, 1H), 1.77 – 1.70 (m, 2H), 1.63 – 1.58 (m, 2H), 1.40 – 1.28 (m, 12H) ppm.

**$^{13}$C NMR** (101 MHz, $CDCl_3$) δ 171.28, 155.53, 145.27, 125.19, 122.43, 80.18, 70.76, 34.33, 31.63, 29.39, 29.33, 29.18, 29.02, 28.92, 28.78, 24.72, 19.17 ppm.

HRMS (ESI): m/z calc. for [C20H28NO4S]+ [M+H]+ 378.1734 found 378.1733.

**Synthesis of ligand SNT-a.** To a stirred solution of 3-mercaptopropanoic acid (500 mg,4.72 mmol) in 5 mL DCM was added trityl chloride (1312 mg, 4.72 mmol) at room temperature. Then the mixture was stirred overnight. The reaction was monitored by TLC (HEX/EA= 4:1, $R_f$= 0.3). After the reaction was complete, the mixture was then filtered to collect the solid product to afford **SNT-a** as white powder in 90% yield (1477 mg).

**$^{1}$H NMR** (400 MHz, $CDCl_3$) δ 7.36 – 7.30 (m, 12H), 7.27 – 7.22 (m, 3H), 2.28 (t, *J* = 5.7, 8.5, 2H), 2.16 (t, *J* = 7.2, 8.5, 2H) ppm.

**$^{13}$C NMR** (101 MHz, $CDCl_3$) δ 173.18, 144.83, 129.56, 128.52, 127.21, 66.64, 33.36,

27.16 ppm.

**Synthesis of Compound SNT-b.** To a stirred solution of **SNT-A** (100 mg, 0.29 mmol) in 2 mL DMF was added EDC (110 mg, 0.57 mmol) and DMAP (17 mg, 0.14 mmol) at room temperature. After 5 minutes, 4-nitrophenol (79 mg, 0.57 mmol) was added, and the mixture was stirred overnight. The reaction was monitored by TLC (DCM, $R_f$= 0.8). Upon completion, the mixture was then dissolved in EA (5 mL) and was successively wash with brine (3×5 mL) and finally dried over anhydrous $Na_2SO_4$. The organic solvent was removed by rotary evaporation. Subsequently, the residue was purified by flash column chromatography (DCM) to afford **SNT-b** as white powder in 54% yield (73 mg).
**$^1$H NMR** (400 MHz, $CDCl_3$) δ 8.27(d, *J* = 7.4 Hz, 2H), 7.52 – 7.50 (m, 6H), 7.36 – 7.33 (m, 6H), 7.29 – 7.26 (m, 5H), 2.65 (t, *J* = 5.88, 5.56 Hz, 2H), 2.53 (t, *J* = 5.56, 5.76 Hz, 2H) ppm.
**$^{13}$C NMR** (101 MHz, $CDCl_3$) δ 169.45, 155.29, 145.38, 144.51, 129.60, 128.07, 126.89, 125.21, 122.41, 67.13, 33.78, 26.70 ppm.

**Synthesis of Compound SNT.** To a stirred solution of **SNT-b** in 2 mL DCM was added trifluoroacetic acid (74 mg, 0.54 mmol) and triisopropylsilane at room temperature. The mixture was stirred for 1 hour. The reaction was monitored by TLC (HEX/EA= 4:1, $R_f$ = 0.4). Upon reaction completion, toluene (5 mL) was added to the reaction mixture and the solvent was removed by rotary evaporation. The residue was purified by flash column chromatography (HEX/EA) to afford **SNT** as colorless oil in 77% yield (10.9 mg).
**$^1$H NMR** (400 MHz, $CDCl_3$) δ 8.28 (d, *J* = 9.3 Hz, 2H), 7.30 (d, *J* = 9.3 Hz, 2H), 2.98 – 2.94 (m, 2H), 2.93 – 2.89 (m, 2H), 1.73 (t, *J* = 8.24, 1.28 Hz, 1H) ppm.

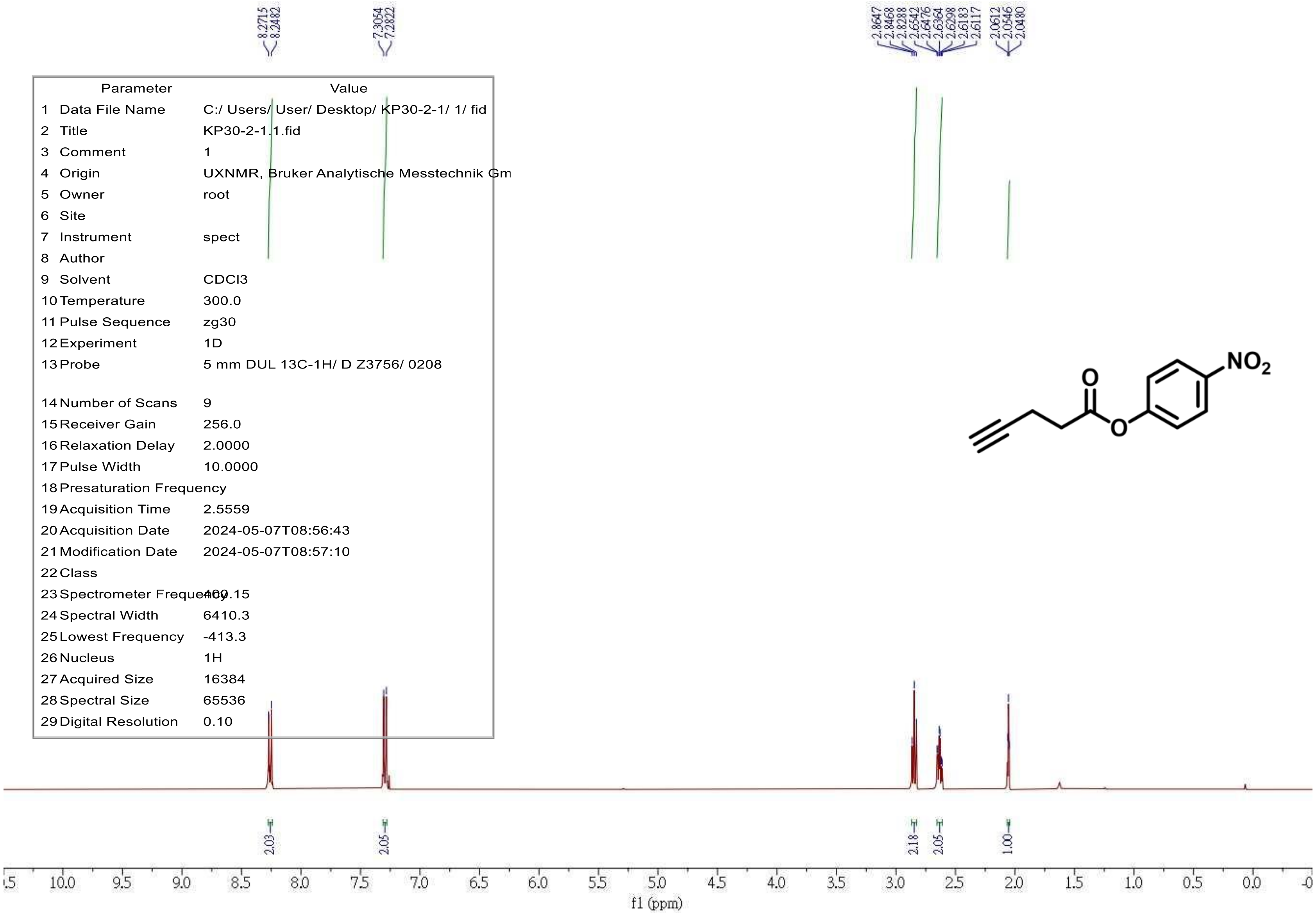
8.2715
8.2482
7.3054
7.2822
2.8647
2.8468
2.8288
2.6542
2.6476
2.6364
2.6298
2.6183
2.6117
2.0612
2.0546
2.0480
Parameter Value
1 Data File Name C:/ Users/ User/ Desktop/ KP30-2-1/ 1/ fid
2 Title KP30-2-1.1.fid
3 Comment 1
4 Origin UXNMR, Bruker Analytische Messtechnik Gm
5 Owner root
6 Site
7 Instrument spect
8 Author
9 Solvent CDCl3
10 Temperature 300.0
11 Pulse Sequence zg30
12 Experiment 1D
13 Probe 5 mm DUL 13C-1H/ D Z3756/ 0208
14 Number of Scans 9
15 Receiver Gain 256.0
16 Relaxation Delay 2.0000
17 Pulse Width 10.0000
18 Presaturation Frequency
19 Acquisition Time 2.5559
20 Acquisition Date 2024-05-07T08:56:43
21 Modification Date 2024-05-07T08:57:10
22 Class
23 Spectrometer Frequency 400.15
24 Spectral Width 6410.3
25 Lowest Frequency -413.3
26 Nucleus 1H
27 Acquired Size 16384
28 Spectral Size 65536
29 Digital Resolution 0.10
O
O
NO2
2.03
2.05
2.18
2.05
1.00
10.0 9.5 9.0 8.5 8.0 7.5 7.0 6.5 6.0 5.5 5.0 4.5 4.0 3.5 3.0 2.5 2.0 1.5 1.0 0.5 0.0
f1 (ppm)

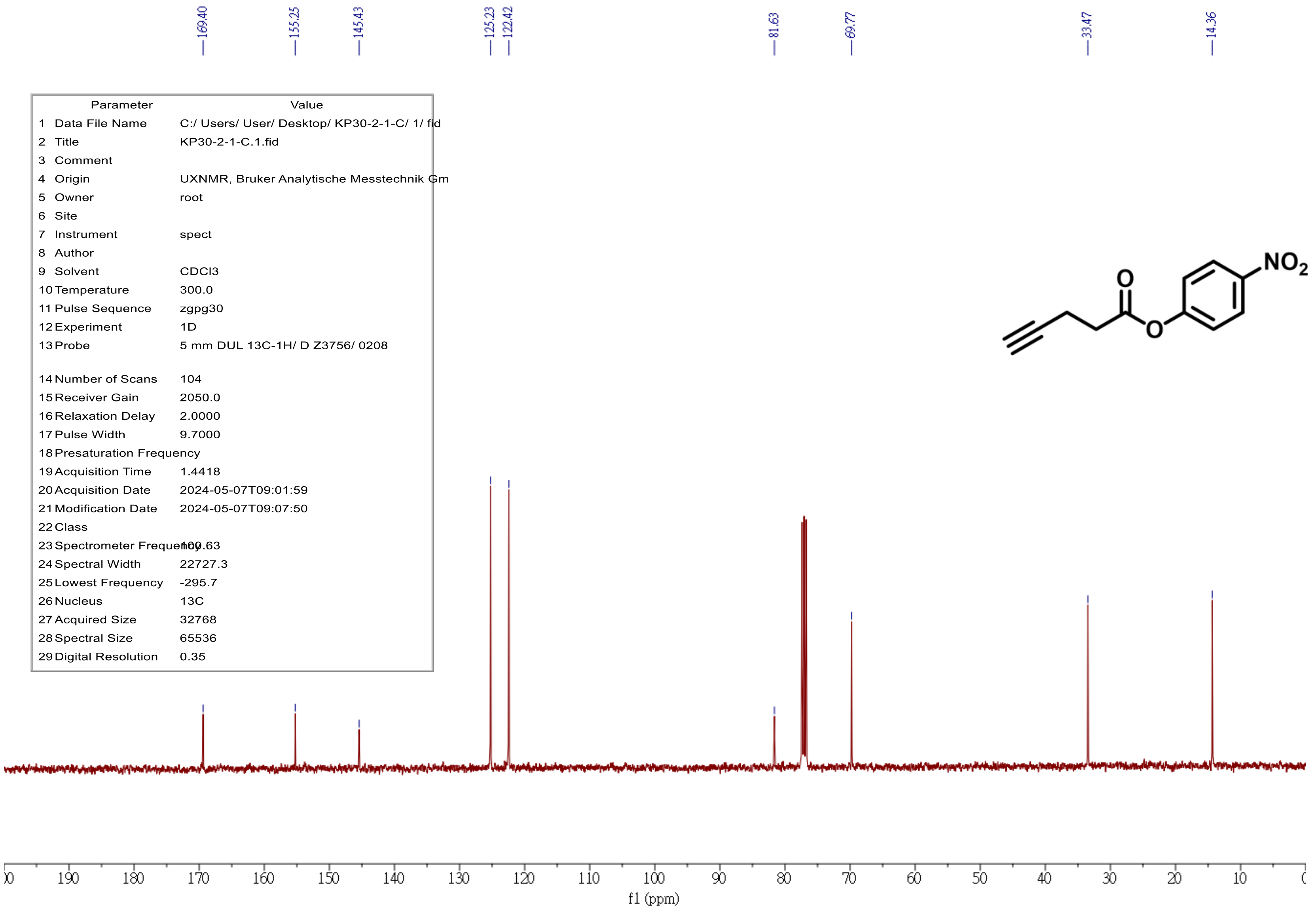


| | Parameter | Value |
|---|---|---|
| 1 | Data File Name | C:/ Users/ User/ Desktop/ KP30-2-1-C/ 1/ fid |
| 2 | Title | KP30-2-1-C.1.fid |
| 3 | Comment | |
| 4 | Origin | UXNMR, Bruker Analytische Messtechnik Gm |
| 5 | Owner | root |
| 6 | Site | |
| 7 | Instrument | spect |
| 8 | Author | |
| 9 | Solvent | CDCl3 |
| 10 | Temperature | 300.0 |
| 11 | Pulse Sequence | zgpg30 |
| 12 | Experiment | 1D |
| 13 | Probe | 5 mm DUL 13C-1H/ D Z3756/ 0208 |
| 14 | Number of Scans | 104 |
| 15 | Receiver Gain | 2050.0 |
| 16 | Relaxation Delay | 2.0000 |
| 17 | Pulse Width | 9.7000 |
| 18 | Presaturation Frequency | |
| 19 | Acquisition Time | 1.4418 |
| 20 | Acquisition Date | 2024-05-07T09:01:59 |
| 21 | Modification Date | 2024-05-07T09:07:50 |
| 22 | Class | |
| 23 | Spectrometer Frequency | 100.63 |
| 24 | Spectral Width | 22727.3 |
| 25 | Lowest Frequency | -295.7 |
| 26 | Nucleus | 13C |
| 27 | Acquired Size | 32768 |
| 28 | Spectral Size | 65536 |
| 29 | Digital Resolution | 0.35 |

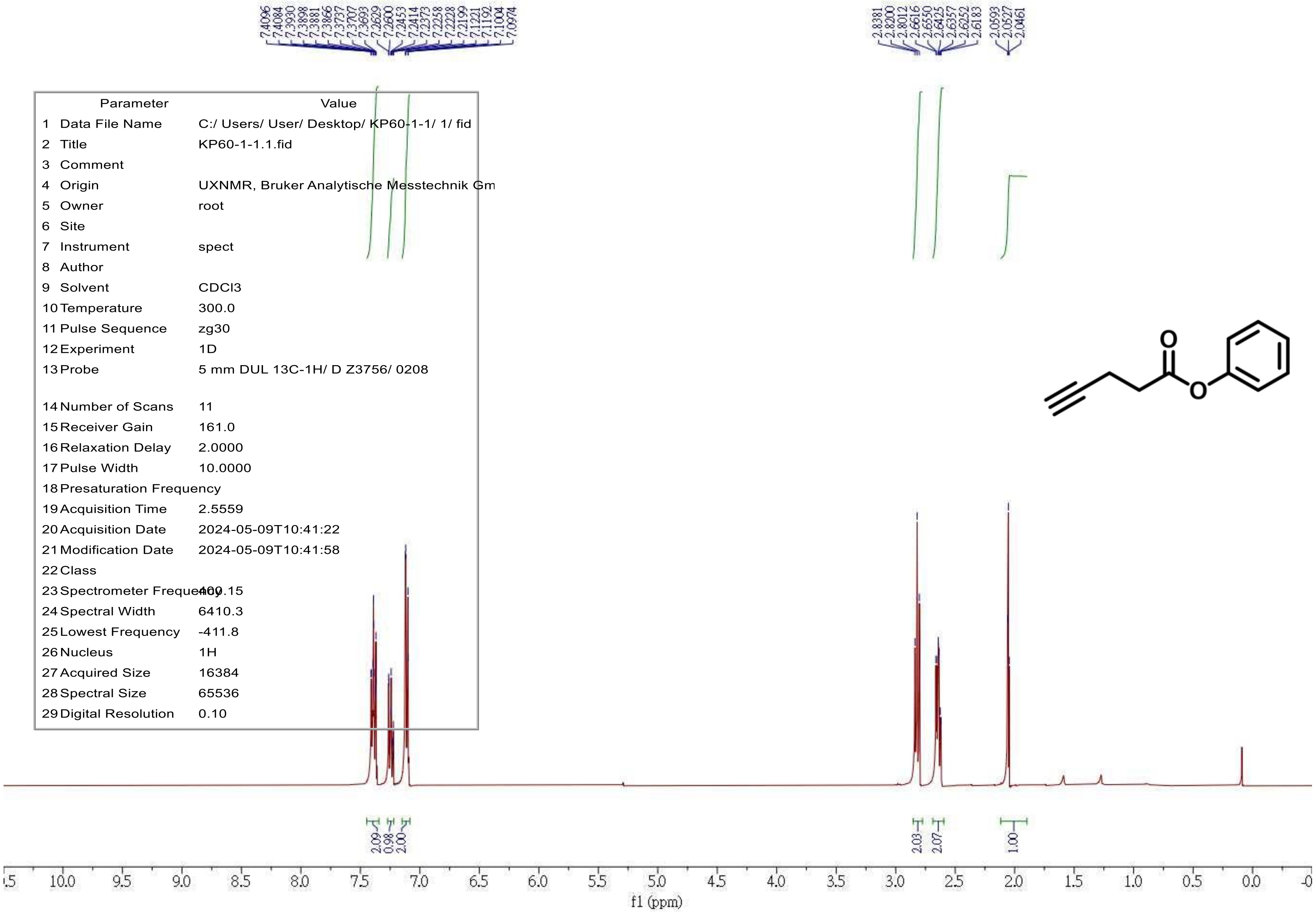


| | Parameter | Value |
|---|---|---|
| 1 | Data File Name | C:/ Users/ User/ Desktop/ KP60-1-1/ 1/ fid |
| 2 | Title | KP60-1-1.1.fid |
| 3 | Comment | |
| 4 | Origin | UXNMR, Bruker Analytische Messtechnik Gm |
| 5 | Owner | root |
| 6 | Site | |
| 7 | Instrument | spect |
| 8 | Author | |
| 9 | Solvent | CDCl3 |
| 10 | Temperature | 300.0 |
| 11 | Pulse Sequence | zg30 |
| 12 | Experiment | 1D |
| 13 | Probe | 5 mm DUL 13C-1H/ D Z3756/ 0208 |
| 14 | Number of Scans | 11 |
| 15 | Receiver Gain | 161.0 |
| 16 | Relaxation Delay | 2.0000 |
| 17 | Pulse Width | 10.0000 |
| 18 | Presaturation Frequency | |
| 19 | Acquisition Time | 2.5559 |
| 20 | Acquisition Date | 2024-05-09T10:41:22 |
| 21 | Modification Date | 2024-05-09T10:41:58 |
| 22 | Class | |
| 23 | Spectrometer Frequency | 400.15 |
| 24 | Spectral Width | 6410.3 |
| 25 | Lowest Frequency | -411.8 |
| 26 | Nucleus | 1H |
| 27 | Acquired Size | 16384 |
| 28 | Spectral Size | 65536 |
| 29 | Digital Resolution | 0.10 |

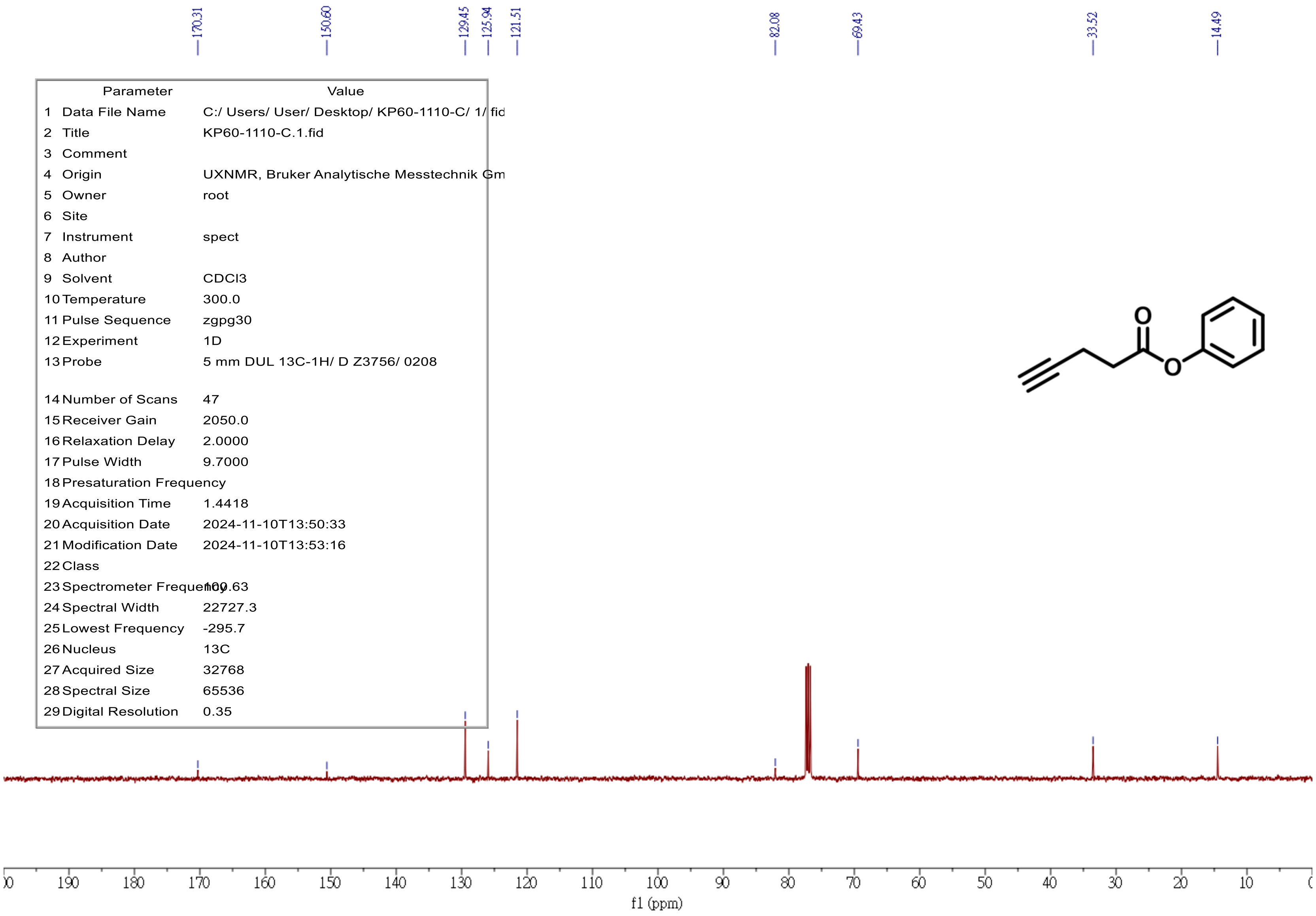


| | Parameter | Value |
|---|---|---|
| 1 | Data File Name | C:/ Users/ User/ Desktop/ KP60-1110-C/ 1/ fid |
| 2 | Title | KP60-1110-C.1.fid |
| 3 | Comment | |
| 4 | Origin | UXNMR, Bruker Analytische Messtechnik Gm |
| 5 | Owner | root |
| 6 | Site | |
| 7 | Instrument | spect |
| 8 | Author | |
| 9 | Solvent | CDCl3 |
| 10 | Temperature | 300.0 |
| 11 | Pulse Sequence | zgpg30 |
| 12 | Experiment | 1D |
| 13 | Probe | 5 mm DUL 13C-1H/ D Z3756/ 0208 |
| 14 | Number of Scans | 47 |
| 15 | Receiver Gain | 2050.0 |
| 16 | Relaxation Delay | 2.0000 |
| 17 | Pulse Width | 9.7000 |
| 18 | Presaturation Frequency | |
| 19 | Acquisition Time | 1.4418 |
| 20 | Acquisition Date | 2024-11-10T13:50:33 |
| 21 | Modification Date | 2024-11-10T13:53:16 |
| 22 | Class | |
| 23 | Spectrometer Frequency | 100.63 |
| 24 | Spectral Width | 22727.3 |
| 25 | Lowest Frequency | -295.7 |
| 26 | Nucleus | 13C |
| 27 | Acquired Size | 32768 |
| 28 | Spectral Size | 65536 |
| 29 | Digital Resolution | 0.35 |

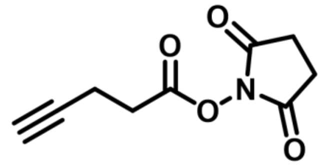

| Parameter | Value |
|---|---|
| 1 Data File Name | Y:/ 個人資料夾/ 趙翊丞/ CY122-2/ 1/ fid |
| 2 Title | CY122-2.1.fid |
| 3 Comment | CY122-2 |
| 4 Origin | UXNMR, Bruker Analytische Messtechnik Gm |
| 5 Owner | root |
| 6 Site | |
| 7 Instrument | spect |
| 8 Author | |
| 9 Solvent | CDCl3 |
| 10 Temperature | 300.0 |
| 11 Pulse Sequence | zg30 |
| 12 Experiment | 1D |
| 13 Probe | 5 mm DUL 13C-1H/ D Z3756/ 0208 |
| 14 Number of Scans | 6 |
| 15 Receiver Gain | 203.0 |
| 16 Relaxation Delay | 2.0000 |
| 17 Pulse Width | 10.0000 |
| 18 Presaturation Frequency | |
| 19 Acquisition Time | 2.5559 |
| 20 Acquisition Date | 2023-09-21T11:35:34 |
| 21 Modification Date | 2023-09-21T11:35:58 |
| 22 Class | |
| 23 Spectrometer Frequency | 400.15 |
| 24 Spectral Width | 6410.3 |
| 25 Lowest Frequency | -413.8 |
| 26 Nucleus | 1H |
| 27 Acquired Size | 16384 |
| 28 Spectral Size | 65536 |
| 29 Digital Resolution | 0.10 |

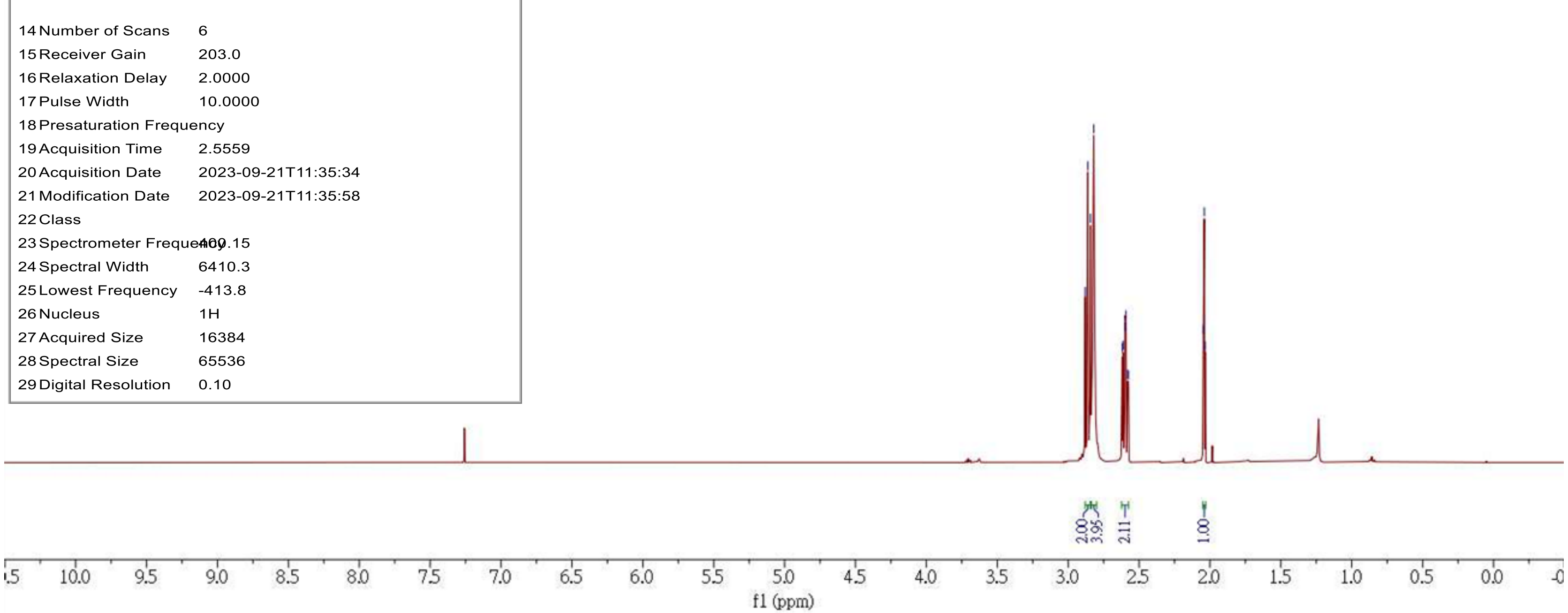

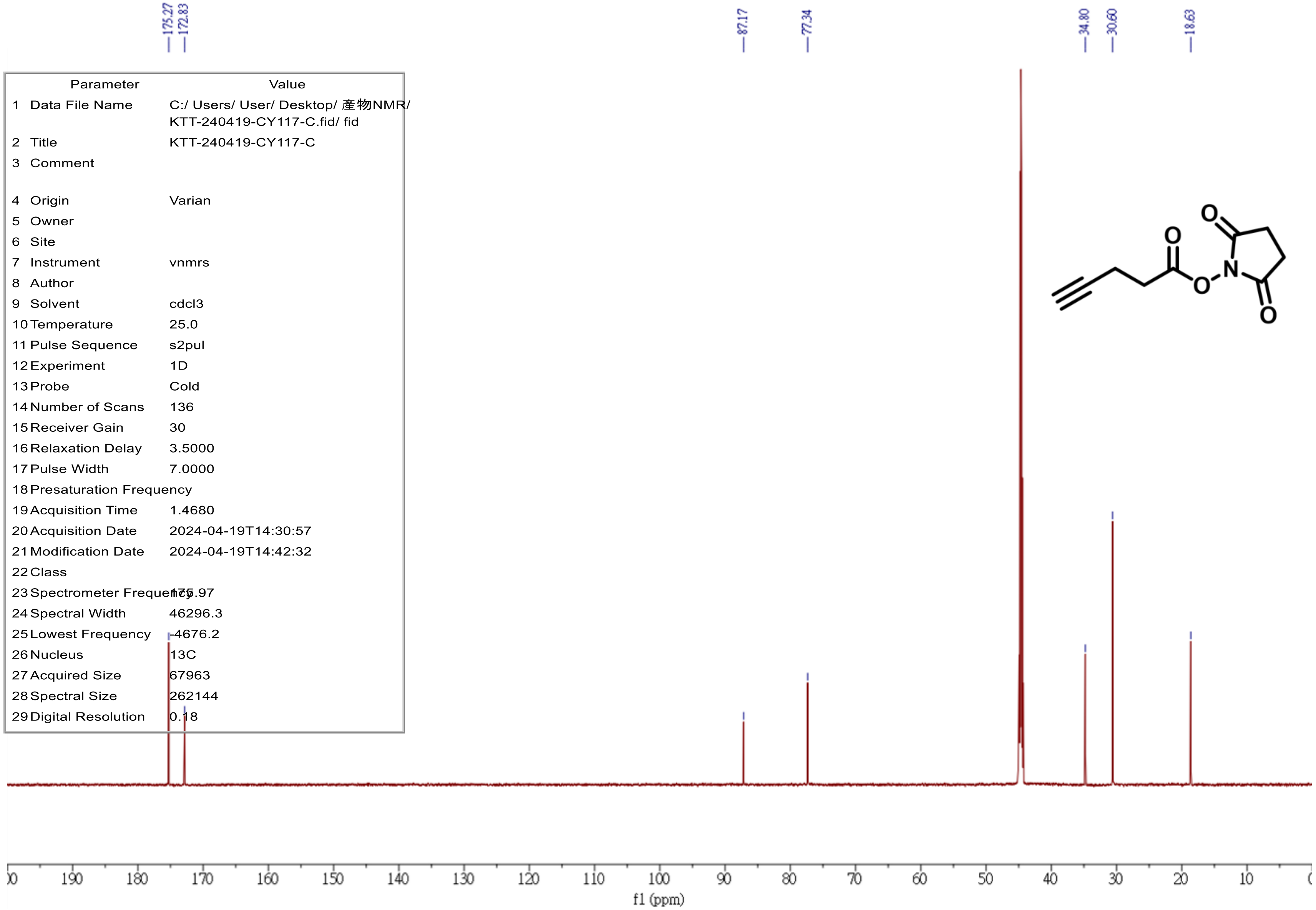
175.27
172.83
87.17
77.34
34.80
30.60
18.63
Parameter | Value
1 Data File Name | C:/ Users/ User/ Desktop/ 產物NMR/ KTT-240419-CY117-C.fid/ fid
2 Title | KTT-240419-CY117-C
3 Comment
4 Origin | Varian
5 Owner
6 Site
7 Instrument | vnmrs
8 Author
9 Solvent | cdcl3
10 Temperature | 25.0
11 Pulse Sequence | s2pul
12 Experiment | 1D
13 Probe | Cold
14 Number of Scans | 136
15 Receiver Gain | 30
16 Relaxation Delay | 3.5000
17 Pulse Width | 7.0000
18 Presaturation Frequency
19 Acquisition Time | 1.4680
20 Acquisition Date | 2024-04-19T14:30:57
21 Modification Date | 2024-04-19T14:42:32
22 Class
23 Spectrometer Frequency | 175.97
24 Spectral Width | 46296.3
25 Lowest Frequency | -4676.2
26 Nucleus | 13C
27 Acquired Size | 67963
28 Spectral Size | 262144
29 Digital Resolution | 0.18
O
O
O
N
O
O
190
180
170
160
150
140
130
120
110
100
90
80
70
60
50
40
30
20
10
f1 (ppm)

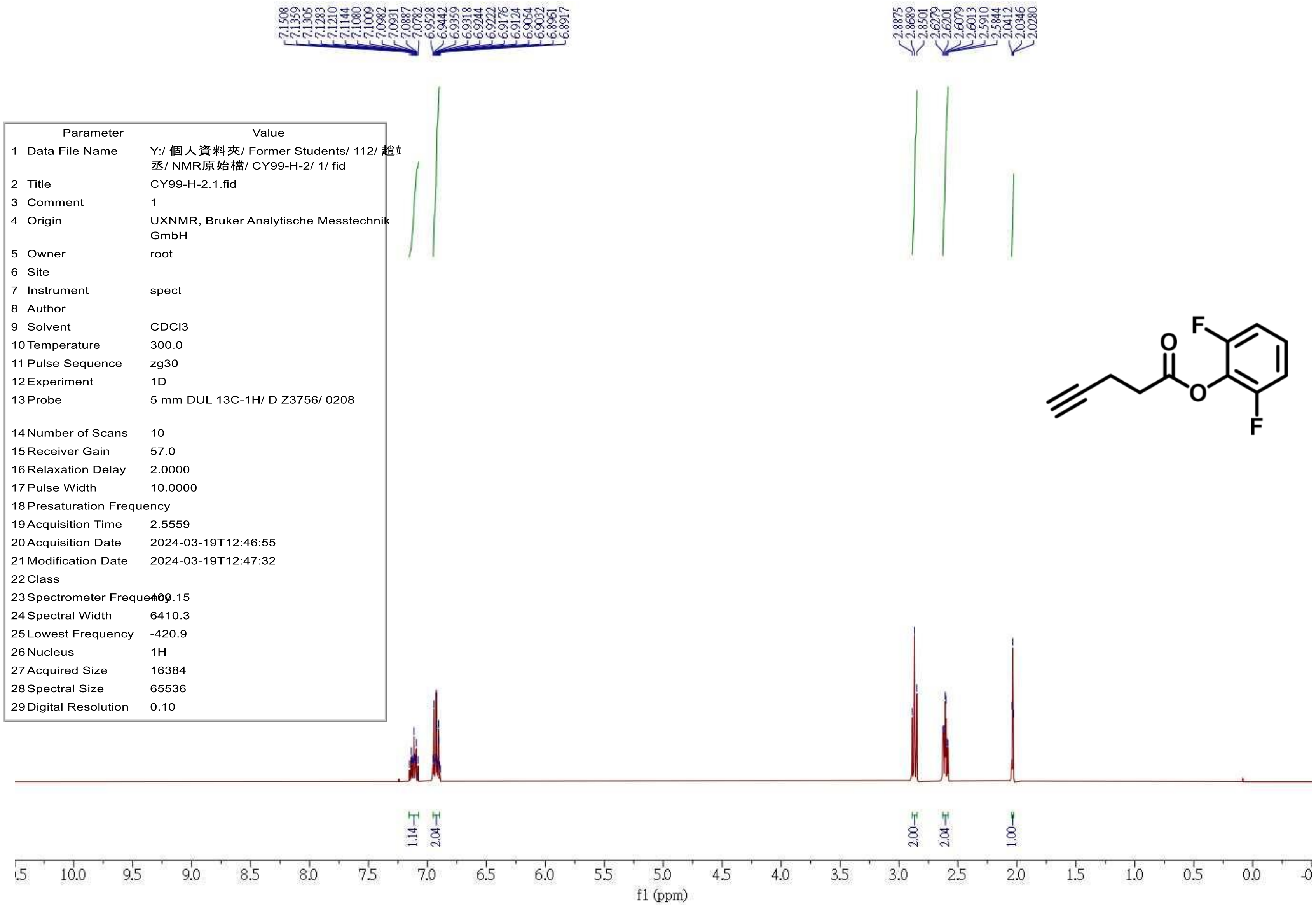


| Parameter | | Value |
|---|---|---|
| 1 | Data File Name | Y:/ 個人資料夾/ Former Students/ 112/ 趙[illegible] 丞/ NMR原始檔/ CY99-H-2/ 1/ fid |
| 2 | Title | CY99-H-2.1.fid |
| 3 | Comment | 1 |
| 4 | Origin | UXNMR, Bruker Analytische Messtechnik GmbH |
| 5 | Owner | root |
| 6 | Site | |
| 7 | Instrument | spect |
| 8 | Author | |
| 9 | Solvent | CDCl3 |
| 10 | Temperature | 300.0 |
| 11 | Pulse Sequence | zg30 |
| 12 | Experiment | 1D |
| 13 | Probe | 5 mm DUL 13C-1H/ D Z3756/ 0208 |
| 14 | Number of Scans | 10 |
| 15 | Receiver Gain | 57.0 |
| 16 | Relaxation Delay | 2.0000 |
| 17 | Pulse Width | 10.0000 |
| 18 | Presaturation Frequency | |
| 19 | Acquisition Time | 2.5559 |
| 20 | Acquisition Date | 2024-03-19T12:46:55 |
| 21 | Modification Date | 2024-03-19T12:47:32 |
| 22 | Class | |
| 23 | Spectrometer Frequency | 400.15 |
| 24 | Spectral Width | 6410.3 |
| 25 | Lowest Frequency | -420.9 |
| 26 | Nucleus | 1H |
| 27 | Acquired Size | 16384 |
| 28 | Spectral Size | 65536 |
| 29 | Digital Resolution | 0.10 |

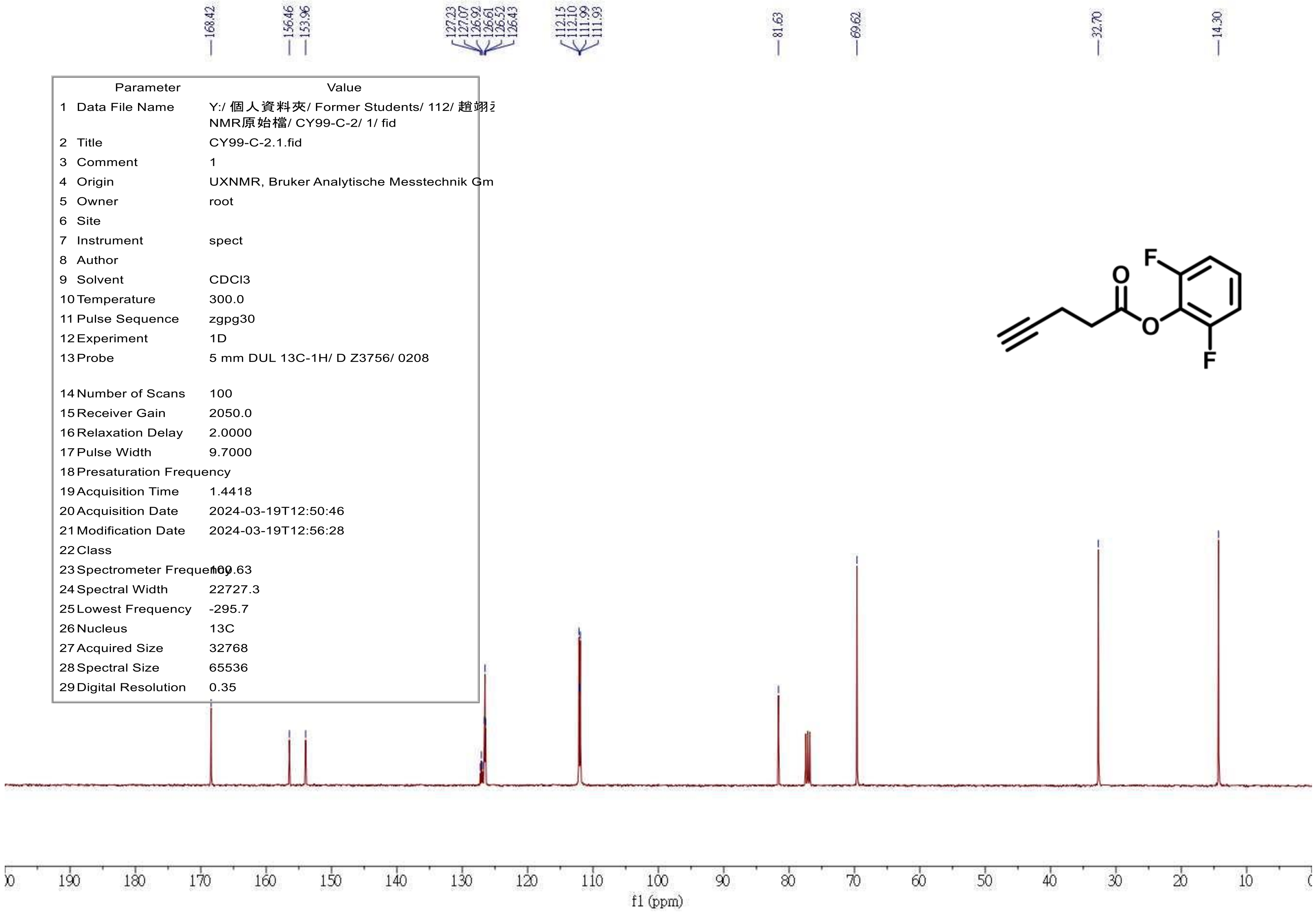


| | Parameter | Value |
|---|---|---|
| 1 | Data File Name | Y:/ 個人資料夾/ Former Students/ 112/ 趙翊 NMR原始檔/ CY99-C-2/ 1/ fid |
| 2 | Title | CY99-C-2.1.fid |
| 3 | Comment | 1 |
| 4 | Origin | UXNMR, Bruker Analytische Messtechnik Gm |
| 5 | Owner | root |
| 6 | Site | |
| 7 | Instrument | spect |
| 8 | Author | |
| 9 | Solvent | CDCl3 |
| 10 | Temperature | 300.0 |
| 11 | Pulse Sequence | zgpg30 |
| 12 | Experiment | 1D |
| 13 | Probe | 5 mm DUL 13C-1H/ D Z3756/ 0208 |
| 14 | Number of Scans | 100 |
| 15 | Receiver Gain | 2050.0 |
| 16 | Relaxation Delay | 2.0000 |
| 17 | Pulse Width | 9.7000 |
| 18 | Presaturation Frequency | |
| 19 | Acquisition Time | 1.4418 |
| 20 | Acquisition Date | 2024-03-19T12:50:46 |
| 21 | Modification Date | 2024-03-19T12:56:28 |
| 22 | Class | |
| 23 | Spectrometer Frequency | 100.63 |
| 24 | Spectral Width | 22727.3 |
| 25 | Lowest Frequency | -295.7 |
| 26 | Nucleus | 13C |
| 27 | Acquired Size | 32768 |
| 28 | Spectral Size | 65536 |
| 29 | Digital Resolution | 0.35 |

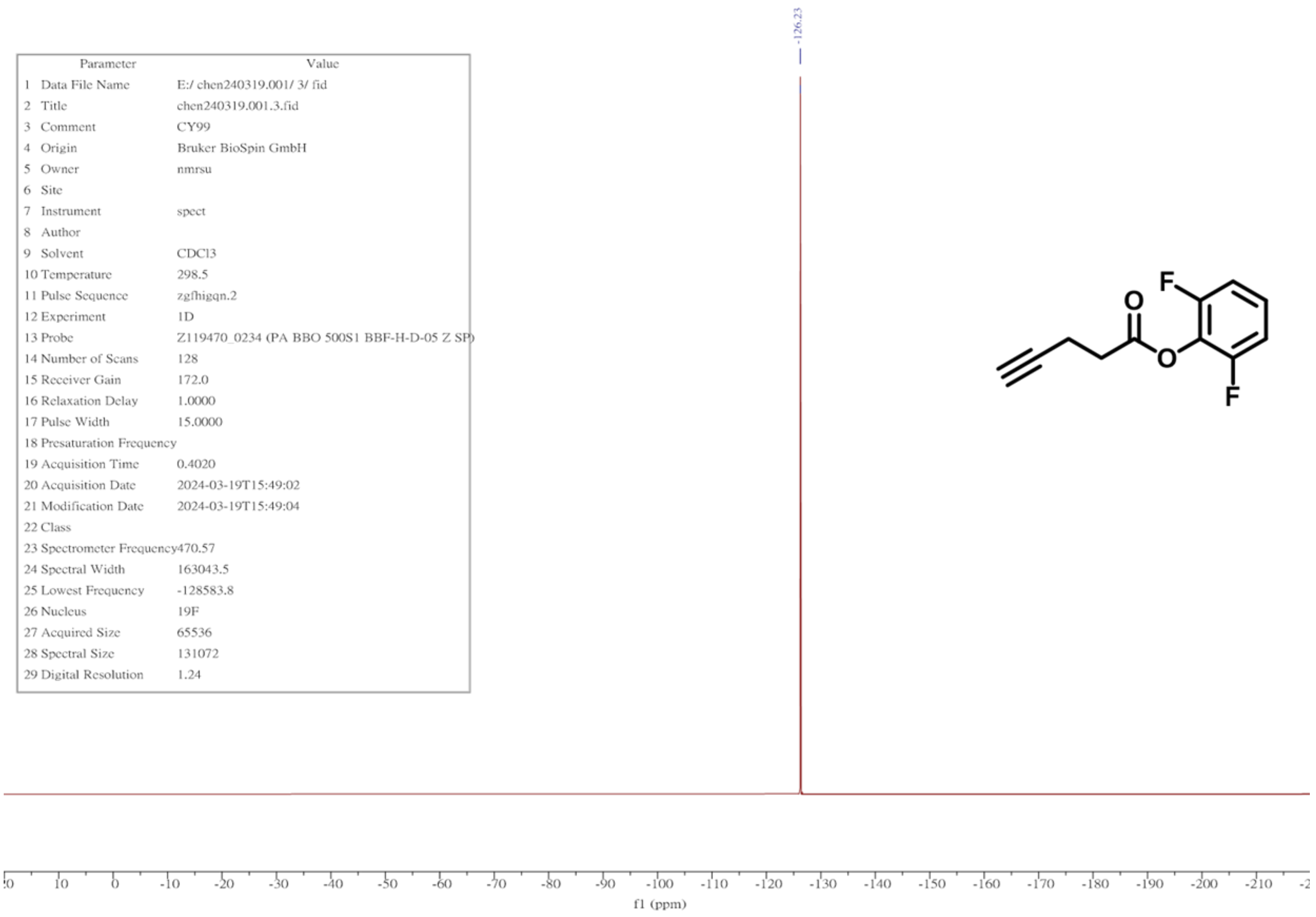


| | Parameter | Value |
|---|---|---|
| 1 | Data File Name | E:/ chen240319.001/ 3/ fid |
| 2 | Title | chen240319.001.3.fid |
| 3 | Comment | CY99 |
| 4 | Origin | Bruker BioSpin GmbH |
| 5 | Owner | nmrsu |
| 6 | Site | |
| 7 | Instrument | spect |
| 8 | Author | |
| 9 | Solvent | CDCl3 |
| 10 | Temperature | 298.5 |
| 11 | Pulse Sequence | zgfhigqn.2 |
| 12 | Experiment | 1D |
| 13 | Probe | Z119470_0234 (PA BBO 500S1 BBF-H-D-05 Z SP) |
| 14 | Number of Scans | 128 |
| 15 | Receiver Gain | 172.0 |
| 16 | Relaxation Delay | 1.0000 |
| 17 | Pulse Width | 15.0000 |
| 18 | Presaturation Frequency | |
| 19 | Acquisition Time | 0.4020 |
| 20 | Acquisition Date | 2024-03-19T15:49:02 |
| 21 | Modification Date | 2024-03-19T15:49:04 |
| 22 | Class | |
| 23 | Spectrometer Frequency | 470.57 |
| 24 | Spectral Width | 163043.5 |
| 25 | Lowest Frequency | -128583.8 |
| 26 | Nucleus | 19F |
| 27 | Acquired Size | 65536 |
| 28 | Spectral Size | 131072 |
| 29 | Digital Resolution | 1.24 |

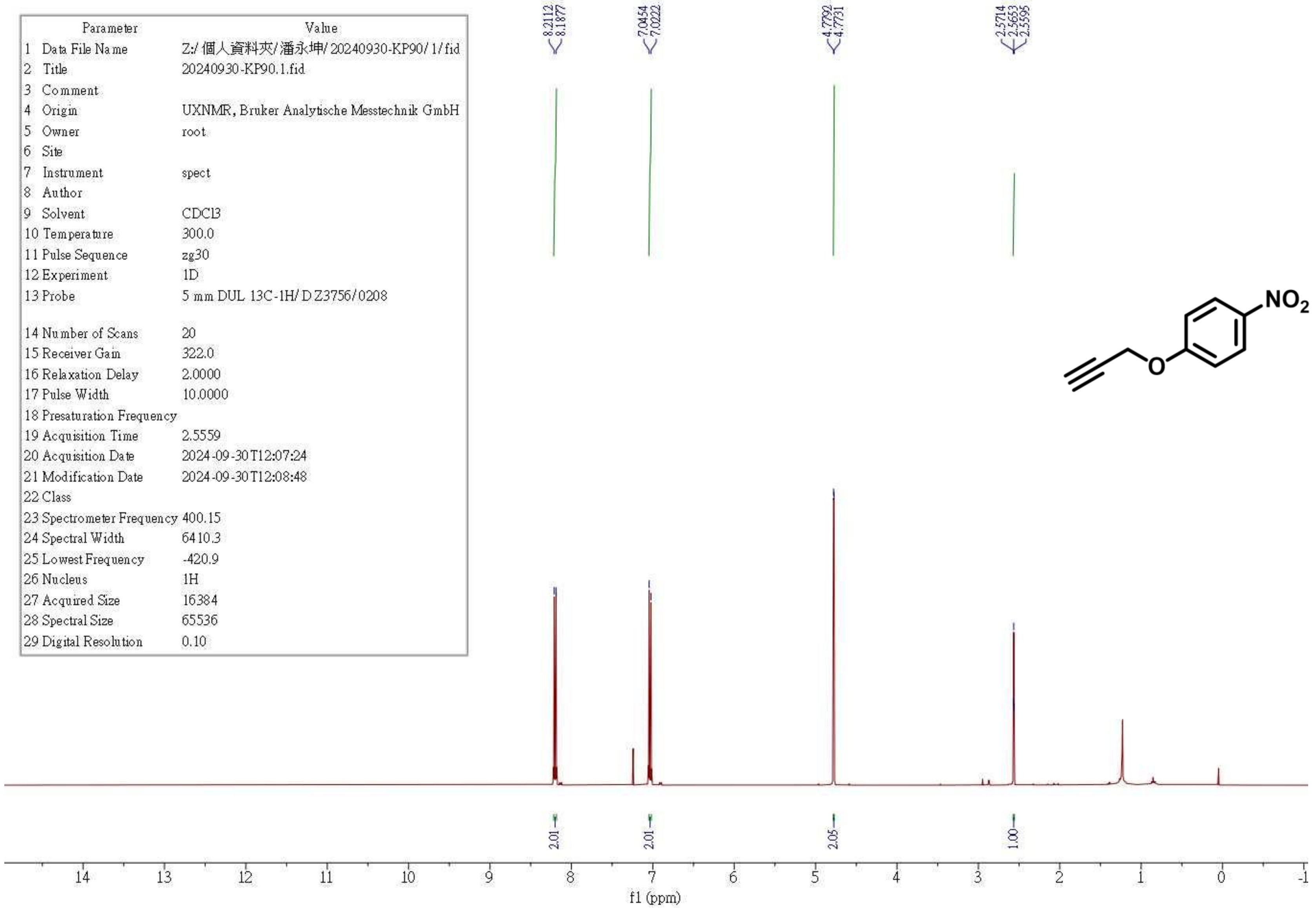


| | Parameter | Value |
|---|---|---|
| 1 | Data File Name | Z:/ 個人資料夾/ 潘永坤/ 20240930-KP90/ 1/ fid |
| 2 | Title | 20240930-KP90.1.fid |
| 3 | Comment | |
| 4 | Origin | UXNMR, Bruker Analytische Messtechnik GmbH |
| 5 | Owner | root |
| 6 | Site | |
| 7 | Instrument | spect |
| 8 | Author | |
| 9 | Solvent | CDCl3 |
| 10 | Temperature | 300.0 |
| 11 | Pulse Sequence | zg30 |
| 12 | Experiment | 1D |
| 13 | Probe | 5 mm DUL 13C-1H/ D Z3756/ 0208 |
| 14 | Number of Scans | 20 |
| 15 | Receiver Gain | 322.0 |
| 16 | Relaxation Delay | 2.0000 |
| 17 | Pulse Width | 10.0000 |
| 18 | Presaturation Frequency | |
| 19 | Acquisition Time | 2.5559 |
| 20 | Acquisition Date | 2024-09-30T12:07:24 |
| 21 | Modification Date | 2024-09-30T12:08:48 |
| 22 | Class | |
| 23 | Spectrometer Frequency | 400.15 |
| 24 | Spectral Width | 6410.3 |
| 25 | Lowest Frequency | -420.9 |
| 26 | Nucleus | 1H |
| 27 | Acquired Size | 16384 |
| 28 | Spectral Size | 65536 |
| 29 | Digital Resolution | 0.10 |

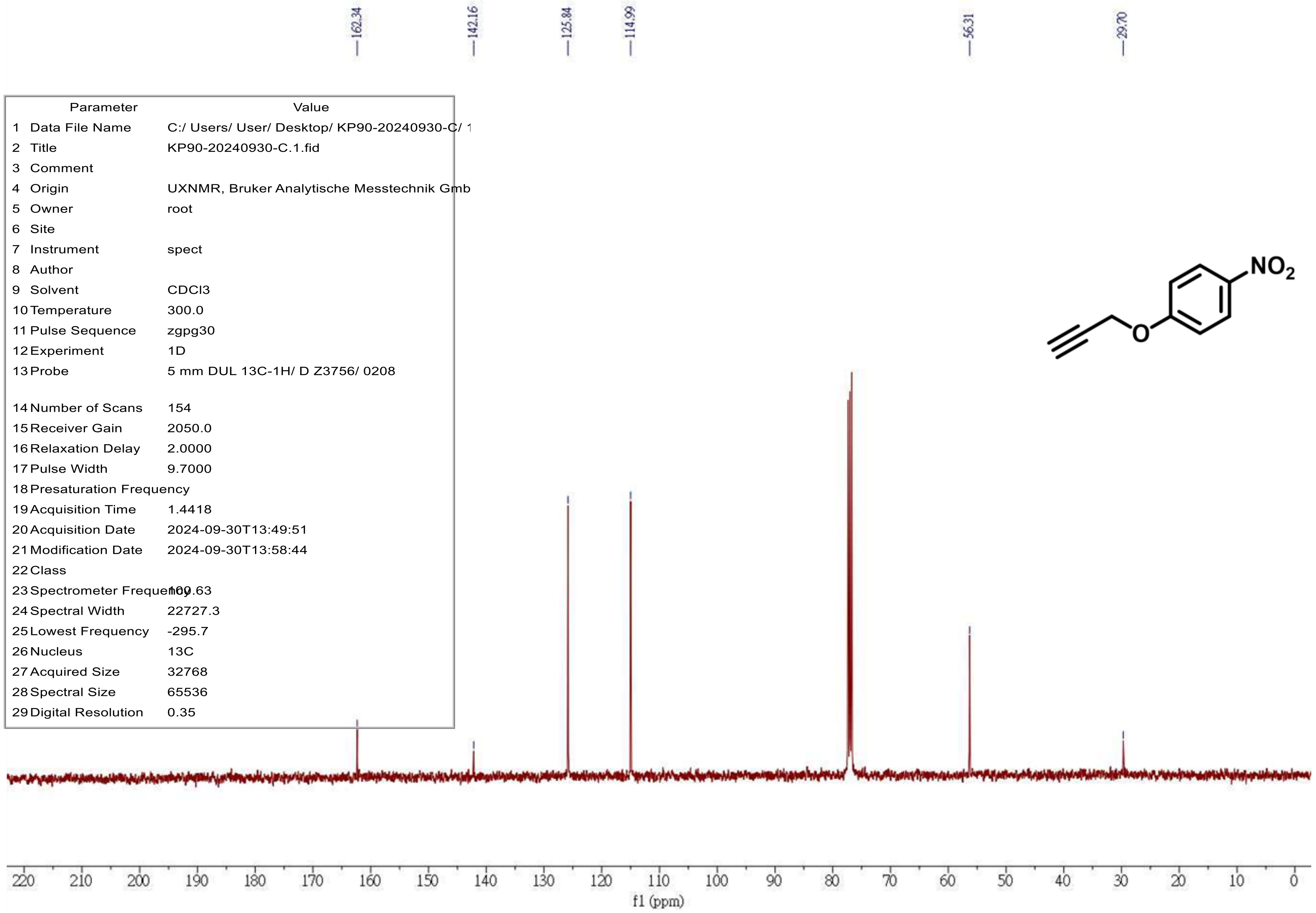


| | Parameter | Value |
|---|---|---|
| 1 | Data File Name | C:/ Users/ User/ Desktop/ KP90-20240930-C/ 1 |
| 2 | Title | KP90-20240930-C.1.fid |
| 3 | Comment | |
| 4 | Origin | UXNMR, Bruker Analytische Messtechnik Gmb |
| 5 | Owner | root |
| 6 | Site | |
| 7 | Instrument | spect |
| 8 | Author | |
| 9 | Solvent | CDCl3 |
| 10 | Temperature | 300.0 |
| 11 | Pulse Sequence | zgpg30 |
| 12 | Experiment | 1D |
| 13 | Probe | 5 mm DUL 13C-1H/ D Z3756/ 0208 |
| 14 | Number of Scans | 154 |
| 15 | Receiver Gain | 2050.0 |
| 16 | Relaxation Delay | 2.0000 |
| 17 | Pulse Width | 9.7000 |
| 18 | Presaturation Frequency | |
| 19 | Acquisition Time | 1.4418 |
| 20 | Acquisition Date | 2024-09-30T13:49:51 |
| 21 | Modification Date | 2024-09-30T13:58:44 |
| 22 | Class | |
| 23 | Spectrometer Frequency | 100.63 |
| 24 | Spectral Width | 22727.3 |
| 25 | Lowest Frequency | -295.7 |
| 26 | Nucleus | 13C |
| 27 | Acquired Size | 32768 |
| 28 | Spectral Size | 65536 |
| 29 | Digital Resolution | 0.35 |

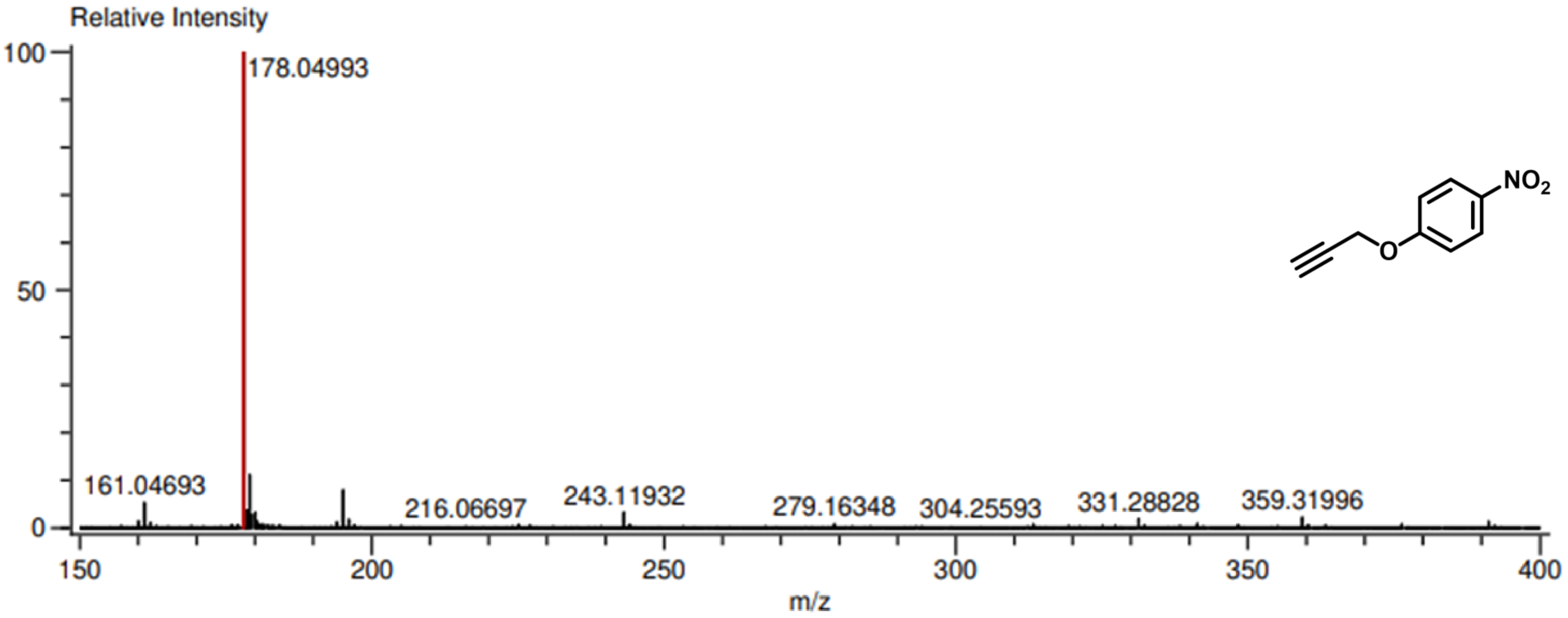


| Mass | Intensity | Calc. Mass | Mass Difference [mDa] | Mass Difference [ppm] | Possible Formula |
|---|---|---|---|---|---|
| 178.04993 | 137073.01 | 178.05042 | -0.49 | -2.74 | $^{12}C_9{}^{1}H_8{}^{14}N_1{}^{16}O_3$ |

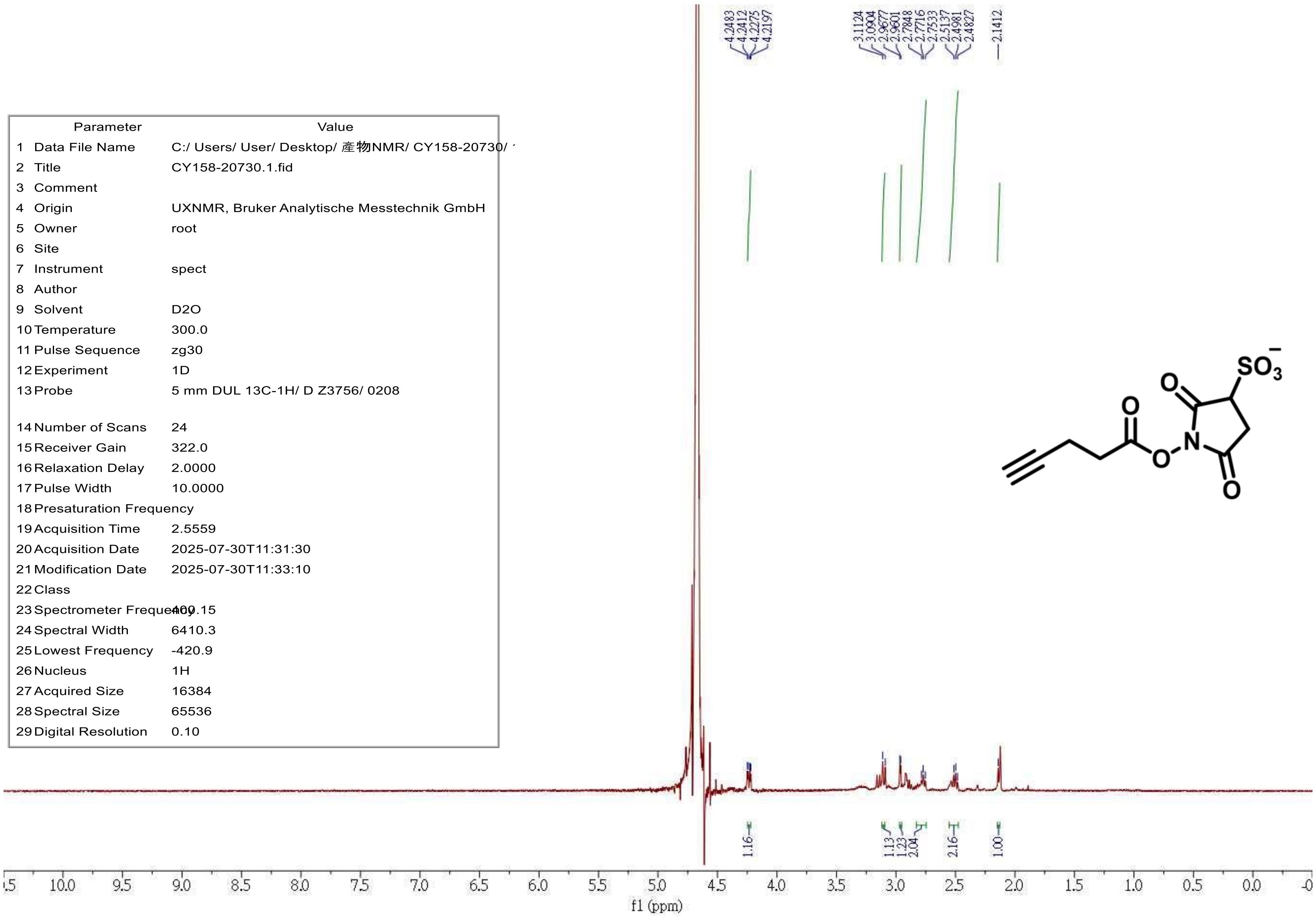


| | Parameter | Value |
|---|---|---|
| 1 | Data File Name | C:/ Users/ User/ Desktop/ 產物NMR/ CY158-20730/ ` |
| 2 | Title | CY158-20730.1.fid |
| 3 | Comment | |
| 4 | Origin | UXNMR, Bruker Analytische Messtechnik GmbH |
| 5 | Owner | root |
| 6 | Site | |
| 7 | Instrument | spect |
| 8 | Author | |
| 9 | Solvent | D2O |
| 10 | Temperature | 300.0 |
| 11 | Pulse Sequence | zg30 |
| 12 | Experiment | 1D |
| 13 | Probe | 5 mm DUL 13C-1H/ D Z3756/ 0208 |
| 14 | Number of Scans | 24 |
| 15 | Receiver Gain | 322.0 |
| 16 | Relaxation Delay | 2.0000 |
| 17 | Pulse Width | 10.0000 |
| 18 | Presaturation Frequency | |
| 19 | Acquisition Time | 2.5559 |
| 20 | Acquisition Date | 2025-07-30T11:31:30 |
| 21 | Modification Date | 2025-07-30T11:33:10 |
| 22 | Class | |
| 23 | Spectrometer Frequency | 400.15 |
| 24 | Spectral Width | 6410.3 |
| 25 | Lowest Frequency | -420.9 |
| 26 | Nucleus | 1H |
| 27 | Acquired Size | 16384 |
| 28 | Spectral Size | 65536 |
| 29 | Digital Resolution | 0.10 |

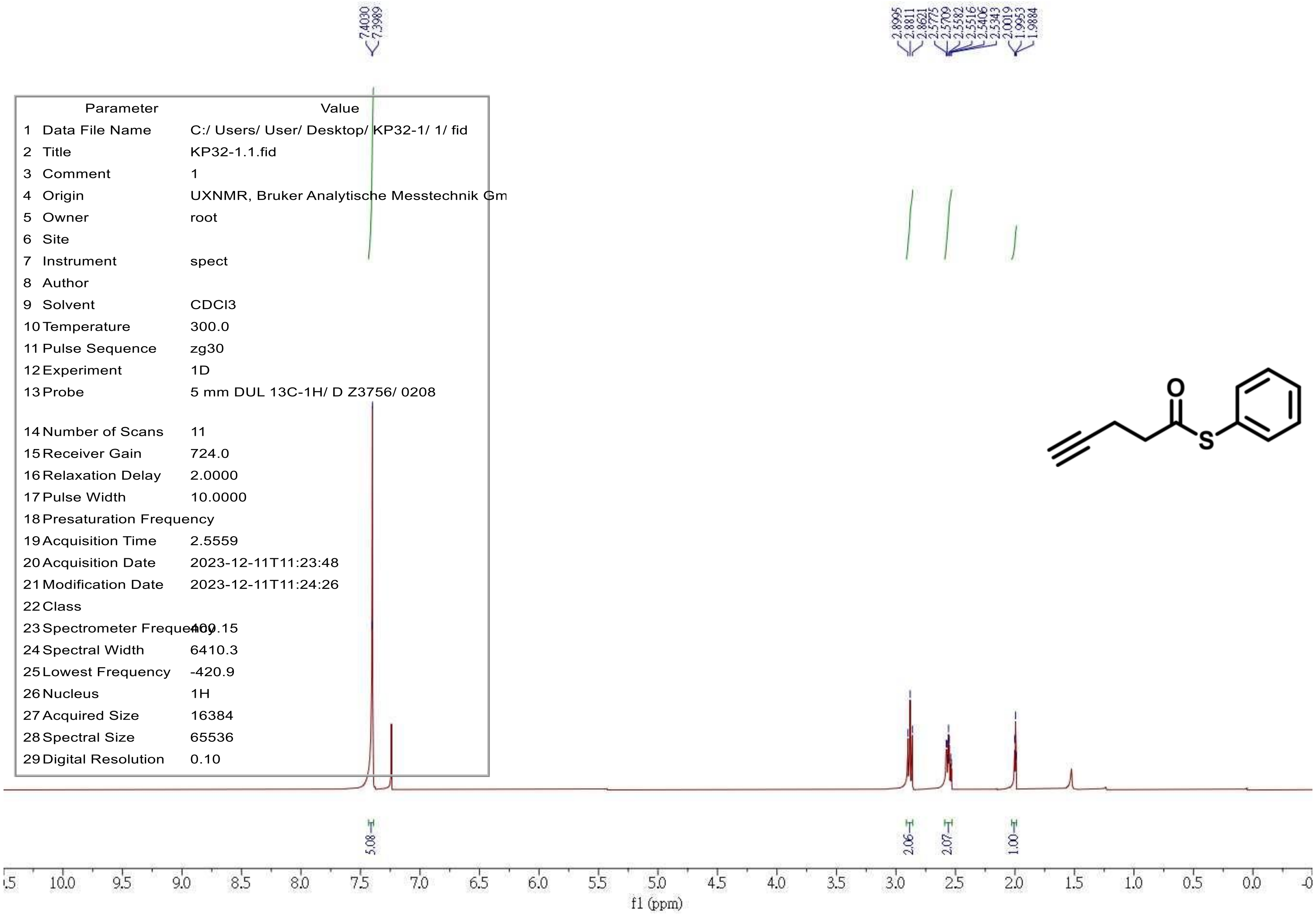


| | Parameter | Value |
|---|---|---|
| 1 | Data File Name | C:/ Users/ User/ Desktop/ KP32-1/ 1/ fid |
| 2 | Title | KP32-1.1.fid |
| 3 | Comment | 1 |
| 4 | Origin | UXNMR, Bruker Analytische Messtechnik Gm |
| 5 | Owner | root |
| 6 | Site | |
| 7 | Instrument | spect |
| 8 | Author | |
| 9 | Solvent | CDCl3 |
| 10 | Temperature | 300.0 |
| 11 | Pulse Sequence | zg30 |
| 12 | Experiment | 1D |
| 13 | Probe | 5 mm DUL 13C-1H/ D Z3756/ 0208 |
| 14 | Number of Scans | 11 |
| 15 | Receiver Gain | 724.0 |
| 16 | Relaxation Delay | 2.0000 |
| 17 | Pulse Width | 10.0000 |
| 18 | Presaturation Frequency | |
| 19 | Acquisition Time | 2.5559 |
| 20 | Acquisition Date | 2023-12-11T11:23:48 |
| 21 | Modification Date | 2023-12-11T11:24:26 |
| 22 | Class | |
| 23 | Spectrometer Frequency | 400.15 |
| 24 | Spectral Width | 6410.3 |
| 25 | Lowest Frequency | -420.9 |
| 26 | Nucleus | 1H |
| 27 | Acquired Size | 16384 |
| 28 | Spectral Size | 65536 |
| 29 | Digital Resolution | 0.10 |

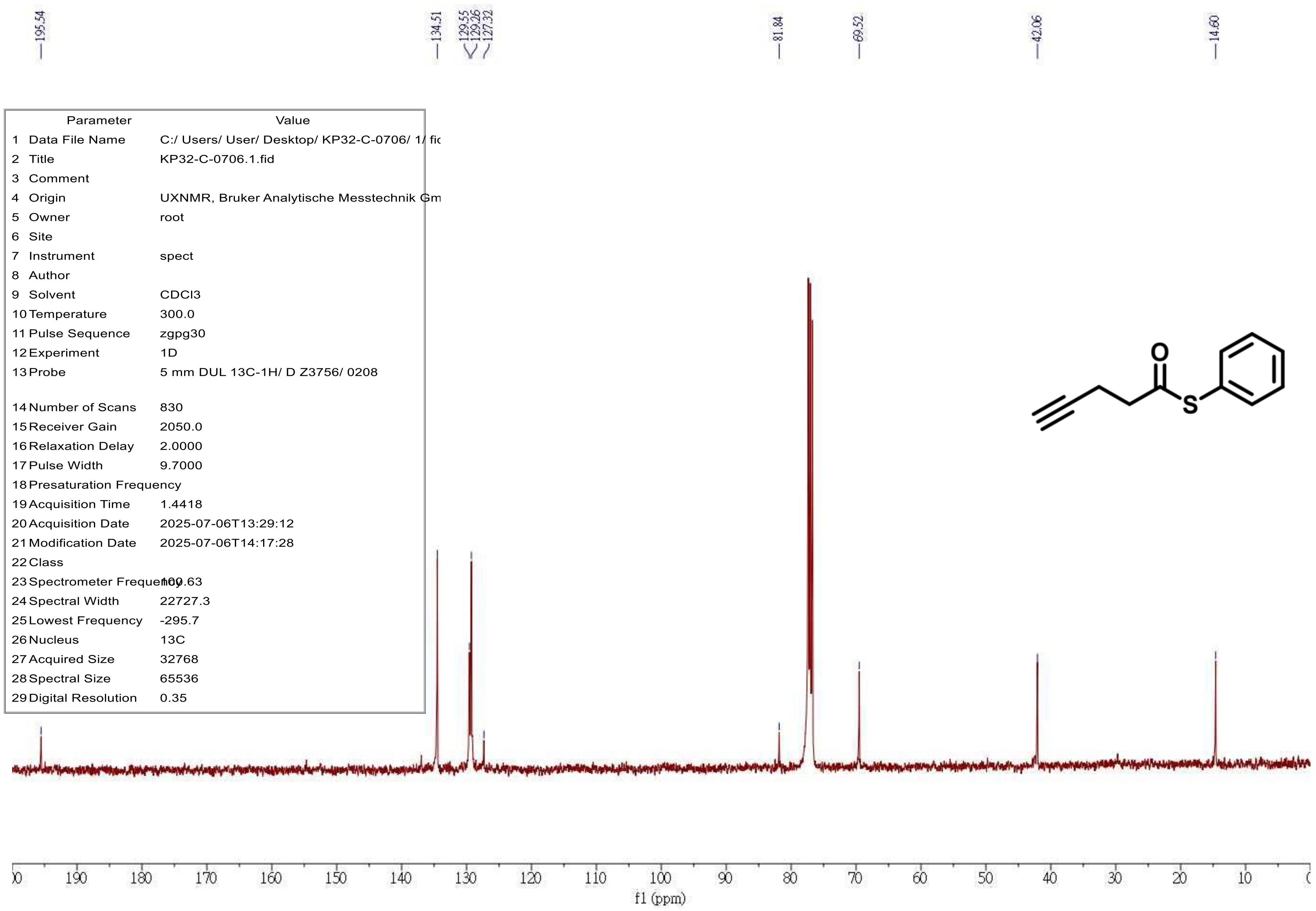


| | Parameter | Value |
|---|---|---|
| 1 | Data File Name | C:/ Users/ User/ Desktop/ KP32-C-0706/ 1/ fic |
| 2 | Title | KP32-C-0706.1.fid |
| 3 | Comment | |
| 4 | Origin | UXNMR, Bruker Analytische Messtechnik Gm |
| 5 | Owner | root |
| 6 | Site | |
| 7 | Instrument | spect |
| 8 | Author | |
| 9 | Solvent | CDCl3 |
| 10 | Temperature | 300.0 |
| 11 | Pulse Sequence | zgpg30 |
| 12 | Experiment | 1D |
| 13 | Probe | 5 mm DUL 13C-1H/ D Z3756/ 0208 |
| 14 | Number of Scans | 830 |
| 15 | Receiver Gain | 2050.0 |
| 16 | Relaxation Delay | 2.0000 |
| 17 | Pulse Width | 9.7000 |
| 18 | Presaturation Frequency | |
| 19 | Acquisition Time | 1.4418 |
| 20 | Acquisition Date | 2025-07-06T13:29:12 |
| 21 | Modification Date | 2025-07-06T14:17:28 |
| 22 | Class | |
| 23 | Spectrometer Frequency | 100.63 |
| 24 | Spectral Width | 22727.3 |
| 25 | Lowest Frequency | -295.7 |
| 26 | Nucleus | 13C |
| 27 | Acquired Size | 32768 |
| 28 | Spectral Size | 65536 |
| 29 | Digital Resolution | 0.35 |

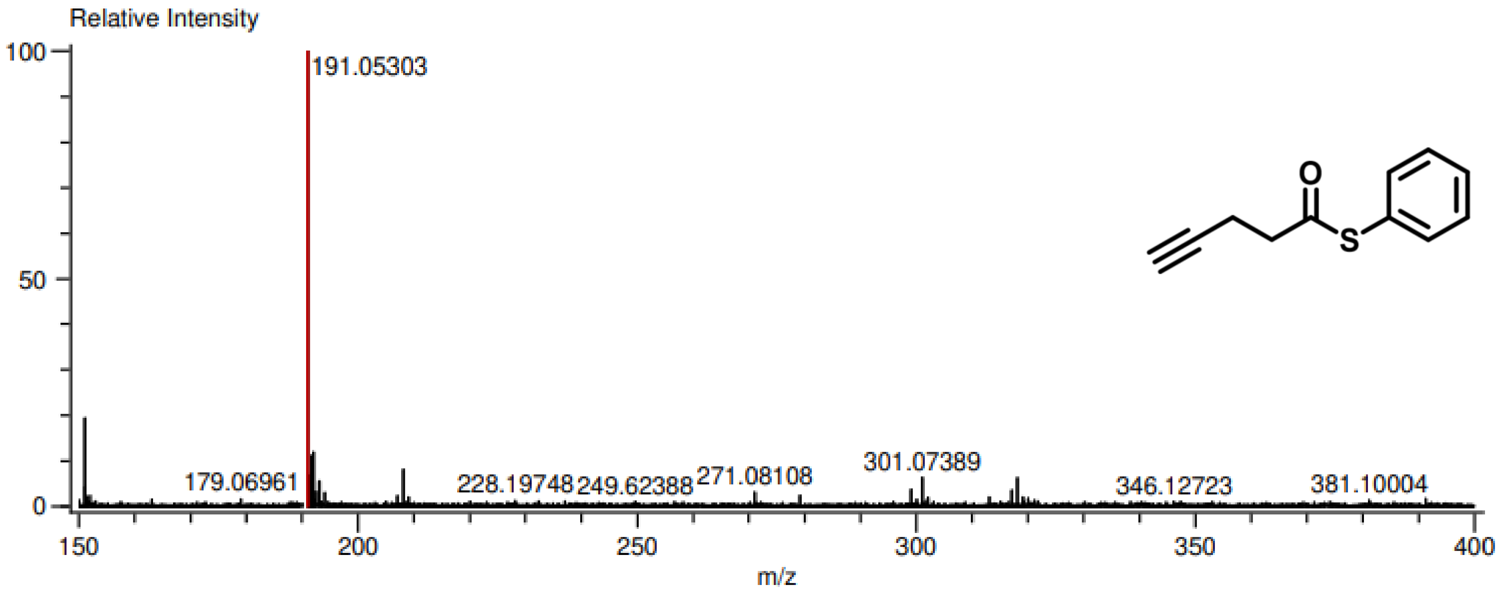


| Mass | Intensity | Calc. Mass | Mass Difference [mDa] | Mass Difference [ppm] | Possible Formula |
|---|---|---|---|---|---|
| 191.05303 | 50831.94 | 191.05306 | -0.03 | -0.13 | $^{12}C_{11}{}^{1}H_{11}{}^{16}O_{1}{}^{32}S_{1}$ |

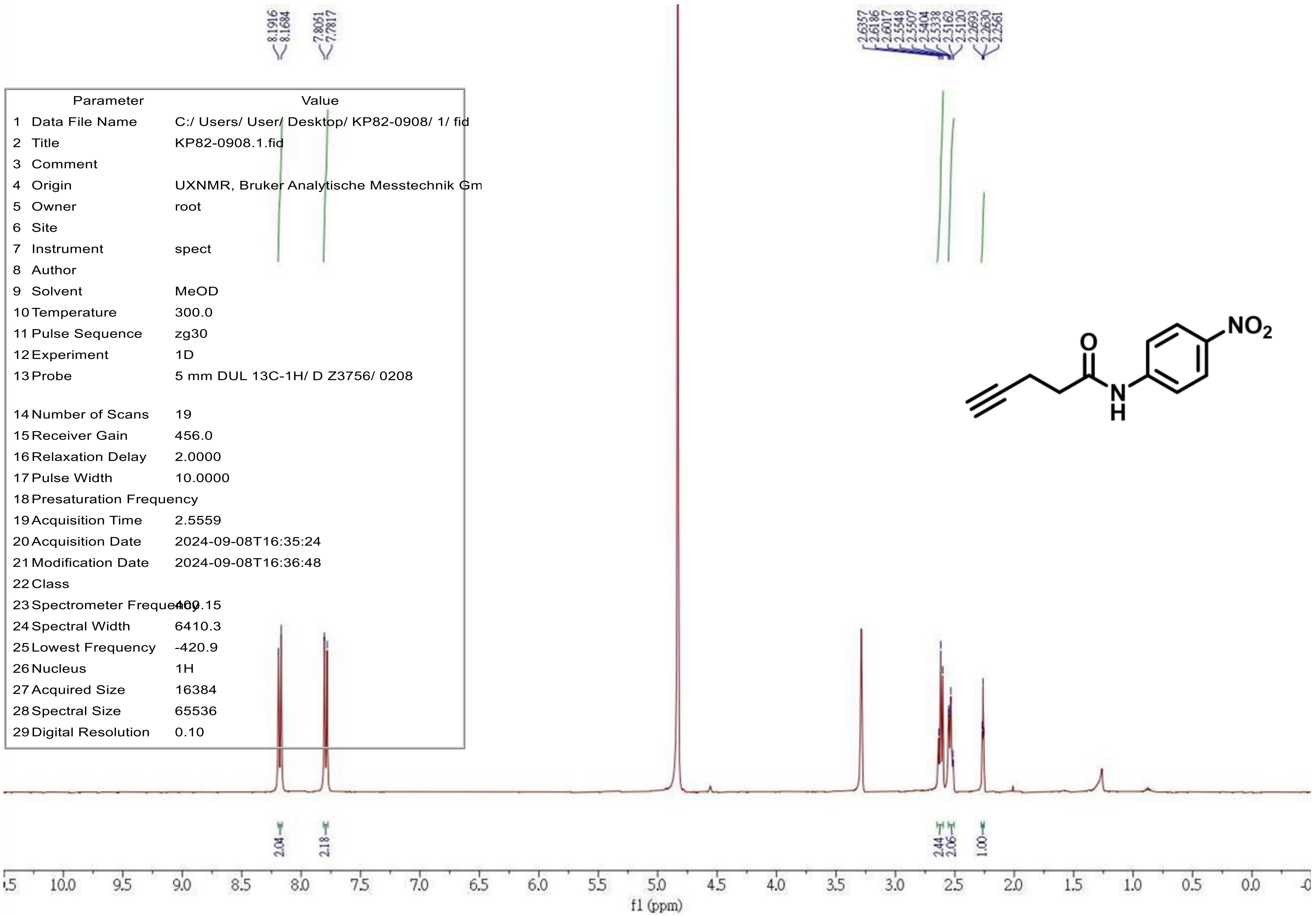


| | Parameter | Value |
|---|---|---|
| 1 | Data File Name | C:/ Users/ User/ Desktop/ KP82-0908/ 1/ fid |
| 2 | Title | KP82-0908.1.fid |
| 3 | Comment | |
| 4 | Origin | UXNMR, Bruker Analytische Messtechnik Gm |
| 5 | Owner | root |
| 6 | Site | |
| 7 | Instrument | spect |
| 8 | Author | |
| 9 | Solvent | MeOD |
| 10 | Temperature | 300.0 |
| 11 | Pulse Sequence | zg30 |
| 12 | Experiment | 1D |
| 13 | Probe | 5 mm DUL 13C-1H/ D Z3756/ 0208 |
| 14 | Number of Scans | 19 |
| 15 | Receiver Gain | 456.0 |
| 16 | Relaxation Delay | 2.0000 |
| 17 | Pulse Width | 10.0000 |
| 18 | Presaturation Frequency | |
| 19 | Acquisition Time | 2.5559 |
| 20 | Acquisition Date | 2024-09-08T16:35:24 |
| 21 | Modification Date | 2024-09-08T16:36:48 |
| 22 | Class | |
| 23 | Spectrometer Frequency | 400.15 |
| 24 | Spectral Width | 6410.3 |
| 25 | Lowest Frequency | -420.9 |
| 26 | Nucleus | 1H |
| 27 | Acquired Size | 16384 |
| 28 | Spectral Size | 65536 |
| 29 | Digital Resolution | 0.10 |

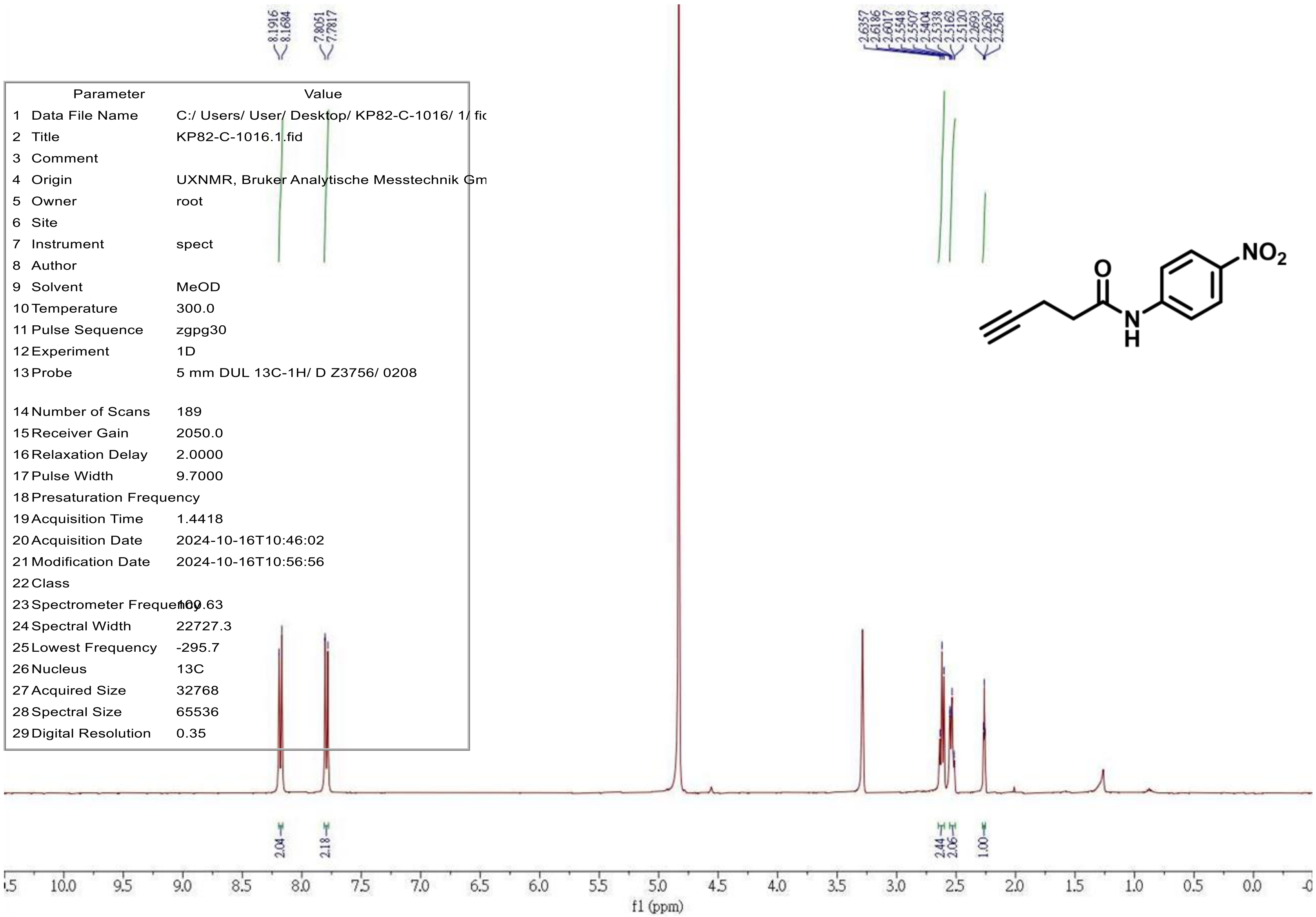


| Parameter | Value |
|---|---|
| 1 Data File Name | C:/ Users/ User/ Desktop/ KP82-C-1016/ 1/ fi[illegible] |
| 2 Title | KP82-C-1016.1.fid |
| 3 Comment | |
| 4 Origin | UXNMR, Bruker Analytische Messtechnik Gm[illegible] |
| 5 Owner | root |
| 6 Site | |
| 7 Instrument | spect |
| 8 Author | |
| 9 Solvent | MeOD |
| 10 Temperature | 300.0 |
| 11 Pulse Sequence | zgpg30 |
| 12 Experiment | 1D |
| 13 Probe | 5 mm DUL 13C-1H/ D Z3756/ 0208 |
| 14 Number of Scans | 189 |
| 15 Receiver Gain | 2050.0 |
| 16 Relaxation Delay | 2.0000 |
| 17 Pulse Width | 9.7000 |
| 18 Presaturation Frequency | |
| 19 Acquisition Time | 1.4418 |
| 20 Acquisition Date | 2024-10-16T10:46:02 |
| 21 Modification Date | 2024-10-16T10:56:56 |
| 22 Class | |
| 23 Spectrometer Frequency | 100.63 |
| 24 Spectral Width | 22727.3 |
| 25 Lowest Frequency | -295.7 |
| 26 Nucleus | 13C |
| 27 Acquired Size | 32768 |
| 28 Spectral Size | 65536 |
| 29 Digital Resolution | 0.35 |

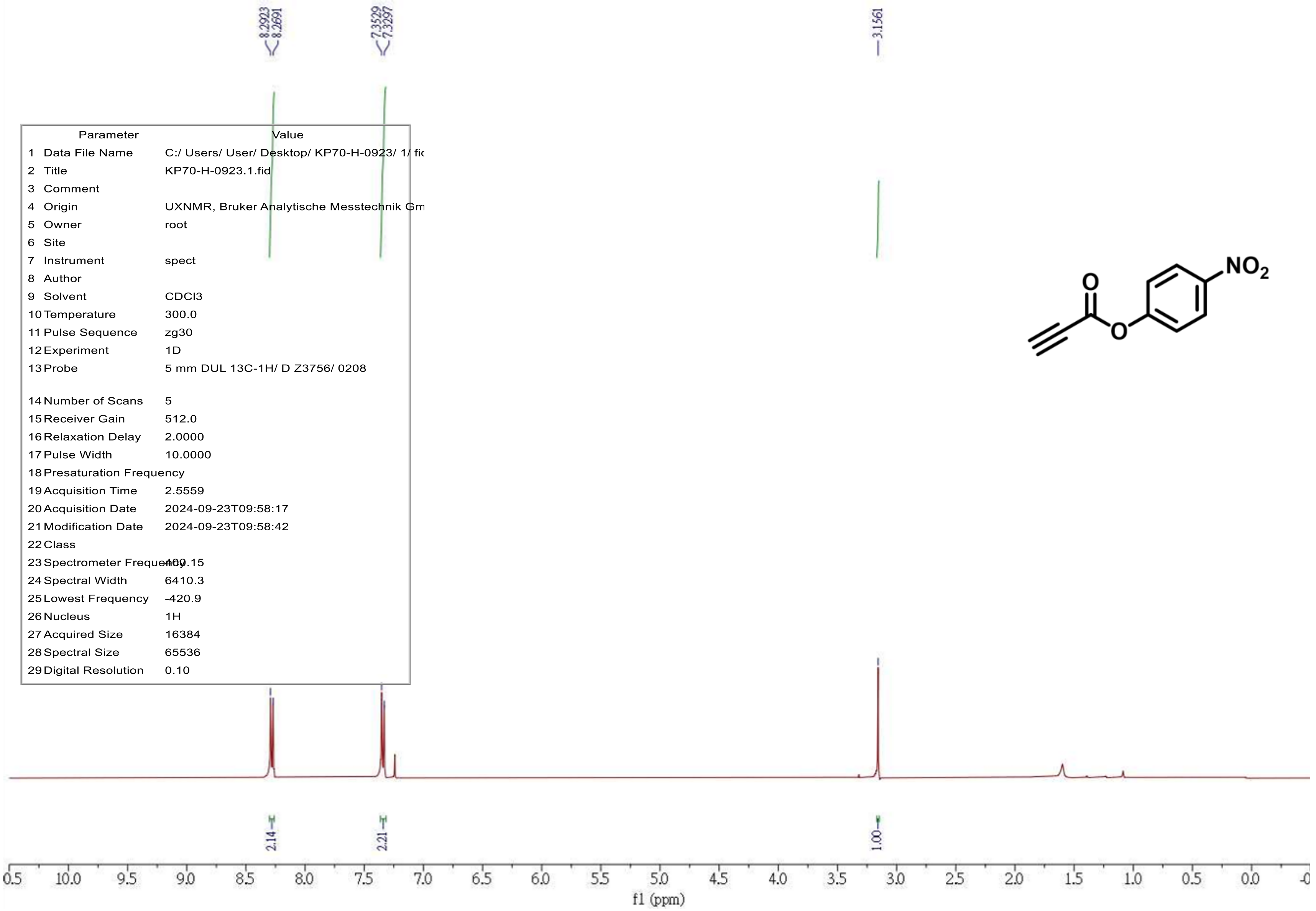


| | Parameter | Value |
|---|---|---|
| 1 | Data File Name | C:/ Users/ User/ Desktop/ KP70-H-0923/ 1/ fic |
| 2 | Title | KP70-H-0923.1.fid |
| 3 | Comment | |
| 4 | Origin | UXNMR, Bruker Analytische Messtechnik Gm |
| 5 | Owner | root |
| 6 | Site | |
| 7 | Instrument | spect |
| 8 | Author | |
| 9 | Solvent | CDCl3 |
| 10 | Temperature | 300.0 |
| 11 | Pulse Sequence | zg30 |
| 12 | Experiment | 1D |
| 13 | Probe | 5 mm DUL 13C-1H/ D Z3756/ 0208 |
| 14 | Number of Scans | 5 |
| 15 | Receiver Gain | 512.0 |
| 16 | Relaxation Delay | 2.0000 |
| 17 | Pulse Width | 10.0000 |
| 18 | Presaturation Frequency | |
| 19 | Acquisition Time | 2.5559 |
| 20 | Acquisition Date | 2024-09-23T09:58:17 |
| 21 | Modification Date | 2024-09-23T09:58:42 |
| 22 | Class | |
| 23 | Spectrometer Frequency | 400.15 |
| 24 | Spectral Width | 6410.3 |
| 25 | Lowest Frequency | -420.9 |
| 26 | Nucleus | 1H |
| 27 | Acquired Size | 16384 |
| 28 | Spectral Size | 65536 |
| 29 | Digital Resolution | 0.10 |

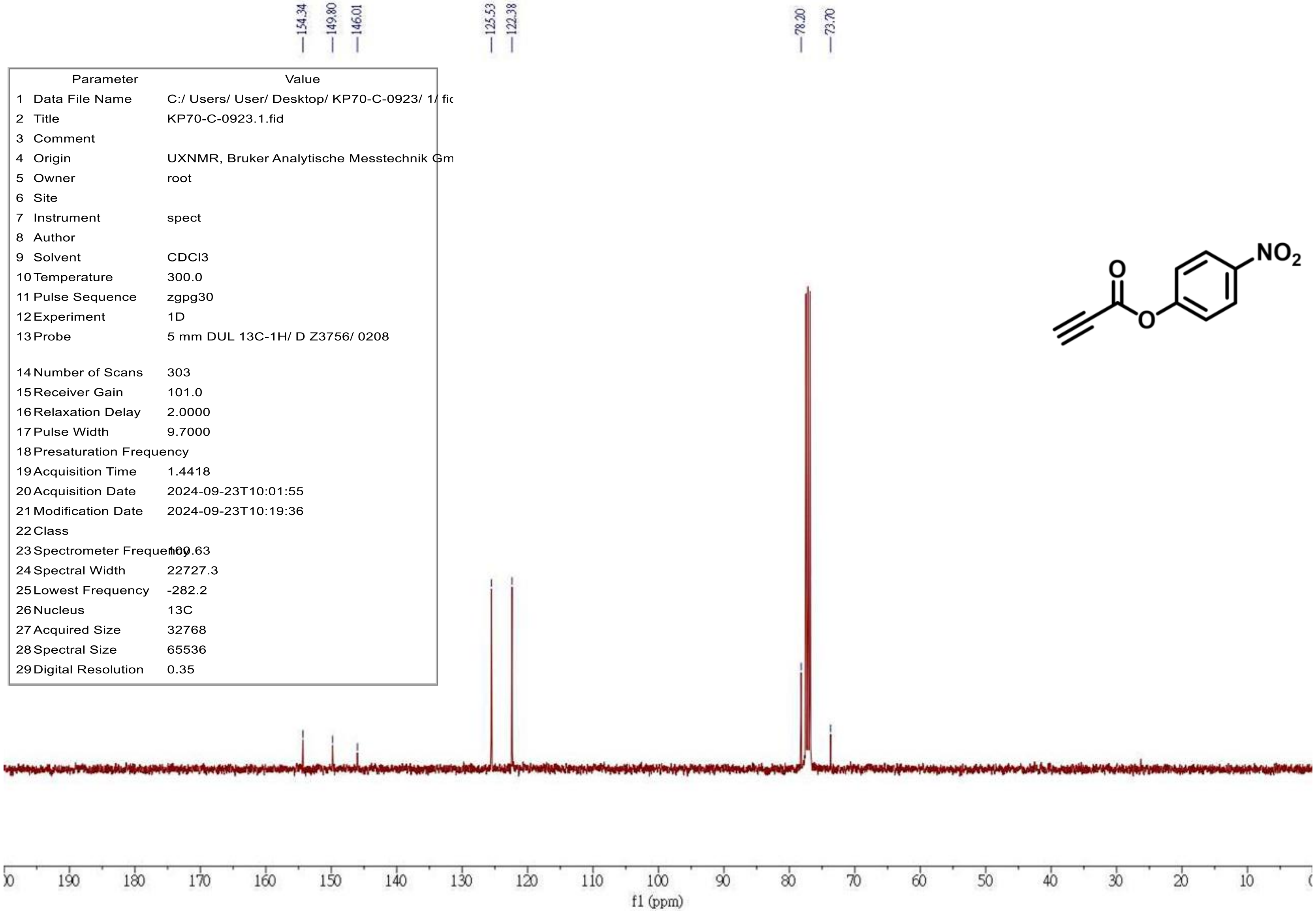
154.34
149.80
146.01
125.53
122.38
78.20
73.70
Parameter Value
1 Data File Name C:/ Users/ User/ Desktop/ KP70-C-0923/ 1/ fic
2 Title KP70-C-0923.1.fid
3 Comment
4 Origin UXNMR, Bruker Analytische Messtechnik Gm
5 Owner root
6 Site
7 Instrument spect
8 Author
9 Solvent CDCl3
10 Temperature 300.0
11 Pulse Sequence zgpg30
12 Experiment 1D
13 Probe 5 mm DUL 13C-1H/ D Z3756/ 0208
14 Number of Scans 303
15 Receiver Gain 101.0
16 Relaxation Delay 2.0000
17 Pulse Width 9.7000
18 Presaturation Frequency
19 Acquisition Time 1.4418
20 Acquisition Date 2024-09-23T10:01:55
21 Modification Date 2024-09-23T10:19:36
22 Class
23 Spectrometer Frequency 100.63
24 Spectral Width 22727.3
25 Lowest Frequency -282.2
26 Nucleus 13C
27 Acquired Size 32768
28 Spectral Size 65536
29 Digital Resolution 0.35
f1 (ppm)
NO2
O
O

| | Parameter | Value |
|---|---|---|
| 1 | Data File Name | C:/ Users/ User/ Desktop/ 產物NMR/ KP62-2-1/ 1 |
| 2 | Title | KP62-2-1.1.fid |
| 3 | Comment | |
| 4 | Origin | UXNMR, Bruker Analytische Messtechnik GmbH |
| 5 | Owner | root |
| 6 | Site | |
| 7 | Instrument | spect |
| 8 | Author | |
| 9 | Solvent | CDCl3 |
| 10 | Temperature | 300.0 |
| 11 | Pulse Sequence | zg30 |
| 12 | Experiment | 1D |
| 13 | Probe | 5 mm DUL 13C-1H/ D Z3756/ 0208 |
| 14 | Number of Scans | 17 |
| 15 | Receiver Gain | 144.0 |
| 16 | Relaxation Delay | 2.0000 |
| 17 | Pulse Width | 10.0000 |
| 18 | Presaturation Frequency | |
| 19 | Acquisition Time | 2.5559 |
| 20 | Acquisition Date | 2024-05-16T13:34:07 |
| 21 | Modification Date | 2024-05-16T13:35:08 |
| 22 | Class | |
| 23 | Spectrometer Frequency | 400.15 |
| 24 | Spectral Width | 6410.3 |
| 25 | Lowest Frequency | -420.9 |
| 26 | Nucleus | 1H |
| 27 | Acquired Size | 16384 |
| 28 | Spectral Size | 65536 |
| 29 | Digital Resolution | 0.10 |

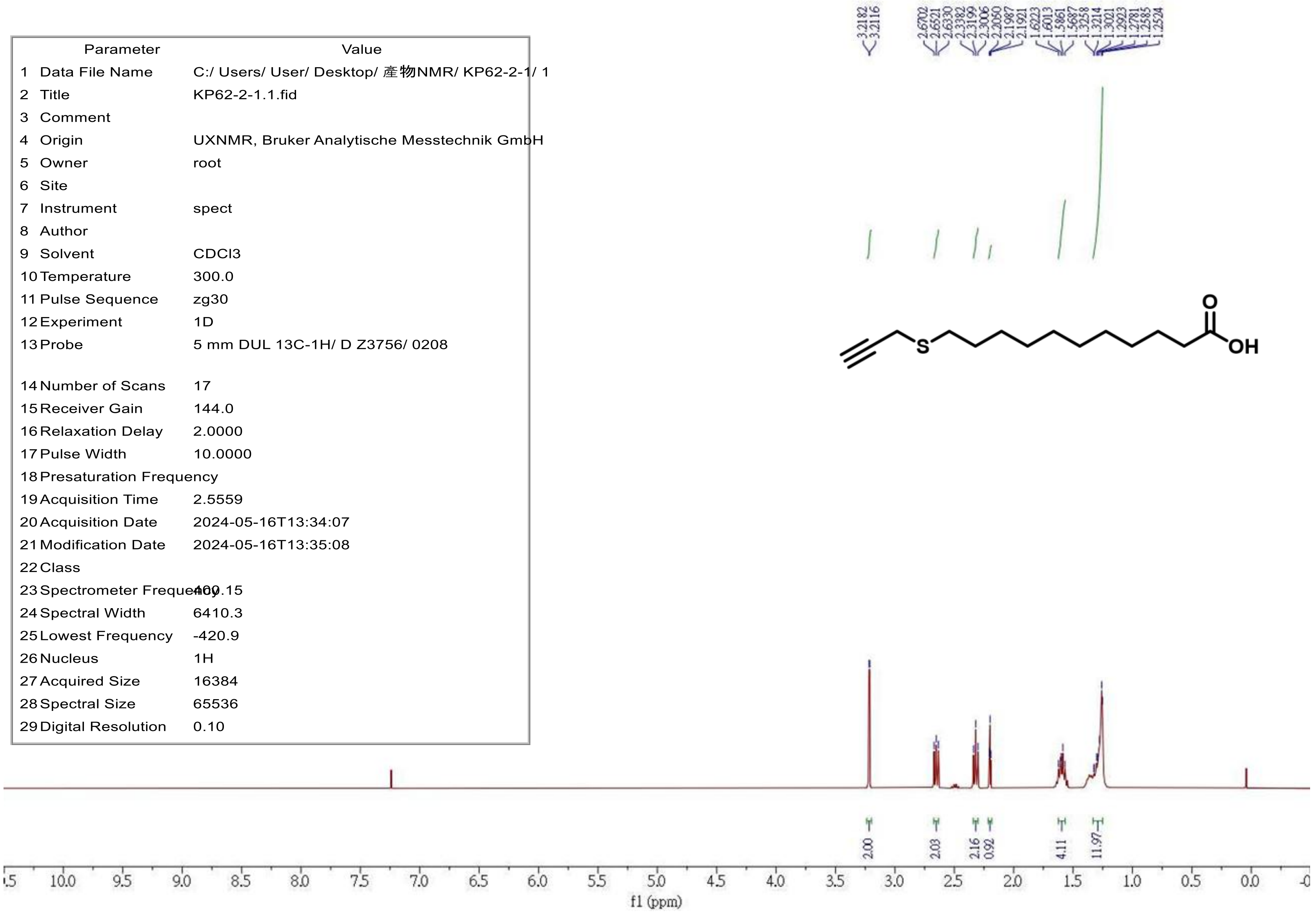

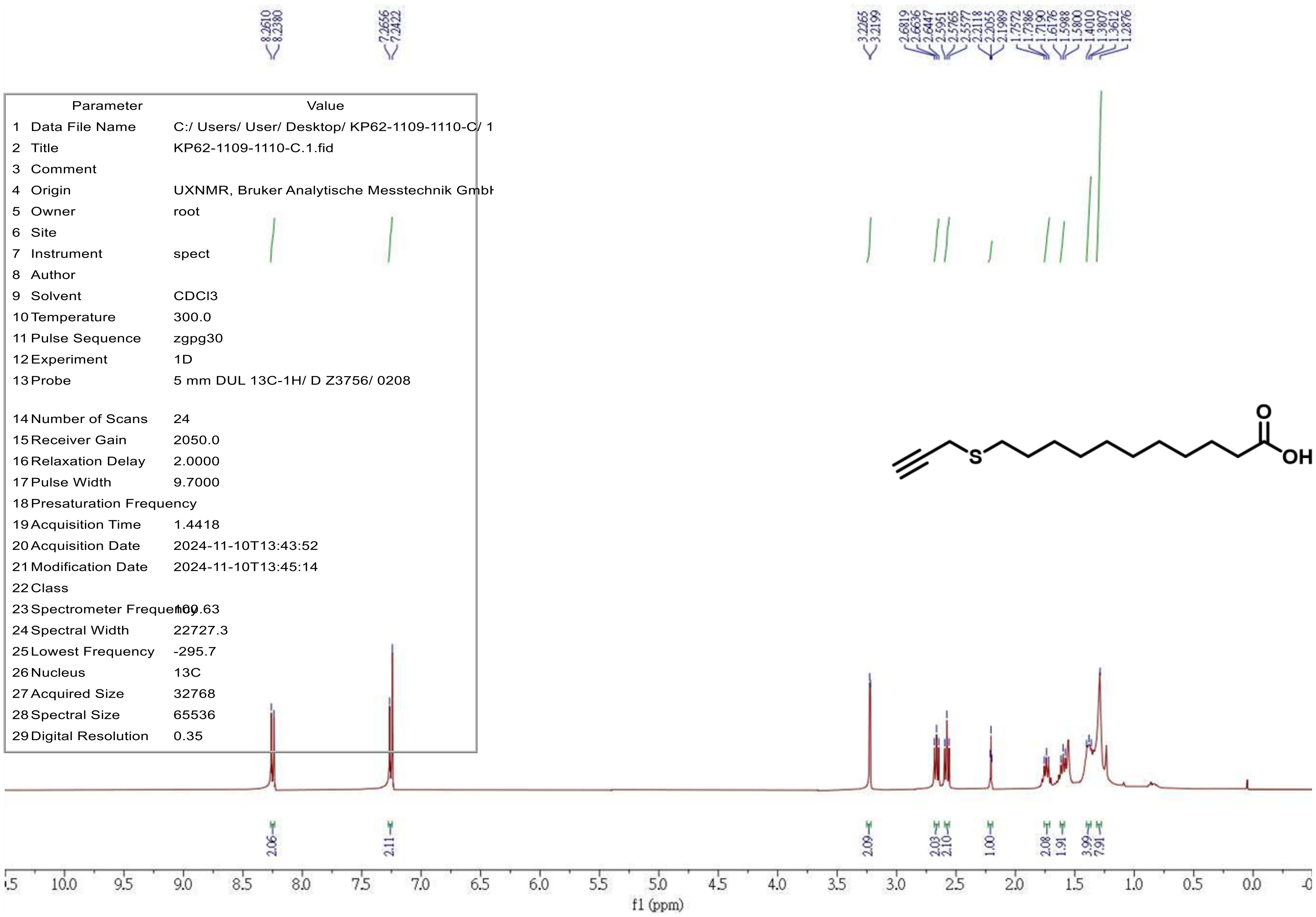


| | Parameter | Value |
|---|---|---|
| 1 | Data File Name | C:/ Users/ User/ Desktop/ KP62-1109-1110-C/ 1 |
| 2 | Title | KP62-1109-1110-C.1.fid |
| 3 | Comment | |
| 4 | Origin | UXNMR, Bruker Analytische Messtechnik GmbH |
| 5 | Owner | root |
| 6 | Site | |
| 7 | Instrument | spect |
| 8 | Author | |
| 9 | Solvent | CDCl3 |
| 10 | Temperature | 300.0 |
| 11 | Pulse Sequence | zgpg30 |
| 12 | Experiment | 1D |
| 13 | Probe | 5 mm DUL 13C-1H/ D Z3756/ 0208 |
| 14 | Number of Scans | 24 |
| 15 | Receiver Gain | 2050.0 |
| 16 | Relaxation Delay | 2.0000 |
| 17 | Pulse Width | 9.7000 |
| 18 | Presaturation Frequency | |
| 19 | Acquisition Time | 1.4418 |
| 20 | Acquisition Date | 2024-11-10T13:43:52 |
| 21 | Modification Date | 2024-11-10T13:45:14 |
| 22 | Class | |
| 23 | Spectrometer Frequency | 100.63 |
| 24 | Spectral Width | 22727.3 |
| 25 | Lowest Frequency | -295.7 |
| 26 | Nucleus | 13C |
| 27 | Acquired Size | 32768 |
| 28 | Spectral Size | 65536 |
| 29 | Digital Resolution | 0.35 |

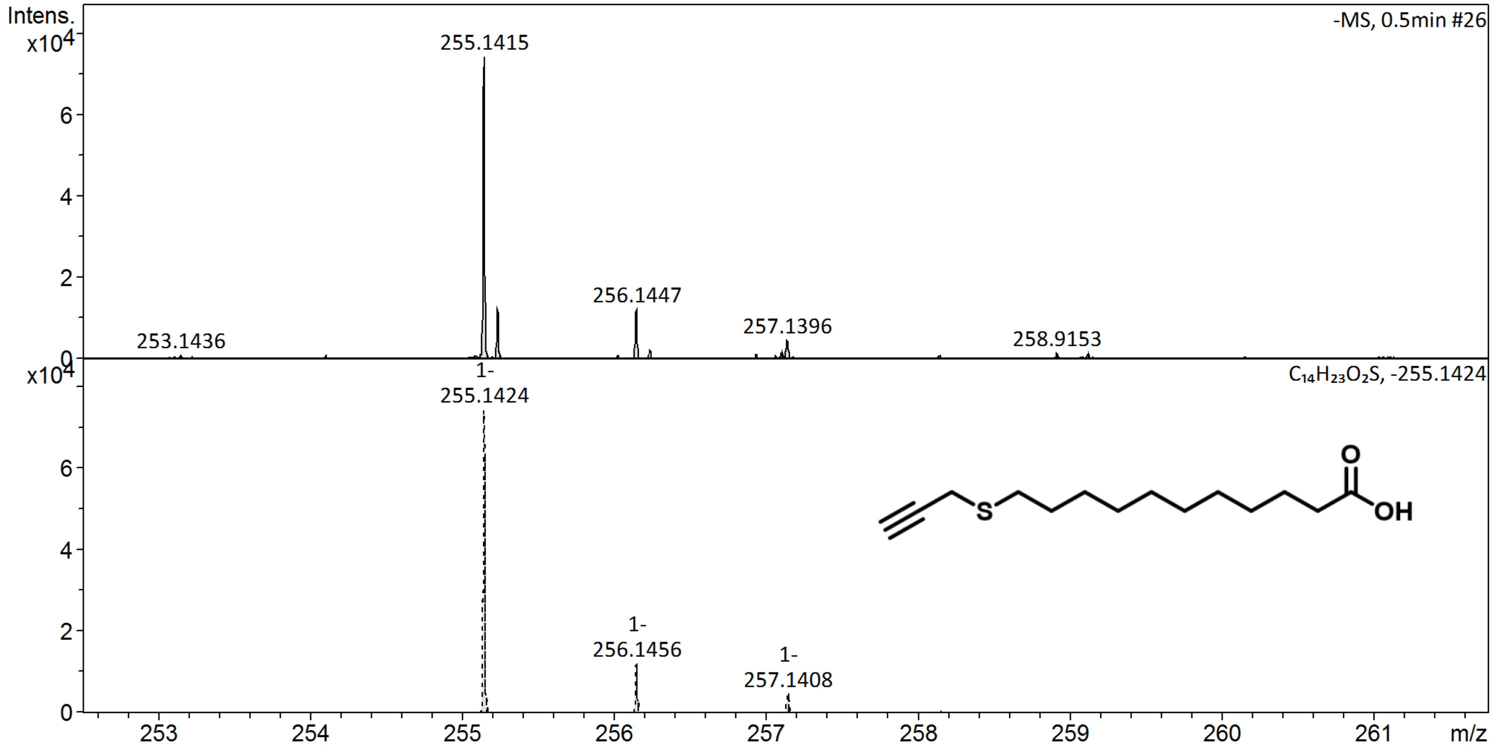


# Display Report

| Meas. m/z | # | Ion Formula | m/z | err [ppm] | mSigma | # Sigma | Score | rdb | e⁻ Conf | N-Rule | Adduct |
|---|---|---|---|---|---|---|---|---|---|---|---|
| 255.1415 | 1 | C14H23O2S | 255.1424 | 3.5 | 1.6 | 1 | 100.00 | 3.5 | even | ok | M-H |

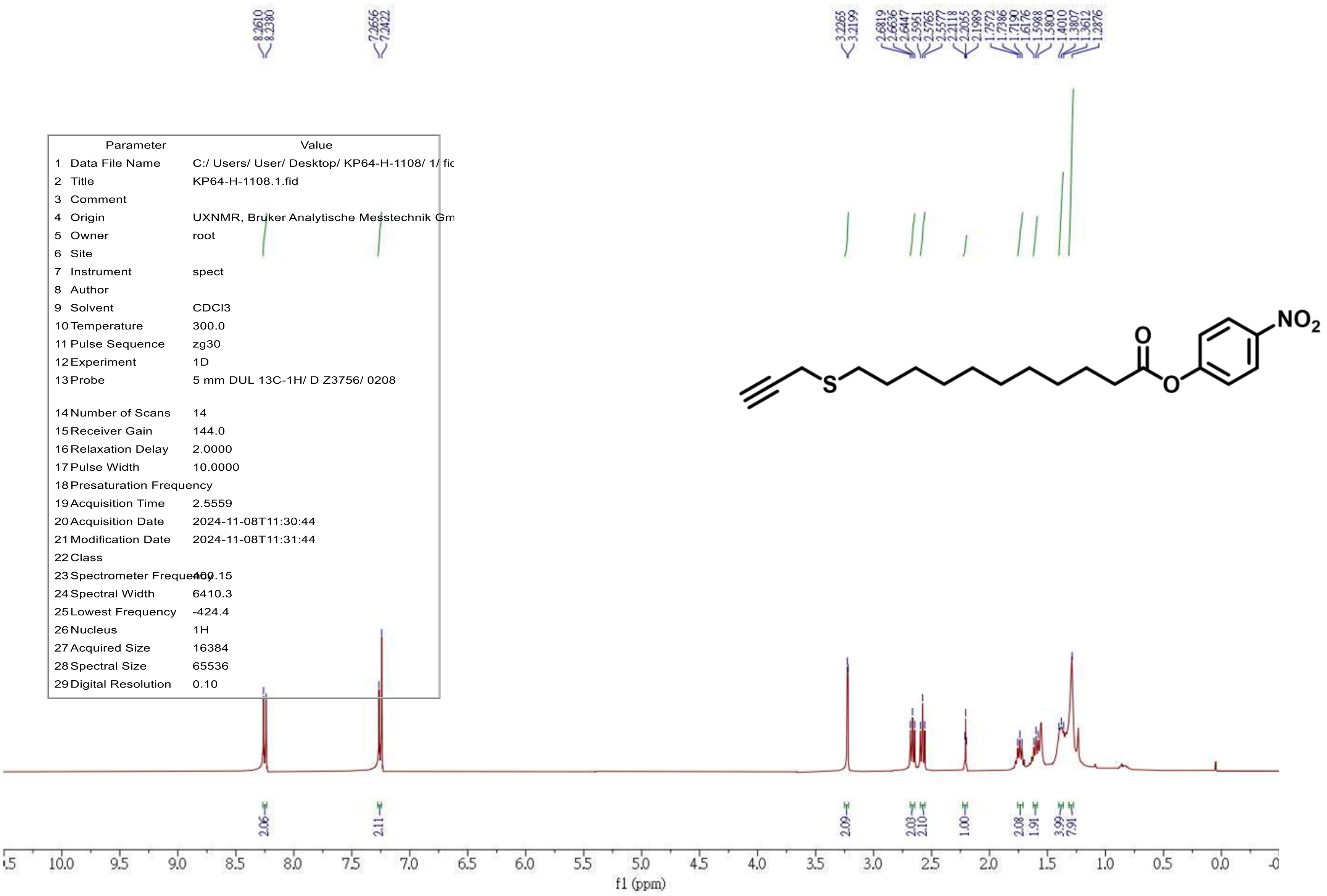


| | Parameter | Value |
|---|---|---|
| 1 | Data File Name | C:/ Users/ User/ Desktop/ KP64-H-1108/ 1/ fic |
| 2 | Title | KP64-H-1108.1.fid |
| 3 | Comment | |
| 4 | Origin | UXNMR, Bruker Analytische Messtechnik Gm |
| 5 | Owner | root |
| 6 | Site | |
| 7 | Instrument | spect |
| 8 | Author | |
| 9 | Solvent | CDCl3 |
| 10 | Temperature | 300.0 |
| 11 | Pulse Sequence | zg30 |
| 12 | Experiment | 1D |
| 13 | Probe | 5 mm DUL 13C-1H/ D Z3756/ 0208 |
| 14 | Number of Scans | 14 |
| 15 | Receiver Gain | 144.0 |
| 16 | Relaxation Delay | 2.0000 |
| 17 | Pulse Width | 10.0000 |
| 18 | Presaturation Frequency | |
| 19 | Acquisition Time | 2.5559 |
| 20 | Acquisition Date | 2024-11-08T11:30:44 |
| 21 | Modification Date | 2024-11-08T11:31:44 |
| 22 | Class | |
| 23 | Spectrometer Frequency | 400.15 |
| 24 | Spectral Width | 6410.3 |
| 25 | Lowest Frequency | -424.4 |
| 26 | Nucleus | 1H |
| 27 | Acquired Size | 16384 |
| 28 | Spectral Size | 65536 |
| 29 | Digital Resolution | 0.10 |

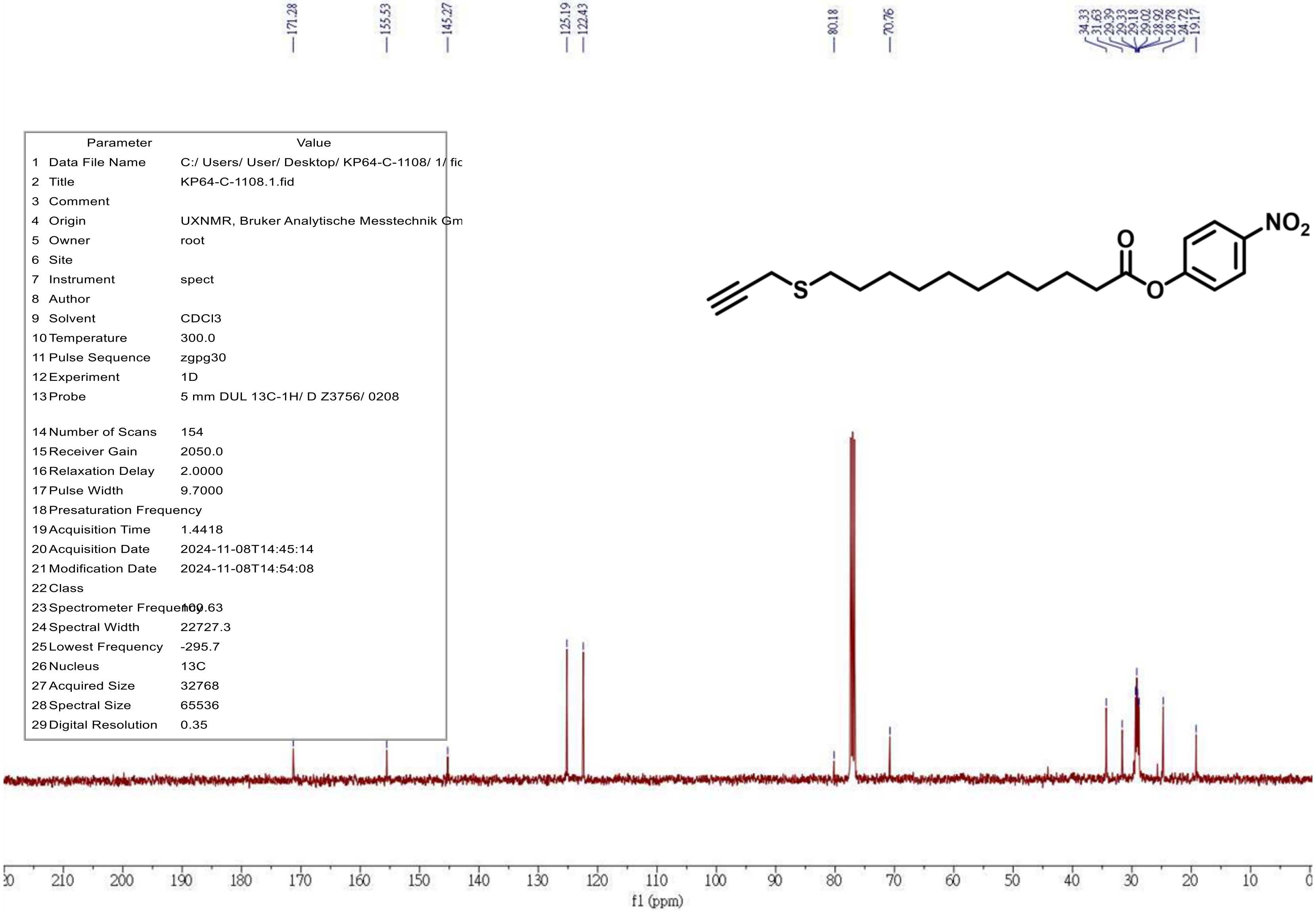


| | Parameter | Value |
|---|---|---|
| 1 | Data File Name | C:/ Users/ User/ Desktop/ KP64-C-1108/ 1/ fic |
| 2 | Title | KP64-C-1108.1.fid |
| 3 | Comment | |
| 4 | Origin | UXNMR, Bruker Analytische Messtechnik Gm |
| 5 | Owner | root |
| 6 | Site | |
| 7 | Instrument | spect |
| 8 | Author | |
| 9 | Solvent | CDCl3 |
| 10 | Temperature | 300.0 |
| 11 | Pulse Sequence | zgpg30 |
| 12 | Experiment | 1D |
| 13 | Probe | 5 mm DUL 13C-1H/ D Z3756/ 0208 |
| 14 | Number of Scans | 154 |
| 15 | Receiver Gain | 2050.0 |
| 16 | Relaxation Delay | 2.0000 |
| 17 | Pulse Width | 9.7000 |
| 18 | Presaturation Frequency | |
| 19 | Acquisition Time | 1.4418 |
| 20 | Acquisition Date | 2024-11-08T14:45:14 |
| 21 | Modification Date | 2024-11-08T14:54:08 |
| 22 | Class | |
| 23 | Spectrometer Frequency | 100.63 |
| 24 | Spectral Width | 22727.3 |
| 25 | Lowest Frequency | -295.7 |
| 26 | Nucleus | 13C |
| 27 | Acquired Size | 32768 |
| 28 | Spectral Size | 65536 |
| 29 | Digital Resolution | 0.35 |

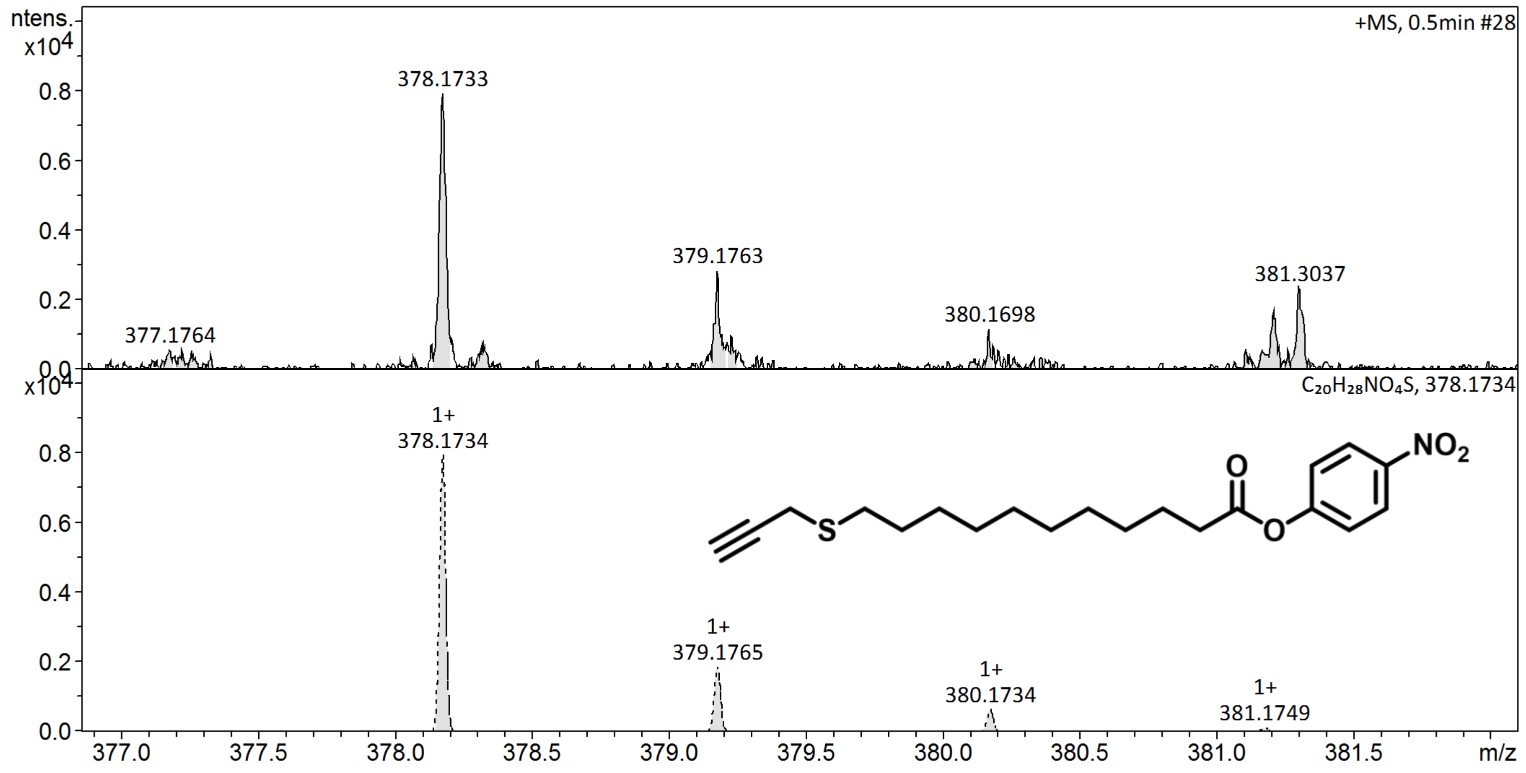


# Display Report

| Meas. m/z | # | Ion Formula | m/z | err [ppm] | mSigma | # Sigma | Score | rdb | e⁻ Conf | N-Rule | Adduct |
|---|---|---|---|---|---|---|---|---|---|---|---|
| 378.1733 | 1 | C20H28NO4S | 378.1734 | 0.2 | 74.4 | 1 | 100.00 | 7.5 | even | ok | M+H |

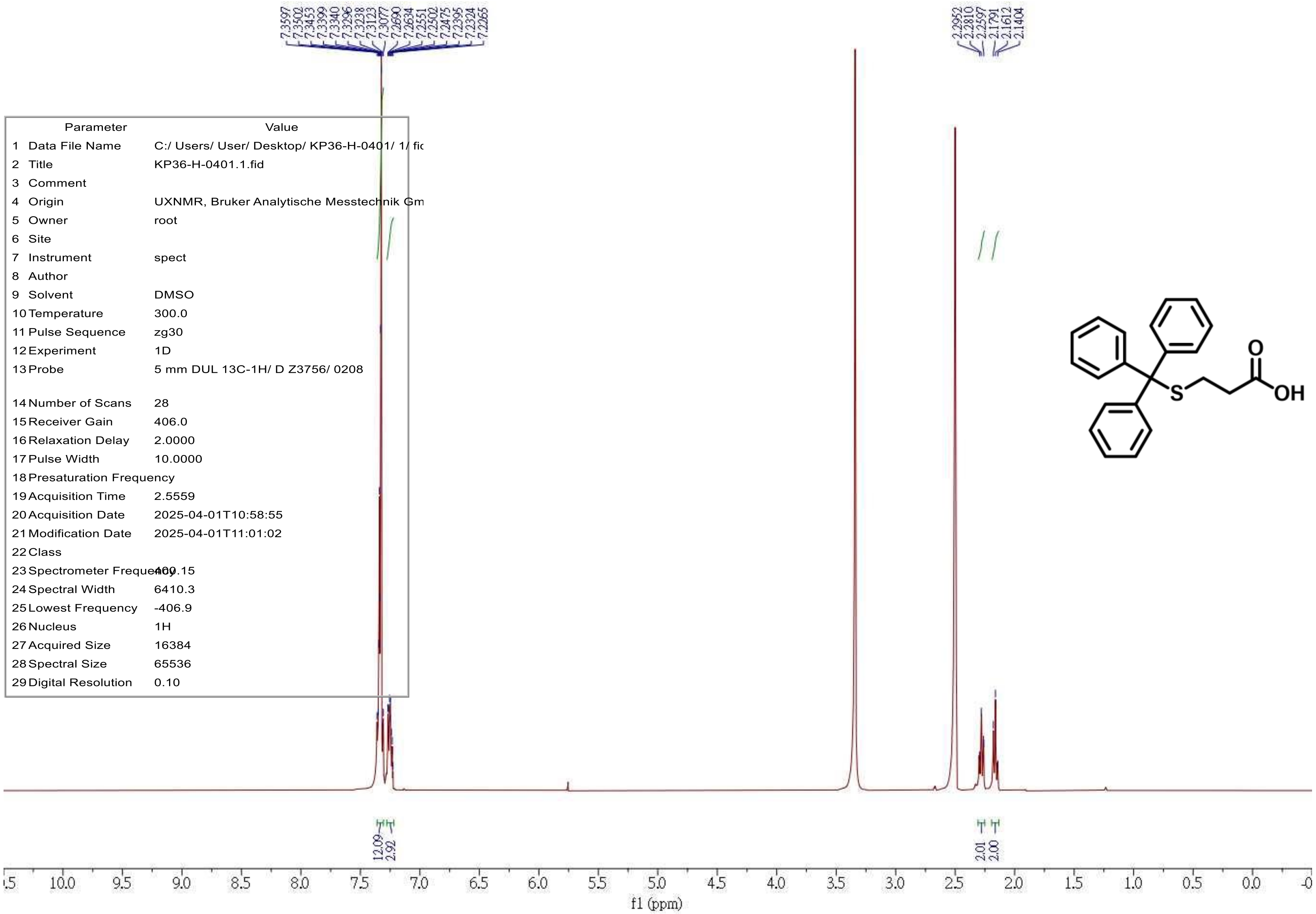


| | Parameter | Value |
|---|---|---|
| 1 | Data File Name | C:/ Users/ User/ Desktop/ KP36-H-0401/ 1/ fic |
| 2 | Title | KP36-H-0401.1.fid |
| 3 | Comment | |
| 4 | Origin | UXNMR, Bruker Analytische Messtechnik Gm |
| 5 | Owner | root |
| 6 | Site | |
| 7 | Instrument | spect |
| 8 | Author | |
| 9 | Solvent | DMSO |
| 10 | Temperature | 300.0 |
| 11 | Pulse Sequence | zg30 |
| 12 | Experiment | 1D |
| 13 | Probe | 5 mm DUL 13C-1H/ D Z3756/ 0208 |
| 14 | Number of Scans | 28 |
| 15 | Receiver Gain | 406.0 |
| 16 | Relaxation Delay | 2.0000 |
| 17 | Pulse Width | 10.0000 |
| 18 | Presaturation Frequency | |
| 19 | Acquisition Time | 2.5559 |
| 20 | Acquisition Date | 2025-04-01T10:58:55 |
| 21 | Modification Date | 2025-04-01T11:01:02 |
| 22 | Class | |
| 23 | Spectrometer Frequency | 400.15 |
| 24 | Spectral Width | 6410.3 |
| 25 | Lowest Frequency | -406.9 |
| 26 | Nucleus | 1H |
| 27 | Acquired Size | 16384 |
| 28 | Spectral Size | 65536 |
| 29 | Digital Resolution | 0.10 |

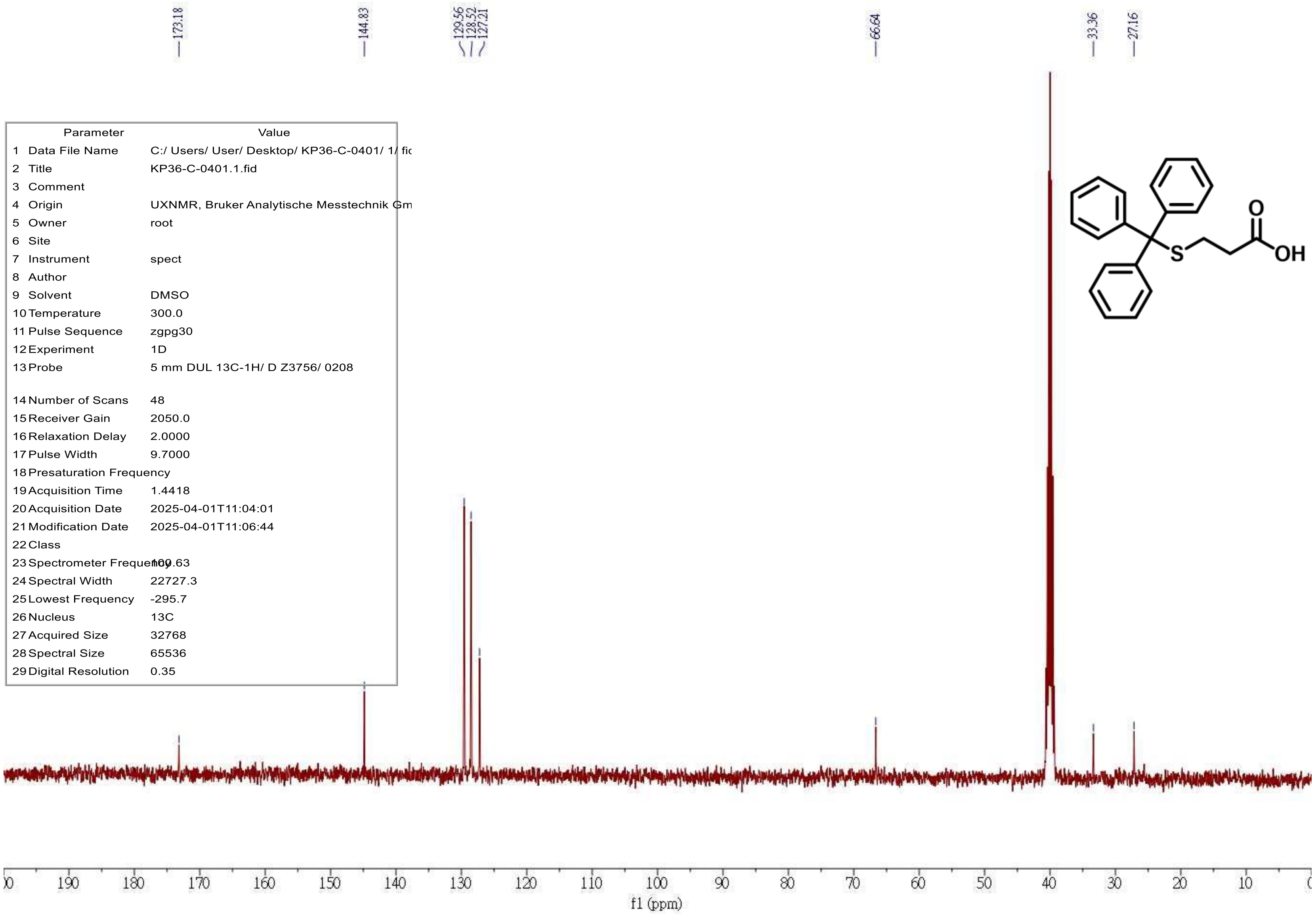


| Parameter | Value |
|---|---|
| 1 Data File Name | C:/ Users/ User/ Desktop/ KP36-C-0401/ 1/ fi |
| 2 Title | KP36-C-0401.1.fid |
| 3 Comment | |
| 4 Origin | UXNMR, Bruker Analytische Messtechnik Gm |
| 5 Owner | root |
| 6 Site | |
| 7 Instrument | spect |
| 8 Author | |
| 9 Solvent | DMSO |
| 10 Temperature | 300.0 |
| 11 Pulse Sequence | zgpg30 |
| 12 Experiment | 1D |
| 13 Probe | 5 mm DUL 13C-1H/ D Z3756/ 0208 |
| 14 Number of Scans | 48 |
| 15 Receiver Gain | 2050.0 |
| 16 Relaxation Delay | 2.0000 |
| 17 Pulse Width | 9.7000 |
| 18 Presaturation Frequency | |
| 19 Acquisition Time | 1.4418 |
| 20 Acquisition Date | 2025-04-01T11:04:01 |
| 21 Modification Date | 2025-04-01T11:06:44 |
| 22 Class | |
| 23 Spectrometer Frequency | 100.63 |
| 24 Spectral Width | 22727.3 |
| 25 Lowest Frequency | -295.7 |
| 26 Nucleus | 13C |
| 27 Acquired Size | 32768 |
| 28 Spectral Size | 65536 |
| 29 Digital Resolution | 0.35 |

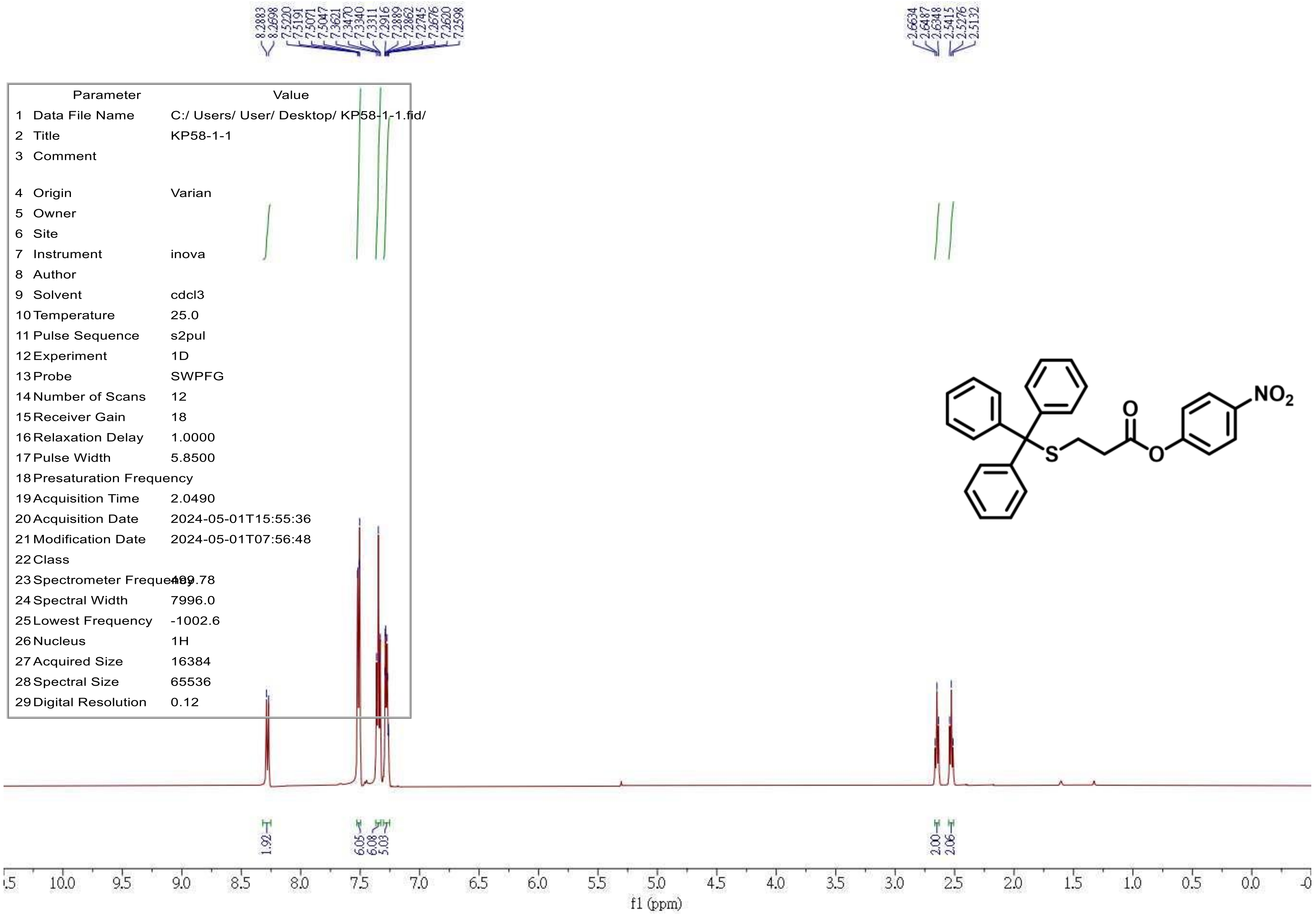


| | Parameter | Value |
|---|---|---|
| 1 | Data File Name | C:/ Users/ User/ Desktop/ KP58-1-1.fid/ |
| 2 | Title | KP58-1-1 |
| 3 | Comment | |
| 4 | Origin | Varian |
| 5 | Owner | |
| 6 | Site | |
| 7 | Instrument | inova |
| 8 | Author | |
| 9 | Solvent | cdcl3 |
| 10 | Temperature | 25.0 |
| 11 | Pulse Sequence | s2pul |
| 12 | Experiment | 1D |
| 13 | Probe | SWPFG |
| 14 | Number of Scans | 12 |
| 15 | Receiver Gain | 18 |
| 16 | Relaxation Delay | 1.0000 |
| 17 | Pulse Width | 5.8500 |
| 18 | Presaturation Frequency | |
| 19 | Acquisition Time | 2.0490 |
| 20 | Acquisition Date | 2024-05-01T15:55:36 |
| 21 | Modification Date | 2024-05-01T07:56:48 |
| 22 | Class | |
| 23 | Spectrometer Frequency | 499.78 |
| 24 | Spectral Width | 7996.0 |
| 25 | Lowest Frequency | -1002.6 |
| 26 | Nucleus | 1H |
| 27 | Acquired Size | 16384 |
| 28 | Spectral Size | 65536 |
| 29 | Digital Resolution | 0.12 |

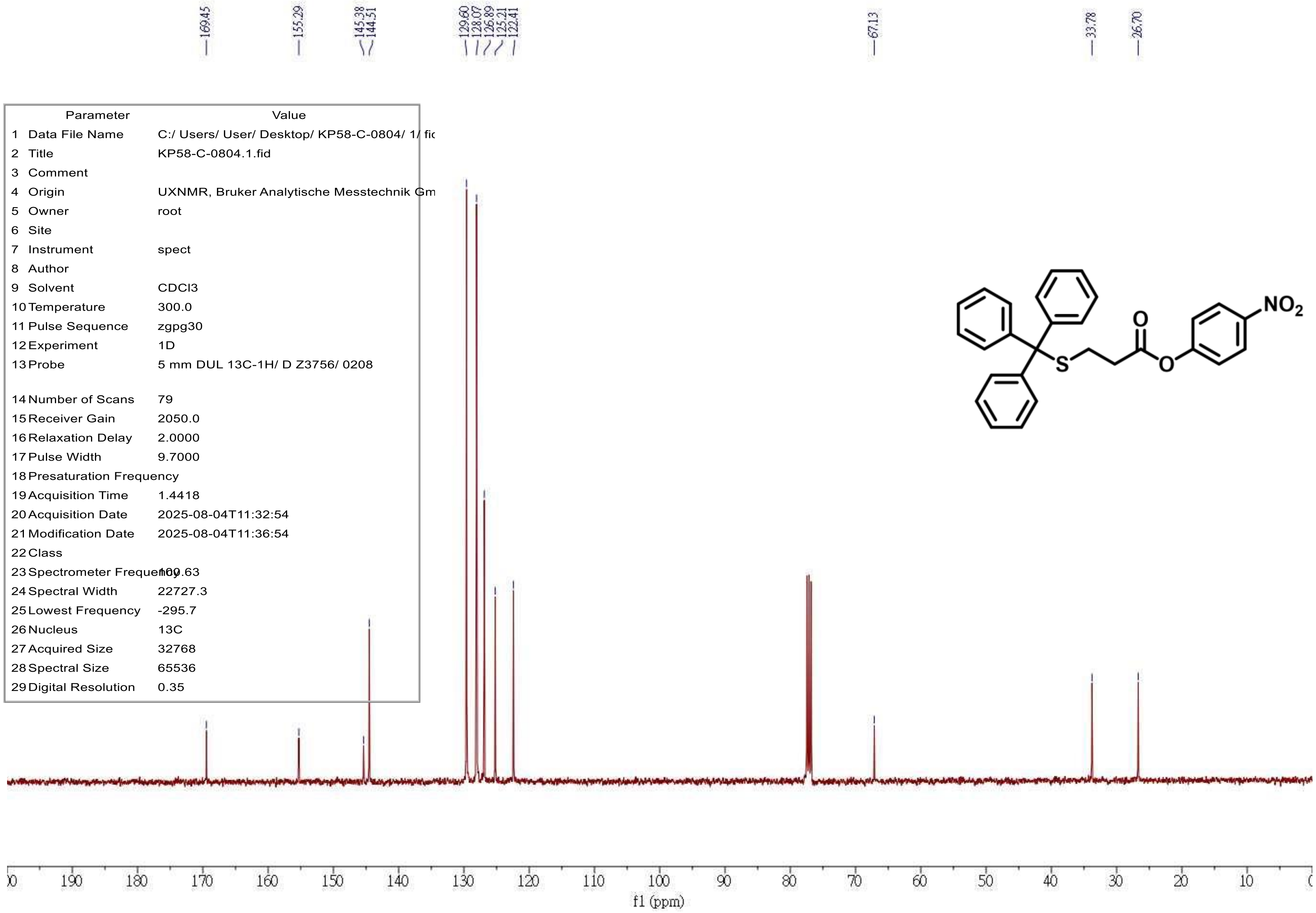


| | Parameter | Value |
|---|---|---|
| 1 | Data File Name | C:/ Users/ User/ Desktop/ KP58-C-0804/ 1/ fic |
| 2 | Title | KP58-C-0804.1.fid |
| 3 | Comment | |
| 4 | Origin | UXNMR, Bruker Analytische Messtechnik Gm |
| 5 | Owner | root |
| 6 | Site | |
| 7 | Instrument | spect |
| 8 | Author | |
| 9 | Solvent | CDCl3 |
| 10 | Temperature | 300.0 |
| 11 | Pulse Sequence | zgpg30 |
| 12 | Experiment | 1D |
| 13 | Probe | 5 mm DUL 13C-1H/ D Z3756/ 0208 |
| 14 | Number of Scans | 79 |
| 15 | Receiver Gain | 2050.0 |
| 16 | Relaxation Delay | 2.0000 |
| 17 | Pulse Width | 9.7000 |
| 18 | Presaturation Frequency | |
| 19 | Acquisition Time | 1.4418 |
| 20 | Acquisition Date | 2025-08-04T11:32:54 |
| 21 | Modification Date | 2025-08-04T11:36:54 |
| 22 | Class | |
| 23 | Spectrometer Frequency | 100.63 |
| 24 | Spectral Width | 22727.3 |
| 25 | Lowest Frequency | -295.7 |
| 26 | Nucleus | 13C |
| 27 | Acquired Size | 32768 |
| 28 | Spectral Size | 65536 |
| 29 | Digital Resolution | 0.35 |

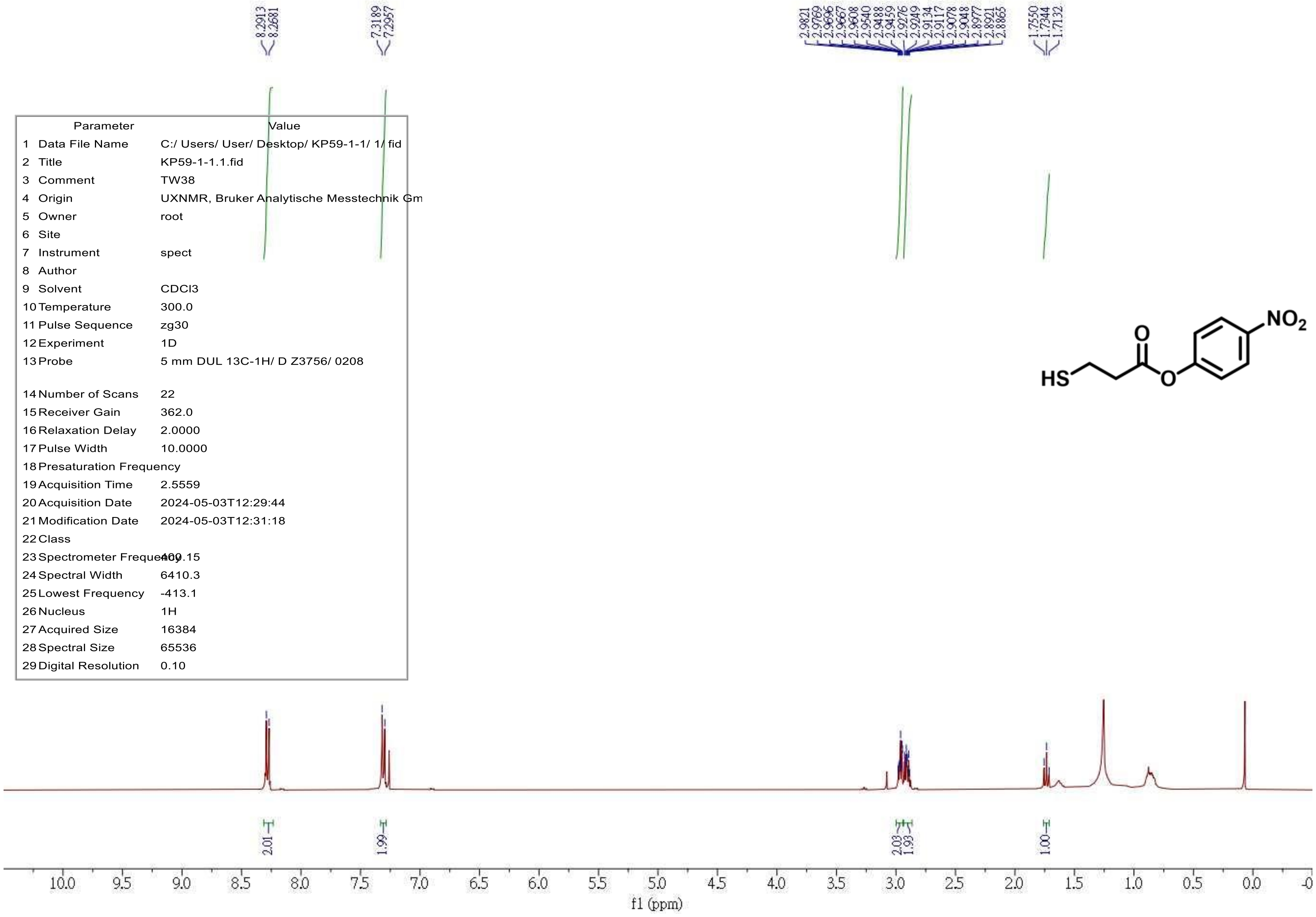


| | Parameter | Value |
|---|---|---|
| 1 | Data File Name | C:/ Users/ User/ Desktop/ KP59-1-1/ 1/ fid |
| 2 | Title | KP59-1-1.1.fid |
| 3 | Comment | TW38 |
| 4 | Origin | UXNMR, Bruker Analytische Messtechnik Gm |
| 5 | Owner | root |
| 6 | Site | |
| 7 | Instrument | spect |
| 8 | Author | |
| 9 | Solvent | CDCl3 |
| 10 | Temperature | 300.0 |
| 11 | Pulse Sequence | zg30 |
| 12 | Experiment | 1D |
| 13 | Probe | 5 mm DUL 13C-1H/ D Z3756/ 0208 |
| 14 | Number of Scans | 22 |
| 15 | Receiver Gain | 362.0 |
| 16 | Relaxation Delay | 2.0000 |
| 17 | Pulse Width | 10.0000 |
| 18 | Presaturation Frequency | |
| 19 | Acquisition Time | 2.5559 |
| 20 | Acquisition Date | 2024-05-03T12:29:44 |
| 21 | Modification Date | 2024-05-03T12:31:18 |
| 22 | Class | |
| 23 | Spectrometer Frequency | 400.15 |
| 24 | Spectral Width | 6410.3 |
| 25 | Lowest Frequency | -413.1 |
| 26 | Nucleus | 1H |
| 27 | Acquired Size | 16384 |
| 28 | Spectral Size | 65536 |
| 29 | Digital Resolution | 0.10 |
